# Efficient Synthesis of β-Aryl-γ-lactams and Their Resolution with (*S*)-Naproxen: Preparation of (*R*)- and (*S*)-Baclofen

**DOI:** 10.3390/molecules201219830

**Published:** 2015-12-10

**Authors:** Iris J. Montoya-Balbás, Berenice Valentín-Guevara, Estefanía López-Mendoza, Irma Linzaga-Elizalde, Mario Ordoñez, Perla Román-Bravo

**Affiliations:** Centro de Investigaciones Químicas CIQ-IICBA, Universidad Autónoma del Estado de Morelos, Av. Universidad 1001, 62209 Cuernavaca, Morelos, Mexico; natzelane_k@hotmail.com (I.J.M.-B.); b.valentinguevara@gmail.com (B.V.-G.); fa_pple@hotmail.com (E.L.-M.); palacios@uaem.mx (M.O.); rperl@uaem.mx (P.R.-B.)

**Keywords:** β-aryl-γ-lactams, Michael addition, resolution, (*S*)-naproxen, baclofen, phenibut

## Abstract

An efficient synthesis of enantiomerically-pure β-aryl-γ-lactams is described. The principal feature of this synthesis is the practical resolution of β-aryl-γ-lactams with (*S*)-Naproxen. The procedure is based on the Michael addition of nitromethane to benzylidenemalonates, which was easily obtained, followed by the reduction of the γ-nitroester in the presence of Raney nickel and the subsequent saponification/decarboxylation reaction. The utility of this methodology was highlighted by the preparation of enantiomerically-pure (*R*)- and (*S*)-Baclofen hydrochloride.

## 1. Introduction

**Figure 1:**
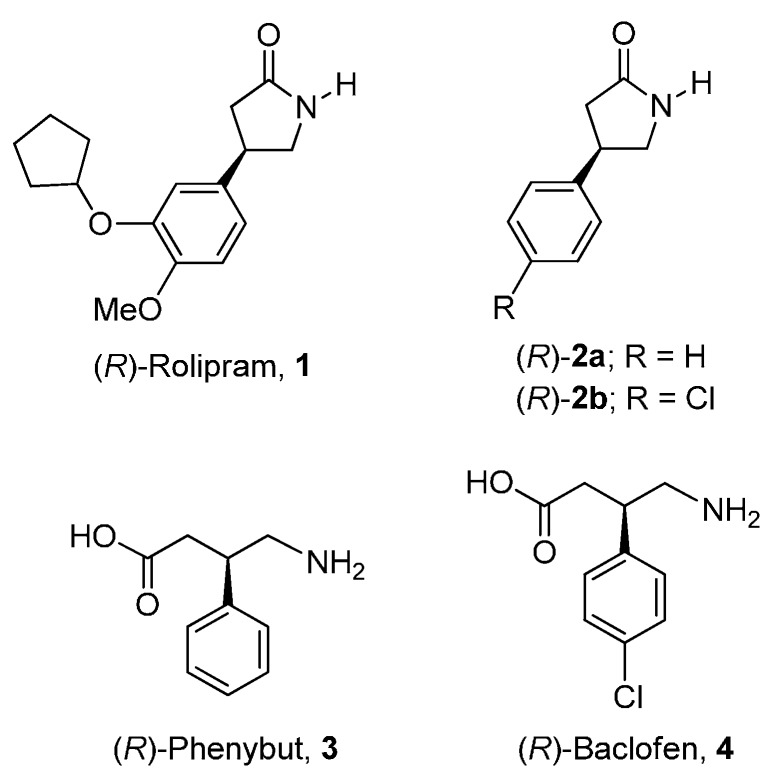
Structures of γ-lactams and GABA derivatives used as pharmaceuticals.

γ-lactams have attracted considerable attention due to their fascinating properties and potential applications in many fields, especially in organic synthesis and medicinal chemistry [1,2,3]. In particular, enantiomerically-pure β-aryl-γ-lactams, such as the (*R*)-Rolipram **1**, considered as a cyclic derivative of GABA, which has shown antipsychotic [4,5,6], antidepressive [7,8], anti-inflammatory, immunosuppressive, and antitumor activity. Additionally, the β-aryl-γ-lactams **2a** and **2b** are precursors for the synthesis of Phenibut **3** and Baclofen **4**, two β-aryl-γ-amino butyric acids (GABA analogues), which are important biological active compounds Figure 1. Phenibut is used as a psychotropic drug, anticonvulsant, antidepressant, and for its anti-neuropathic pain properties [9,10], whereas Baclofen is a GABAB receptor agonist and is marketed for the treatment of multiple neurological disorders, and acts as a muscle relaxant [11]. The biological activity of these compounds depends on its absolute configuration, and the (*R*)-enantiomer is much more active than the (*S*)-enantiomer [12,13,14,15]. Additionally, the β-aryl-γ-lactams are also important key intermediates for the synthesis of more complex compounds [1,16].

Due to the utility of β-aryl-γ-lactams as key synthetic intermediates for the synthesis of γ-amino acids [17] in conjunction with their biological activity, several methods have been reported for the synthesis of γ-lactams [18,19,20,21,22]; however, it is yet highly desirable to develop convenient and milder protocols for its preparation, especially with various substitution patterns and enantiomerically purity. In this paper, we report an efficient synthesis of a series of β-aryl-γ-lactams and its resolution by derivatization with (*S*)-Naproxen. The utility of this methodology was highlighted by the preparation of enantiomerically-enriched (*R*)- and (*S*)-Baclofen hydrochloride.

## 2. Results and Discussion

**Scheme 1:**
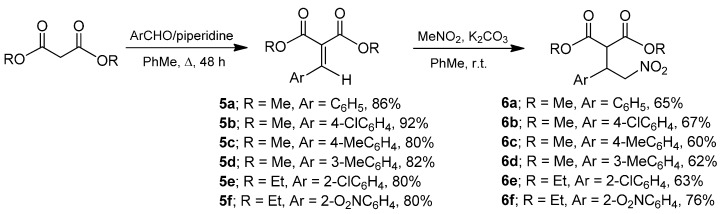
Preparation of nitro derivatives (**6a**–**f**).

For the synthesis of the target β-aryl-γ-lactams (**2a**–**f**), we first carried out the Knoevenagel reaction of diethyl or methyl malonate with different aromatic aldehydes in toluene at reflux in the presence of a catalytic amount of piperidine, leading to the expected arylidenemalonates (**5a**–**f**) in 80% to 92% yield. The reaction proceeds efficiently with electron-rich and electron-withdrawing aromatic substituents. The Michael addition of nitromethane to arylidenemalonates (**5a**–**f**) in the presence of K_2_CO_3_ as a base in toluene at room temperature, furnished the γ-nitro derivatives **6a**–**f** in 60% to 76% yield (Scheme 1) [23,24].

**Scheme 2:**
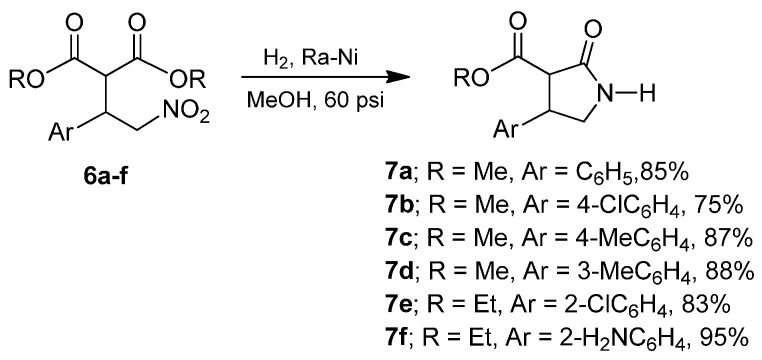
Catalytic reduction of γ-nitroesters **6a**–**f**.

Catalytic hydrogenation of the nitro derivatives (**6a**–**f**) in the presence of catalytic amounts of Raney nickel at 60 psi proceeds efficiently to produce the racemic γ-lactams (**7a**–**f**) in 75% to 95% yield (Scheme 2).

**Figure 2:**
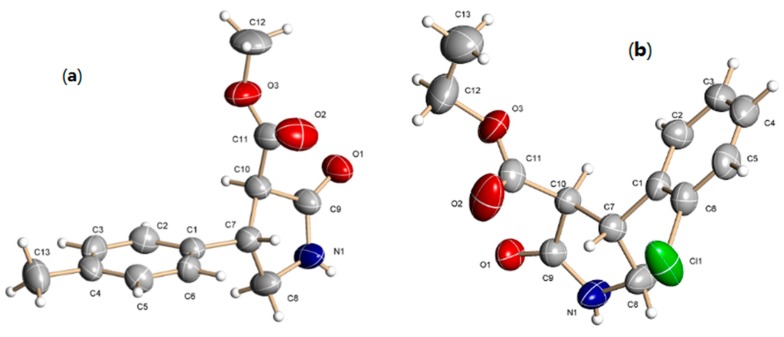
X-ray structures of γ-lactams **7c** (**a**) and **7e** (**b**).

The racemic γ-lactams with *trans*-stereochemistry were obtained as major product, according to the coupling constants (*J* = 10 Hz) for the hydrogens H2 and H3. Additionally, suitable crystals for the γ-lactams **7c** and **7e** were obtained, which were subjected to X-ray analysis (Supplementary Materials) [25], in which it has been confirmed that the orientation of the hydrogens in C7 and C10 are in a *trans* relationship (Figure 2).

**Scheme 3:**
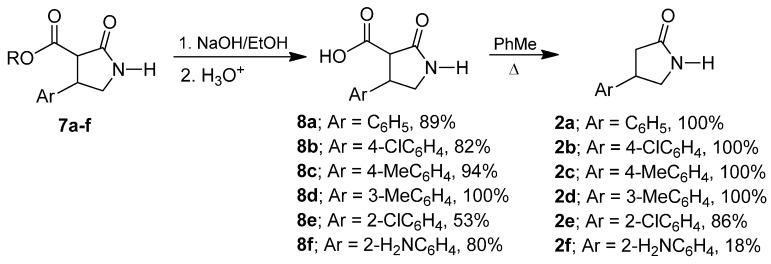
Preparation of racemic γ-lactams (**2a**–**f**).

In the next step we carried out the hydrolysis and decarboxylation of the ester moiety, by treatment of (**7a**–**f**) with 1 N NaOH in ethanol followed by the protonation, obtaining the carboxylic acids derivatives (**8a**–**f**) in 53% to 100% yield which, by heating in toluene, afforded the β-aryl-γ-lactams (**2a**–**f**) in excellent yield (Scheme 3).

**Scheme 4:**
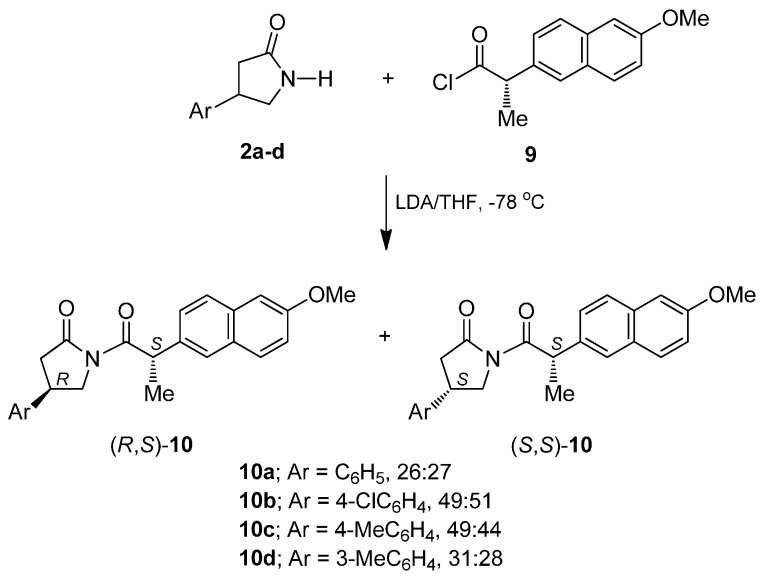
Resolution of γ-lactams **2a**–**d** with (*S*)-Naproxen.

With the racemic β-aryl-γ-lactams (**2a**–**f**) in hand, the next step was to explore the scope of (*S*)-Naproxen as a resolution agent [26,27,28,29,30]. For this purpose, and after several attempts using Et_3_N/DMAP as base, we found that the reaction of the racemic β-phenyl-γ-lactam (**2a**) with lithium diisopropylamide (LDA) in dry tetrahydrofuran at −78 °C, followed by the addition of (*S*)-Naproxen acyl chloride **9** freshly prepared after reaction of (*S*)-Naproxen with oxalyl chloride, produced the imides (*R*,*S*)-**10a** and (*S*,*S*)-**10a** as a diastereoisomeric mixture which, by careful separation by column chromatography, afforded the diastereoisomerically pure imides (*R*,*S*)-**10a** as minor polar and (*S*,*S*)-**10a** as more polar in 26% and 27% yield, respectively. Under identical conditions, the resolution of the β-aryl-γ-lactams (**2c**–**d**) with **9**, afforded the diastereoisomerically pure imides (*R*,*S*)-**10b**–**d** and (*S*,*S*)-**10b**–**d** in good yields (Scheme 4).

**Scheme 5:**
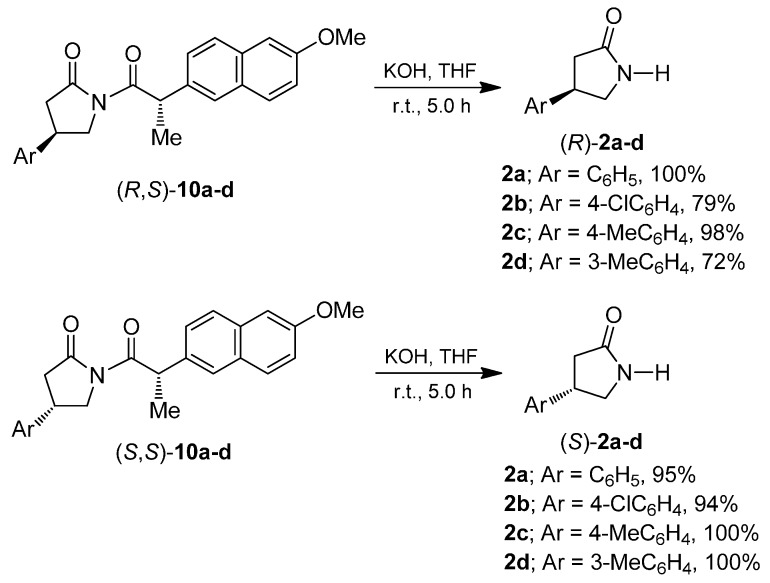
Preparation of enantiomerically-pure β-aryl-γ-lactams (*R*)- and (*S*)-**2a**–**d**.

Subsequently removing the chiral agent in the diastereoisomerically-pure imides (*R*,*S*)-**10a**–**d** and (*S*,*S*)-**10a**–**d** was carried out using 1 N potassium hydroxide in THF to obtain the enantiomerically-pure β-aryl-γ-lactams (*R*)-**2a**–**d** and (*S*)-**2a**–**d** in excellent yield (Scheme 5). The absolute configuration of γ-lactams (*R*)-**2a**–**b** [(*R*)-**2a**: ${\left[\alpha \right]}_{\text{D}}^{20}$ − 19.21; (*R*)-**2b**: ${\left[\alpha \right]}_{\text{D}}^{20}$ − 24.2] and (*S*)-**2a**: ${\left[\alpha \right]}_{\text{D}}^{20}$ + 19.78; (*S*)-**2b**: ${\left[\alpha \right]}_{\text{D}}^{20}$ + 13.53 was assigned by comparing the sign of optical rotation with those reported in the literature [31,32,33,34,35]. The other β-aryl-γ-lactams showed similar characteristics in NMR and the configuration was also assigned by comparing the sign of optical rotation.

**Scheme 6:**
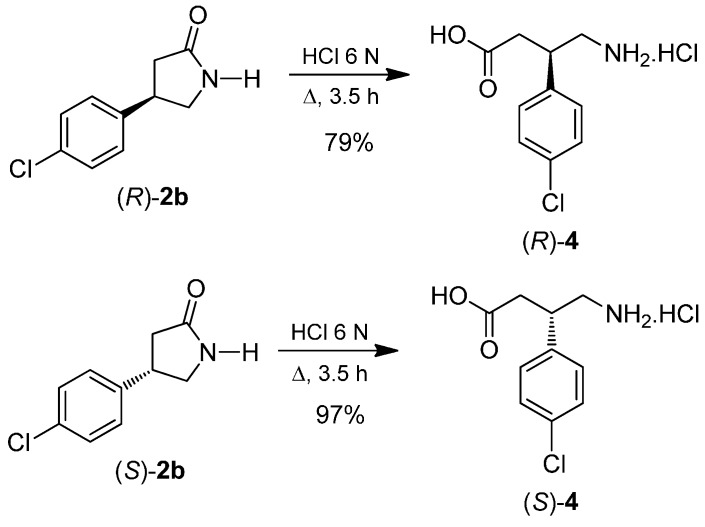
Preparation of (*R*)- and (*S*)-Baclofen hydrochloride **4**.

Finally, the hydrolysis of the β-chlorophenyl-γ-lactam (*R*)-**2b** with 6N HCl, gave the (*R*)-baclofen hydrochloride **4** in 79% yield. Under identical conditions, the β-chlorophenyl-γ-lactam (*S*)-**2b** was transformed into (*S*)-Baclofen hydrochloride **4** in 97% yield (Scheme 6).

## 3. Materials and Methods

### 3.1. General Comments

Reagents were obtained from commercial suppliers and were used without further purification. Melting points were determined in a Fischer Johns apparatus (Pittsburgh, PA, USA) and are uncorrected. NMR spectra were recorded on Varian System instrument (Palo Alto, CA, USA), 400 MHz for ^1^H and 100 MHz for ^13^C) and Varian Gemini 200 MHz, 200 MHz for ^1^H and 50 MHz for ^13^C). The spectra were obtained in CD_3_OD and CDCl_3_ solution using TMS as an internal reference. High-resolution CI^+^ and FAB^+^ mass experiments were made in a JEOL HRMStation JHRMS-700 (Akishima, Tokyo, Japan). X-ray diffraction studies were performed on a Bruker-APEX diffractometer (Madison, WI, USA) with a CCD area detector at 100 K (λ_Mo_ _Kα_ = 0.71073 Å, monochromator:graphite). Specific rotations were measured in a Perkin-Elmer 341 polarimeter (Shelton, CT, USA) at room temperature and λ = 589 nm. The purification of all compounds was carried out by column chromatography using (silica gel 70-230). The dichloromethane was refluxed on phosphorous pentoxide and THF with sodium and benzophenone.

### 3.2. General Procedure for the Preparation of Arylidenemalonates ***5a**–**f***

A mixture of dialkyl malonate (1 eq.), toluene, aryl aldehyde (1 eq.), and 10 drops of piperidine, was refluxed for 48 h. Then, the reaction mixture was acidified to pH = 6–7 by addition of 1M HCl, and the solvent was removed under reduced pressure. The crude product was purified by column chromatography. ^1^H- and ^13^C-NMR data for the compounds **5a**,**d** [36], **5b**,**c** [37], are identical with those described in the literature.

#### 3.2.1. Diethyl 2-(2-Chlorobenzylidene)malonate **5e**

According to the general procedure, diethyl malonate (3.0 g, 18.7 mmol), toluene (35 mL), 2-chlorobenzaldehyde (2.63 g, 18.7 mmol), and piperidine were reacted. The crude product was purified by column chromatography using hexane/AcOEt (9:1) as eluent to afford **5e** (4.2 g, 80%) as a slightly yellow liquid. IR (cm^−1^): 2983, 1724, 1632, 1469, 1248, 1200, 756. ^1^H-NMR (CDCl_3_, 400 MHz,) δ: 1.18 (t, *J* = 7.2 Hz, 3H), 1.34 (t, *J* = 7.2 Hz, 3H), 4.22 (q, *J* = 7.2 Hz, 2H), 4.33 (q, *J* = 7.2 Hz, 2H), 7.21–7.33 (m, 1H), 7.29–7.33 (m, 1H), 7.41–7.45 (m, 2H), 8.02 (s, 1H). ^13^C-NMR (CDCl_3_, 100 MHz) δ: 14.0, 14.3, 61.8, 61.9, 127.0, 129.1, 129.5, 130.0, 131.3, 132.3, 134.9, 139.4, 163.9, 165.9. MS (CI^+^): *m*/*z* 283 (10%), 237 (100%), 219 (12%), 173 (30%). HRMS (CI): calculated for C_14_H_16_ClO_4_ [M+H]^+^, *m*/*z* 283.0737; found for [M + H]^+^, *m*/*z* 283.0712.

#### 3.2.2. Diethyl 2-(2-Nitrobenzyliden)malonate **5f**

According to the general procedure, diethyl malonate (3.0 g, 18.7 mmol), toluene (35 mL), 2-nitrobenzaldehyde (2.82 g, 18.7 mmol), and piperidine were reacted. The crude product was purified by column chromatography on using hexane/AcOEt (8:2) as eluent to afford **5f** (4.4 g, 80%), as a white crystalline solid, m.p.: 64–66 °C. IR (cm^−1^): 2989, 1714, 1626, 1477, 1259, 1201, 757. ^1^H-NMR (CDCl_3_, 400 MHz) δ: 1.32 (t, *J* = 7.2 Hz, 3H), 1.35 (t, *J* = 7.2 Hz, 3H), 4.36 (m, 4H), 7.57–7.61 (m, 1H), 7.75–7.80 (m, 2H), 8.2–8.26 (m, 1H), 8.34 (s, 1H). ^13^C-NMR (CDCl_3_, 100 MHz) δ: 14.1, 14.3, 62.3, 62.4, 123.9, 125.0, 129.6, 130.1, 134.8, 135.3, 139.2, 148.6, 163.6, 165.9. MS (FAB^+^): *m*/*z* 294 (75%), 248 (100%), 232 (13%), 220 (<10%), 202 (<10%), 176 (<10%), 154 (15%), 136 (20%), 107 (<10%), 89 (10%), 77 (<10%). HRMS (FAB): calculated for C_14_H_16_NO_6_ [M + H]^+^, *m*/*z* 294.0978; found for [M + H]^+^, *m*/*z* 294.0959.

### 3.3. General Procedure for the Preparation of Nitroderivatives ***6a**–**f***

To a solution of arylidenemalonates **5a**–**f** in toluene (20 mL) was added nitromethane (5.0 eq.) and potassium carbonate (1.7 eq.). The reaction mixture was stirred at room temperature for 48 h, and then the solvent was evaporated under reduced pressure. The crude product was treated with water (20 mL) and extracted with AcOEt (4 × 25 mL). The organic layer was dried over Na_2_SO_4_, filtered, evaporated, and purified by column chromatography. ^1^H- and ^13^C-NMR data for the compounds **6a**,**b** [38], **6c**,**d** [39], **6e**,**f** [23,40], are identical with those described in the literature.

### 3.4. General Procedure for the Synthesis of the γ-Lactams ***7a**–**f***

A mixture of **6a**–**f** in MeOH (15 mL) and a catalytic amount of Ra-Ni was hydrogenated at room temperature for 2.5 h at 60 psi. The catalyst was filtered off in vacuum through Celite and the filtrate was evaporated under reduced pressure. The crude product was purified by column chromatography or by recrystallization. ^1^H- and ^13^C-NMR data for the compound **7a** was identical with those described in the literature [41].

#### 3.4.1. Ethyl 2-Oxo-4-(4-chlorophenyl)-pyrrolidine-3-carboxylate (±)-**7b**

Following the general procedure, **6b** (0.9 g, 2.8 mmol) was treated with Ra-Ni in MeOH. The crude product was recrystallized (hot EtOH) to give (±)-**7b** as a white solid (0.54 g, 75%), m.p.: 131–133 °C. IR (cm^−1^): 3193, 3094, 2867, 1740, 1701, 1434, 1198, 1161, 821. ^1^H-NMR (CDCl_3_, 400 MHz) δ: 3.40 (ddd, *J* = 10.0, 8.4, 6.0 Hz, 1H), 3.54 (d, *J* = 10 Hz, 1H), 3.78–3.83 (m, 1H), 3.78 (s, 3H), 4.09 (dd, *J* = 18.0, 8.4 Hz, 1H), 7.20 (d, *J* = 8.8, 2H), 7.32 (d, *J* = 8.0, 2H), 7.43 (bs, 1H). ^13^C-NMR (CDCl_3_, 100 MHz) δ: 43.9, 47.8, 53.1, 55.3, 128.6, 129.4, 133.7, 138.3, 169.6, 172.7. ME (FAB^+^): *m*/*z* 254 (60%), 235 (<10%), 222 (<10%), 176 (<10%), 154 (100%), 136 (65%), 107 (18%), 89 (15%), 77 (12%), 65 (<10%), 51 (<10%). HRMS (FAB) calculated for C_12_H_13_ClNO_3_ (M + 1): 254.0584, found 254.0596.

#### 3.4.2. Methyl 2-Oxo-4-(4-methylphenyl)-pyrrolidine-3-carboxylate (±)-**7c**

Following the general procedure, **6c** (1.8 g, 6.09 mmol) was treated with Ra-Ni in MeOH. The crude product was recrystallized (CH_2_Cl_2_/hexane) to give (±)-**7c** as a beige solid (1.2 g, 87%), m.p.: 120–123 °C. IR (cm^−1^): 3186, 3095, 2953, 1742, 1701, 1518, 1158, 1196, 815. ^1^H-NMR (CDCl_3_, 400 MHz) δ: 2.33 (s, 3H), 3.40 (dd, *J* = 17.6, 9.2 Hz, 1H), 3.57 (d, *J* = 9.2 Hz, 1H), 3.77 (s, 3H), 3.77–7.80 (m, 1H), 4.07 (ddd, *J* = 9.2, 9.2, 8.8 Hz, 1H), 7.15 (s, 4 H). ^13^C-NMR (CDCl_3_, 100 MHz) δ: 21.2, 44.2, 48.1, 53.0, 55.5, 127.1, 129.8, 136.9, 137.5, 169.9, 173.0. ME (CI^+^): *m*/*z* 234 (100%), 233 (5%), 202 (30%), 174 (30%). HRMS (CI) calculated for C_13_H_16_NO_3_ (M + 1): 234.1130, found 234.1136.

#### 3.4.3. Methyl 2-Oxo-4-(3-methylphenyl)-pyrrolidine-3-carboxylate (±)-**7d**

According to the general procedure, **6d** (0.8 g, 2.7 mmol) was hydrogenated in the presence of a catalytic amount of Ra-Ni in MeOH (15 mL). The crude product was recrystallized from hot EtOH, to give (±)-**7d** (0.56 g, 88 %) as a white solid, m.p.: 105–109 °C. IR (cm^−1^): 3208, 2948, 1739, 1694, 1433, 1167, 786, 775, 701. ^1^H-NMR (CDCl_3_, 400 MHz) δ: 2.34 (s, 3H), 3.40–3.44 (m, 1H), 3.60 (d, *J* = 9.6 Hz, 3H), 3.78 (dd, *J* = 17.6, 9.2 Hz, 1H), 3.79 (s, 3H), 4.07 (dd, *J* = 17.6, 8.4 Hz, 1H), 7.04–7.10 (m, 2H), 7.21–7.26 (m, 2H), 7.34 (bs, 1H). ^13^C-NMR (CDCl_3_, 100 MHz) δ: 21.6, 44.4, 48.1, 53.0, 55.4, 124.2, 127.9, 128.5, 129.1, 138.9, 139.9, 169.8, 173.0. MS (FAB^+^): *m*/*z* HRMS 234 (100%), 202 (25%), 174 (15%), 159 (<10%), 137 (<10%), 91 (<10%), 77 (<10%). (FAB): calculated for C_13_H_16_NO_3_ [M + H]^+^, *m*/*z* 234.1130; found for [M + H]^+^, *m*/*z* 234.1135.

#### 3.4.4. Ethyl 2-Oxo-4-(2-chlorophenyl)-pyrrolidine-3-carboxylate (±)-**7e**

According to the general procedure, **6e** (1.2 g, 3.5 mmol) was hydrogenated in the presence of a catalytic amount of Ra-Ni in MeOH (18 mL). The crude product was recrystallized from hexane/CH_2_Cl_2_ mixture, to give (±)-**7e** (0.77 g, 83%) as a white solid, m.p.: 108–110 °C. IR (cm^−1^): 3207, 3100, 2868, 1732, 1698, 1481, 1175, 1152, 757. ^1^H-NMR (CDCl_3_, 400 MHz) δ: 1.28 (t, *J* = 7.2 Hz, 3H), 3.40 (dd, *J* = 9.6, 7.6 Hz, 1H), 3.66 (d, *J* = 7.6 Hz, 1H), 3.92 (dd, *J* = 9.6, 8.8 Hz, 1H), 4.23 (q, *J* = 7.2 Hz, 2H), 4.52 (ddd, *J* = 8.8, 7.6, 7.6 Hz, 1H), 7.21–7.33 (m, 3H), 7.39–7.41 (d, *J* = 7.6 Hz, 1H), 7.51 (bs, 1H). ^13^C-NMR (CDCl_3_, 100 MHz) δ: 14.2, 41.4, 47.1, 54.4, 62.1, 127.6, 128.0, 129.0, 130.4, 134.1, 169.2, 173.0. MS (CI^+^): *m*/*z* 268 (100%), 267 (5%), 222 (45%), 194 (20%). HRMS (CI) calculated for C_13_H_15_ClNO_3_ [M + H]^+^, *m*/*z* 268.0740; found for [M + H]^+^, *m*/*z* 268.0745.

#### 3.4.5. Ethyl 2-Oxo-4-(2-aminophenyl)-pyrrolidine-3-carboxylate (±)-**7f**

According to the general procedure, **6f** (0.7 g, 1.9 mmol) was hydrogenated in the presence of a catalytic amount of Ra-Ni in MeOH (15 mL). The crude product was recrystallized from Et_2_O/CH_2_Cl_2_/hexane to give the *trans-***7f** (0.40 g, 83%) as a white solid, m.p.: 102–105 °C. IR (cm^−1^): 3458, 3352, 3210, 3105, 2956, 1721, 1701, 1470, 1178, 787. ^1^H-NMR (CDCl_3_, 400 MHz) δ: 1.27 (t, *J* = 7.2 Hz, 3H), 3.39 (m, 1H), 3.54 (d, *J* = 9.6 Hz, 3H), 3.76 (m, 1H), 3.98 (dd, *J* = 17.6, 8.4 Hz, 1H), 4.22 (q, *J* = 7.2 Hz, 2H), 6.59 (m, 3H), 7.11 (m, 1H), 7.24 (bs, 1H).^13^C-NMR (CDCl_3_, 100 MHz) δ: 14.3, 44.5, 47.9, 55.4, 62.0, 113.7, 114.4, 117.1, 130.1, 141.3, 147.2, 169.6, 173.1. MS (FAB^+^): *m*/*z* 249 (75%), 248 (27%), 203 (28%), 175 (40%), 149 (100%), 132 (<10%), 113 (15%), 71 (35%), 57 (47%). HRMS (FAB) calculated for C_13_H_17_N_2_O_3_ [M + H]^+^, *m*/*z* 249.1239; found for [M + H]^+^, *m*/*z* 249.1251.

### 3.5. General Procedure for The Preparation of the Carboxylic Acids ***8a**–**f***

To a suspension of **7a**–**f** in ethanol (2 mL) was added 1 N NaOH (0.8 mL) was stirred at room temperature for 48 h. The ethanol was removed at reduced pressure and the residue was acidified with 1M HCl. The precipitate formed was filtered under vacuum.

#### 3.5.1. 2-Oxo-4-phenyl-pyrrolidine-3-carboxylic Acid **8a**

According to the general procedure, **7a** (0.38 g, 1.7 mmol) was treated with 1 N NaOH (1.5 mL) to give **8a** (0.31 g, 89%) as a white solid, m.p.: 158–162 °C. IR (cm^−1^): 3283, 1686, 1489, 766, 703. ^1^H**-**NMR (MeOD, 400 MHz) δ: 3.39 (dd, *J* = 9.2, 8.8 Hz, 1H), 3.58 (d, *J* = 10.0 Hz, 1H), 3.75 (dd, *J* = 9.2, 8.8 Hz, 1H), 4.01 (ddd, *J* = 10.0, 8.8, 8.8 Hz, 1H), 7.24–7.35 (m, 5H). ^13^C-NMR (MeOD, 100 MHz) δ: 46.4, 48.9, 56.9, 128.2, 128.6, 130.1, 141.6, 172.8, 175.1. MS (FAB^+^): *m*/*z* 206 (100%), 188 (20%), 154 (55%), 136 (38%), 107 (11%), 77 (14%). HRMS (FAB) calculated for C_11_H_12_NO_3_ [M + H]^+^, *m*/*z*; 206.0817, found for [M + H]^+^, *m*/*z* 206.0815.

#### 3.5.2. 2-Oxo-4-(4-chlorophenyl)-pyrrolidine-3-carboxylic Acid **8b**

According to the general procedure, **7b** (0.2 g, 0.79 mmol) was treated with 1 N NaOH (0.8 mL) to give **8b** (0.15 g, 82%) as a beige solid, m.p.: 140–142 °C. IR (cm^−1^): 2883, 1739, 1670, 1486, 822. ^1^H-NMR (MeOD, 400 MHz) δ: 3.37 (t, *J* = 9.6 Hz, 1H), 3.57 (d, *J* = 9.6 Hz, 1H), 3.75 (dd, *J* = 9.6, 8.8 Hz, 1H), 4.01 (t, *J* = 8.8 Hz, 1H), 7.32 (s, 4H). ^13^C-NMR (MeOD, 100 MHz) δ: 45.9, 48.7, 56.8, 130.0, 130.1, 134.4, 140.3, 172.7, 175.0. MS (FAB^+^): *m*/*z* 240 (98%), 222 (20%), 176 (10%), 154 (100%), 137 (70%), 107 (23%), 89 (22%), 77 (20%), 65 (<10%), 51 (<10%). HRMS calculated for C_11_H_11_ClNO_3_ [M + H]^+^, *m*/*z* 240.0427; found for [M + H]^+^, *m*/*z* 240.0431.

#### 3.5.3. 2-Oxo-4-(4-methylphenyl)-pyrrolidine-3-carboxylic Acid **8c**

According to the general procedure, **7c** (0.2 g, 0.85 mmol) was treated with 1 N NaOH (0.8 mL) to give **8c** (0.17 g, 94%) as a white solid, m.p.: 166–169 °C. IR (cm^−1^): 3255, 2964, 1738, 1667, 1488, 808. ^1^H-NMR (MeOD, 400 MHz) δ: 2.30 (s, 3H), 3.30–3.31 (m, 1H), 3.36 (t, *J* = 9.6 Hz, 1H), 3.57 (d, *J* = 9.6 Hz, 1H), 3.72 (t, *J* = 9.6 Hz, 1H), 3.93–4.0 (m, 1H), 7.12–7.20 (m, 4H). ^13^C-NMR (MeOD, 100 MHz) δ: 21.2, 46.1, 49.0, 56.0, 128.0, 128.8, 130.1, 130.5, 138.3, 172.8, 175.1. MS (CI^+^): *m*/*z* 219 (<10%), 202 (18%), 175 (100%), 145 (10%), 118 (85%), 91 (<10%). HRMS (CI^+^) calculated for C_12_H_14_NO_3_ [M + H]^+^, *m*/*z* 220.0974; found for [M + H]^+^, *m*/*z* 220.0976.

#### 3.5.4. 2-Oxo-4-(3-methylphenyl)-pyrrolidine-3-carboxylic Acid **8d**

According to the general procedure, **7d** (0.2 g, 0.85 mmol) was treated with 1 N NaOH (0.8 mL) to give **8d** (0.18 g, 100%) as a white solid, m.p.: 173–177 °C. IR (cm^−1^): 3332, 2891, 1731, 1666, 1427, 731, 686, 642. ^1^H-NMR (MeOD, 400 MHz) δ: 2.32 (s, 3H), 3.38 (dd, *J* = 10.0, 8.8 Hz, 1H), 3.56 (d, *J* = 10.0 Hz, 1H), 3.73 (dd, *J* = 10.0, 8.8 Hz, 1H), 3.97 (ddd, *J* = 10.0, 8.8, 8.8 Hz, 1H), 7.07–7.14 (m, 3H), 7.20–7.23 (m, 1H). ^13^C-NMR (MeOD, 50 MHz) δ: 21.6, 46.6, 48.9, 57.0, 125.3, 128.9, 129.3, 130.0, 141.5, 172.9, 175.3. MS (FAB^+^): *m*/*z* 220 (100%), 202 (37%), 154 (65%), 136 (55%), 89 (25%), 77 (23%), 57 (12%). HRMS (FAB) calculated for C_12_H_14_NO_3_ [M + H]^+^, *m*/*z* 220.0974; found for [M + H]^+^, *m*/*z* 220.0966.

#### 3.5.5. 2-Oxo-4-(2-chlorophenyl)-pyrrolidine-3-carboxylic Acid **8e**

According to the general procedure, **7e** (0.2 g, 0.7 mmol) was treated with 1 N NaOH (0.8 mL) to give **8e** (0.89 g, 53%) as a white solid, m.p.: 154–156 °C. IR (cm^−1^): 3290, 2887, 1751, 1656, 1478, 760. ^1^H-NMR (MeOD, 400 MHz) δ: 3.61 (dd, *J* = 10.0, 7.6 Hz, 1H), 3.68 (d, *J* = 8.4 Hz, 1H), 3.85 (dd, *J* = 10.0, 8.4 Hz, 1H), 4.44–4.50 (m, 1H), 7.24–7.29 (m, 1H), 7.31–7.35 (m, 1H), 7.41–7.47 (m, 2H). ^13^C-NMR (MeOD, 50 MHz) δ: 43.1, 48.0, 55.6, 128.9, 129.3, 130.1, 131.2, 135.1, 138.9, 172.6, 174.9. MS (FAB^+^): *m*/*z* 240 (85%), 222 (25%), 154 (100%), 136 (80%), 107 (25%), 89 (26%), 77 (23%). HRMS calculated for C_11_H_11_ClNO_3_ [M + H]^+^, *m*/*z* 240.0427; found for [M + H]^+^, *m*/*z* 240.0429.

#### 3.5.6. 2-Oxo-4-(2-aminophenyl)-pyrrolidine-3-carboxylic Acid **8f**

According to general procedure, **7f** (0.2 g, 0.8 mmol) was treated with 1 N NaOH (0.8 mL) to give **8f** (0.14 g, 80%) as a brown liquid. IR (cm^−1^): 3366, 2883, 1674, 1493, 793. ^1^H-NMR (MeOD, 400 MHz) δ: 3.44 (dd, *J* = 9.6, 8.8 Hz, 1H), 3.63 (d, *J* = 10 Hz, 1H), 3.81 (dd, *J* = 9.6, 9.2 Hz, 1H), 4.08–4.12 (m, 1H), 7.34 (d, *J* = 7.2, 1H), 7.41 (s, 1H), 7.48–7.56 (m, 2H). ^13^C-NMR (MeOD, 100 MHz) δ: 45.9, 47.0, 56.6, 123.1, 123.2, 129.1, 132.0, 133.0, 144.3, 172.4, 174.6. MS (FAB^+^) *m*/*z* 220 (<10%), 203 (<10%), 176 (10%), 154 (100%), 136 (85%), 120 (12%) 107 (18%), 89 (<10%). HRMS (FAB) calculated for C_11_H_13_N_2_O_3_ [M + H]^+^, *m*/*z* 221.0926; found for [M + H]^+^, *m*/*z* 221.0976.

### 3.6. General Procedure of the Synthesis of β-Aryl-γ-lactams (±)-***2a**–**f***

A suspension of carboxylic acid in toluene was heated to reflux for 5 h. After cooling to room temperature, the solvent was evaporated and the pure product was obtained. ^1^H- and ^13^C-NMR data for the compounds (±)-**2a**–**c** [42], are identical with those described in the literature.

#### 3.6.1. 4-(3-Methylphenyl)-pyrrolidin-2-One (±)-**2d**

According to the general procedure **8d** (0.15 g, 0.7 mmol) was refluxed to give (±)-**2d** (0.12 g, 100%) as a beige solid, m.p.: 103–104 °C. IR (cm^−1^): 3211, 3095, 2892, 1677, 1489, 790, 706, 684. ^1^H-NMR (CDCl_3_, 200 MHz) δ: 2.35 (s, 3H), 2.49 (dd, *J* = 16.8, 8.6 Hz, 1H), 2.72 (dd, *J* = 16.8, 8.6 Hz, 1H), 3.41 (dd, *J* = 16.8, 8.6 Hz, 1H), 3.57–3.68 (m, 1H), 3.77 (dd, *J* = 16.8, 8.6 Hz, 1H), 7.03–7.08 (m, 2H), 7.19–7.27 (m, 2H). ^13^C-NMR (CDCl_3_, 50 MHz) δ: 36.9, 37.3, 48.5, 127.2, 127.5, 128.4, 130.0, 133.9, 139.6, 178.0. MS (FAB^+^) *m*/*z* 176 (100%), 131 (<10%), 105 (10%), 91 (11%), 69 (12%), 55 (14%), 43 (12%). HRMS (FAB) calculated for C_11_H_14_NO [M + H]^+^, *m*/*z* 176.1075; found for [M + H]^+^, *m*/*z* 176.1077.

#### 3.6.2. 4-(2-Chlorophenyl)-pyrrolidin-2-One (±)-**2e**

According to general procedure, **8e** (0.11 g, 0.46 mmol) was refluxed to give (±)-**2e** (80 mg, 86%) as a beige solid, m.p.: 112–115 °C. IR (cm^−1^): 3174, 3080, 2884, 1686, 1486, 746. ^1^H-NMR (CDCl_3_, 200 MHz) δ: 2.48 (dd, *J* = 17.2, 7.4 Hz, 1H), 2.74 (dd, *J* = 17.2, 8.8 Hz, 1H), 3.41 (dd, *J* = 9.8, 6.2 Hz, 1H), 3.85 (dd, *J* = 9.8, 8.4 Hz, 1H), 4.18–7.25 (m, 1H), 7.25–7.29 (m, 1H), 7.33–7.34 (d, *J* = 7.2 Hz, 1H), 7.38–7.40 (d, *J* = 7.6 Hz, 2H). ^13^C-NMR (CDCl_3_, 50 MHz) δ: 36.9, 37.3, 48.5, 127.2, 127.5, 128.4, 130.0, 133.9, 139.6, 178.0. MS (FAB^+^) *m*/*z* 196 (100%), 162 (<10%), 154 (10%), 137 (11%), 107 (<10%), 77 (<10%), 55 (<10%), 41 (<10%). HRMS (FAB) calculated for C_10_H_11_ClNO [M + H]^+^, *m*/*z* 196.0529; found for [M + H]^+^, *m*/*z* 196.0541.

#### 3.6.3. 4-(2-Aminophenyl)-pyrrolidin-2-one (±)-**2f**

According to general procedure, **8f** (0.2 g, 0.7 mmol) was refluxed in toluene for five hours to give (±)-**2f** (60 mg, 18%) as a beige solid, m.p.: 122–125 °C. IR (cm^−1^): 3422, 3347, 3243, 2923, 1668, 792. ^1^H-NMR (CDCl_3_, 200 MHz) δ: 2.47 (dd, *J* = 17.0, 8.6 Hz, 1H), 2.69 (dd, *J* = 17.0, 8.6 Hz, 1H), 3.57 (dddd, *J* = 8.6, 8.2, 8.2, 7.6 Hz, 1H), 3.73 (dd, *J* = 9.0, 8.2 Hz, 2H), 6.55–6.63 (m, 2H), 7.06–7.14 (m, 2H). ^13^C-NMR (CDCl_3_, 50 MHz) δ: 38.0, 40.3, 49.6, 113.4, 114.0, 117.0, 129.8, 143.6, 147.0, 178.4. MS (CI^+^): *m*/*z* 177 (100%), 176 (78%) 160 (11%), 119 (45%). HRMS (CI) calculated for C_10_H_13_N_2_O [M + H]^+^, *m*/*z* 177.1028; found for [M + H]^+^, *m*/*z* 177.1025.

### 3.7. Synthesis of (S)-Naproxen Acyl Chloride ***9***

To a solution of (*S*)-Naproxen (2.5 eq.) in anhydrous CH_2_Cl_2_ (15 mL) and *N*,*N*-dimethyl formamide (one drop), oxalyl chloride (3 eq.) at 0 °C was added. The reaction mixture was stirred at room temperature for 2.5 h under a nitrogen atmosphere, and after this time, the solvent and residual oxalyl chloride were removed under reduced pressure to continue the reaction, obtaining the (*S*)-Naproxen acyl chloride **9**, which was not isolated and used immediately in the next reaction.

### 3.8. General Procedures for The Resolution of β-Aryl-γ-lactams (±)-***2a–d***

A solution of **8a**–**d** (1 eq.) in anhydrous tetrahydrofuran (10 mL) was added dropwise to a freshly prepared LDA (1.1 eq.) at −78 °C. The reaction mixture was stirred for 30 min at room temperature under a nitrogen atmosphere. Then, the mixture was cooled to −78 °C followed by the addition of crude (*S*)-**9**. The reaction mixture was allowed to room temperature and stirred for 2 h under a nitrogen atmosphere. After, a saturated solution of ammonium chloride was added and extracted with dichloromethane (3 × 15 mL). Finally the solvent was removed under reduced pressure and purified by column chromatography, to obtain the diastereoisomeric pure (*R*,*S*)- and (*S*,*S*)-imides **10a**–**d**.

#### 3.8.1. (*R*)-1-((*S*)-2(6-Methoxynaphth-2-yl)propionyl)-4-phenyl-pyrrolidin-2-one (*R,S*)-**10a** and (*S*)-1-((*S*)-2(6-methoxynaphth-2-yl)propionyl)-4-phenyl-pyrrolidin-2-one (*S*,*S*)-**10a**

According to the general procedure, the reaction of **8a** (50 mg, 0.31 mmol) with LDA (39 mg, 0.37 mmol) and (*S*)-**9** (190 mg, 0.77 mmol), followed by purification in column chromatography using hexane/AcOEt (90:10), afforded the diastereoisomers (*R*,*S*)-**10a** (30 mg, 26%) and (*S*,*S*)-**10a** (31 mg, 27%), both as an amber liquid.

(*R,S*)-**10a**: ${\left[\alpha \right]}_{\text{D}}^{20}$ + 87.13 (*c* 0.95, CHCl_3_). IR (cm^−1^): 2933, 1734, 1685, 1604, 1482, 1190, 760, 698. ^1^H-NMR (CDCl_3_, 400 MHz) δ: 1.57 (d, *J* = 7.2 Hz, 3H), 2.76 (dd, *J* = 17.6, 10.4 Hz, 1H), 2.84 (dd, *J* = 17.6, 8.4 Hz, 1H), 3.38 (dddd, *J* = 10.4, 8.8, 8.4, 8.4 Hz, 1H), 3.79 (dd, *J* = 12.0, 8.8 Hz, 1H), 3.91 (s, 3H), 4.19 (dd, *J* = 12.0, 8.4 Hz, 1H), 5.25 (q, *J* = 7.2 Hz, 1H), 7.11–7.20 (m, 4H), 7.25–7.29 (m, 1H), 7.29–7.36 (m, 2H), 7.48–7.51 (m 1H), 7.70–7.75 (m, 3H). ^13^C-NMR (CDCl_3_, 100 MHz) δ: 19.4, 35.8, 41.1, 44.7, 52.5, 55.3, 105.6, 118.9, 126.6, 126.7, 127.0, 127.1, 127.4, 129.0, 129.4, 133.7, 136.2, 140.3, 157.6, 173.4, 175.4. MS (CI^+^): *m*/*z* 374 (100%), 373 (38%) 212 (70%), 185 (23%), 162 (18%). HRMS (CI) calculated for C_24_H_24_NO_3_ [M + H]^+^, *m*/*z* 374.1756; found for [M + H]^+^, *m*/*z* 374.1772.

(*S,S*)-**10a**: ${\left[\alpha \right]}_{\text{D}}^{20}$ + 83.53 (*c* 0.82, CHCl_3_). IR (cm^−1^): 2933, 1734, 1686, 1604, 1482, 1189, 760, 698. ^1^H-NMR (CDCl_3_, 400 MHz) δ: 1.55 (d, *J* = 6.8 Hz, 3H), 2.57 (dd, *J* = 17.6, 8.0 Hz, 1H), 2.91 (dd, *J* = 17.6, 8.8 Hz, 1H), 3.45–3.49 (m, 1H), 3.67 (dd, *J* = 11.6, 6.8 Hz, 1H), 3.90 (s, 3H), 4.30 (dd, *J* = 11.6, 8.0 Hz, 1H), 5.22 (q, *J* = 6.8 Hz, 1H), 6.91–6.93 (m, 2H), 7.05–7.14 (m, 5H), 7.45–7.46 (m, 1H), 7.67–7.68 (m, 3H). ^13^C-NMR (CDCl_3_, 100 MHz) δ: 19.5, 36.2, 41.4, 44.9, 52.8, 55.5, 105.8, 119.0, 126.5, 126.6, 127.2, 127.4, 127.4, 129.0, 129.1, 129.6, 133.9, 136.2, 141.2, 157.8, 173.8, 175.6. MS (CI^+^): *m*/*z* 374 (70%), 373 (55%) 212 (100%), 185 (32%), 184 (19%), 162 (18%), 141 (<10%). HRMS (CI) calculated for C_24_H_24_NO_3_ [M + H]^+^, *m*/*z* 374.1756; found for [M + H]^+^, *m*/*z* 374.1772.

#### 3.8.2. (*R*)-4-(4-Chlorophenyl)-1-((*S*)-2-(6-methoxynaphth-2-yl)propionyl)-pyrrolidin-2-one (*R*,*S*)-**10b** and (S)-4-(4-Chlorophenyl)-1-((*S*)-2-(6-methoxynaphth-2-yl)propionyl)-pyrrolidin-2-one (*S*,*S*)-**10b**

According to the general procedure, **8b** (86 mg, 0.4 mmol) with LDA (56 mg, 0.52 mmol) and (*S*)-**9** (27 mg, 1.1 mmol), followed by purification in column chromatography using hexane/AcOEt (85:15), afforded the diastereoisomers (*R*,*S*)-**10b** (86 mg, 49%) and (*S*,*S*)-**10b** (92 mg, 51%), both as an colorless liquid.

(*R,S*)-**10b**: ${\left[\alpha \right]}_{\text{D}}^{20}$ + 44.48 (*c* 1.0, CHCl_3_). IR (cm^−1^): 2929, 1735, 1686, 810. ^1^H-NMR (CDCl_3_, 400 MHz) δ: 1.54 (d, *J* = 7.2 Hz, 3H), 2.69 (dd, *J* = 17.2, 10.0 Hz, 1H), 2.82 (dd, *J* = 17.2, 8.4 Hz, 1H), 3.35 (dddd, *J* = 10.0, 8.8, 8.4, 8.4 Hz, 1H), 3.74 (dd, *J* = 12.0, 8.8 Hz, 1H), 3.90 (s, 3H), 4.17 (dd, *J* = 12.0, 8.4 Hz, 1H) 5.21 (q, *J* = 7.2 Hz, 1H), 7.10–7.14 (m, 4H), 7.29–7.31 (m, 2H), 7.45–7.48 (m, 1H), 7.68–7.71 (m, 3H). ^13^C-NMR (CDCl_3_, 100 MHz) δ: 19.5, 35.5, 41.2, 44.9, 52.5, 55.5, 105.7, 119.1, 126.8, 127.2, 127.3, 128.2, 129.1 129.3, 129.5, 133.4, 133.8, 136.2, 138.9, 157.8, 173.2, 175.6. MS (FAB^+^): *m*/*z* 408 (25%), 185 (100%), 136 (24%), 95 (28%), 69 (55%), 55 (30%). HRMS (FAB) calculated for C_24_H_23_ClNO_3_ [M + H]^+^, *m*/*z* 408.1366; found for [M + H]^+^, *m*/*z* 408.1383.

(*S,S*)-**10b**: ${\left[\alpha \right]}_{\text{D}}^{20}$ + 39.08 (*c* 0.97, CHCl_3_). IR (cm^−1^): 2932, 1735, 1688, 811. ^1^H-NMR (CDCl_3_, 200 MHz) δ: 1.55 (d, *J* = 7.2 Hz, 3H), 2.50 (dd, *J* = 17.2, 7.2 Hz, 1H), 2.90 (dd, *J* = 17.2, 8.4 Hz, 1H), 3.42 (m, 1H), 3.62 (dd, *J* = 11.6, 6.0 Hz, 1H), 3.92 (s, 3H), 4.25 (dd, *J* = 11.6, 8.0 Hz, 1H), 5.20 (q, 7.2 Hz, 1H), 6.76–6.78 (m, 2H), 6.92–6.95 (m, 2H), 7.12–7.15 (m, 2H), 7.41–7.44 (m, 1H), 7.66–7.69 (m, 3H). ^13^C-NMR (CDCl_3_, 100 MHz) δ: 19.3, 35.6, 41.2, 45.0, 52.7, 55.5, 105.7, 119.1, 126.6, 127.2, 127.8, 129.1, 129.6, 133.1, 133.9, 136.0, 139.9, 157.9, 173.4, 175.6. MS (FAB^+^): *m*/*z* 408 (12%), 407 (<10%), 307 (27%), 289 (13%), 212 (18%), 185 (12%), 154 (100%), 136 (66%), 107 (18%), 77 (13%). HRMS (FAB) calculated for C_24_H_23_ClNO_3_ [M + H]^+^, *m*/*z* 408.1366; found for [M + H]^+^, *m*/*z* 408.1383.

#### 3.8.3. (*R*)-1-((*S*)-2-(6-Methoxynaphth-2-yl)propionyl-4-(4-methylphenyl)-pyrrolidin-2-one (*R*,*S*)-**10c** and (*S*)-1-((*S*)-2-(6-Methoxynaphth-2-yl)propionyl-4-(4-methylphenyl)-pyrrolidin-2-one (*S*,*S*)-**10c**, (*R*,*S*)-**10c** and (*S,S*)-**10c**

According to the general procedure, the reaction of **8c** (0.2 g, 1.14 mmol) with LDA (0.14 g, 1.37 mmol) and (*S*)-**9** (0.709 g, 2.85 mmol), followed by purification in column chromatography using hexane/AcOEt (90:10), afforded the diastereoisomers (*R*,*S*)-**10c** (0.21 g, 49%) as a colorless liquid, and (*S*,*S*)-**10c** (0.19 g, 44%) as a beige solid, m.p.: 61–64 °C.

(*R,S*)-**10c**: ${\left[\alpha \right]}_{\text{D}}^{20}$ + 92.96 (*c* 0.88, CHCl_3_). IR (cm^−1^): 2930, 1735, 1686, 1604, 1481, 1189, 812. ^1^H-NMR (CDCl_3_, 400 MHz) δ: 1.54 (d, *J* = 6.8 Hz, 3H), 2.33 (s, 3H), 2.73 (dd, *J* = 17.6, 10.4 Hz, 1H), 2.82 (dd, *J* = 17.6, 8.4 Hz, 1H), 3.36 (dddd, *J* = 10.4, 9.2, 8.4, 8.0 Hz, 1H), 3.76 (dd, *J* = 11.6, 9.2 Hz, 1H), 3.91 (s, 3H), 4.17 (dd, *J* = 11.6, 8.0 Hz, 1H), 5.22 (q, *J* = 6.8 Hz, 1H), 7.07–7.15 (m, 6H), 7.46–7.49 (m, 1H), 7.68–7.72 (m, 3H). ^13^C-NMR (CDCl_3_, 100 MHz) δ: 19.5, 21.1, 35.7, 41.4, 44.8, 52.8, 55.5, 105.7, 118.9, 126.7, 126.7, 127.2, 129.1, 129.5, 129.8, 133.8, 136.3, 137.3, 137.2, 137.4, 157.8, 173.7, 175.6. MS (CI^+^): *m*/*z* 388 (100%), 387 (55%) 212 (90%), 185 (20%), 176 (15%). HRMS (CI) calculated for C_25_H_26_NO_3_ [M + H]^+^, *m*/*z* 388.1913; found for [M + H]^+^, *m*/*z* 388.1905.

(*S,S*)-**10c**. ${\left[\alpha \right]}_{\text{D}}^{20}$ + 98.96 (*c* 1.1, CHCl_3_). IR (cm^−1^): 2931, 1735, 1686, 1604, 1482, 1185, 813. ^1^H-NMR (CDCl_3_, 400 MHz) δ: 1.55 (d, *J* = 6.8 Hz, 3H), 2.23 (s, 3H), 2.57 (dd, *J* = 17.6, 8.4 Hz, 1H), 2.90 (dd, *J* = 17.6, 8.4 Hz, 1H), 3.41–3.49 (m, 1H), 3.64 (dd, *J* = 12.0, 7.6 Hz, 1H), 3.91 (s, 3H), 4.29 (dd, *J* = 12.0, 7.6 Hz, 1H), 5.21 (q, *J* = 6.8 Hz, 1H), 6.81–6.88 (m, 4H), 7.11–7.14 (m, 2H), 7.44–7.47 (m, 1H), 7.67–7.70 (m, 3H). ^13^C-NMR (CDCl_3_, 100 MHz) δ: 19.5, 21.1, 35.9, 41.5, 44.9, 52.9, 55.5, 105.7, 118.9, 126.4, 126.6, 127.2, 127.4, 133.9, 136.2, 137.0, 138.1, 157.9, 173.9, 175.6. MS (CI^+^): *m*/*z* 388 (88%), 387 (48%) 212 (100%), 185 (23%), 176 (12%). HRMS (CI) calculated for C_25_H_26_NO_3_ [M + H]^+^, *m*/*z* 388.1913, found for [M + H]^+^, *m*/*z* 388.1904.

#### 3.8.4. (*R*)-1-((*S*)-2-(6-Methoxynaphth-2-yl)propionyl)-4-(3-methylphenyl)-pyrrolidin-2-one (*R*,*S*)-**10d** and (*S*)-1-((*S*)-2-(6-Methoxynaphth-2-yl)propionyl)-4-(3-methylphenyl)-pyrrolidin-2-one (*S*,*S*)-**10d**

According to the general procedure, the reaction of **8d** (50 mg, 0.28 mmol) with LDA (36 mg, 0.34 mmol) and (*S*)-**9** (0.17 g, 0.7 mmol), followed by purification in column chromatography using hexane/AcOEt (90:10), afforded the diastereoisomers (*R*,*S*)-**10d** (33 mg, 31%) and (*S*,*S*)-**10d** (30 mg, 28%), both as an amber liquid.

(*R,S*)-**10d**: ${\left[\alpha \right]}_{\text{D}}^{20}$ + 87.14 (*c* 0.95, CHCl_3_). IR (cm^−1^): 2931, 1735, 1686, 1604, 1482, 1198, 727, 701, 672. ^1^H-NMR (CDCl_3_, 400 MHz) δ: 1.55 (d, *J* = 7.2 Hz, 3H), 2.33 (s, 3H), 2.75 (dd, *J* = 17.6, 10.4 Hz, 1H), 2.83 (dd, *J* = 17.6, 8.4 Hz, 1H), 3.35 (dddd, *J* = 10.4, 9.2, 8.4, 8.4 Hz, 1H), 3.78 (dd, *J* = 12.0, 9.2 Hz, 1H), 3.90 (s, 3H), 4.17 (dd, *J* = 12.0, 8.4 Hz, 1H), 5.23 (q, *J* = 7.2 Hz, 1H), 6.98–7.0 (m, 2H), 7.07–7.14 (m, 3H), 7.20–7.25 (m, 1H), 7.46–7.49 (m, 1H), 7.68–7.73 (m, 3H). ^13^C-NMR (CDCl_3_, 100 MHz) δ: 19.3, 21.4, 35.7, 41.2, 44.7, 52.5, 55.3, 105.5, 118.9, 123.7, 126.6, 127.0, 128.1, 128.9, 129.4, 133.6, 136.2, 138.7, 140.2, 157.6, 173.6, 175.4. MS (CI^+^): *m*/*z* 388 (100%), 387 (45%) 212 (90%), 185 (25%), 176 (25%), 141 (<10%), 115 (<10%). HRMS (CI) calculated for C_25_H_26_NO_3_ [M + H]^+^, *m*/*z* 388.1913; found for [M + H]^+^, *m*/*z* 388.1924.

(*S,S*)-**10d**: ${\left[\alpha \right]}_{\text{D}}^{20}$ + 83.53 (*c* 0.8, CHCl_3_). IR (cm^−1^): 2931, 1734, 1686, 1604, 1482, 1197, 727, 701, 672. ^1^H-NMR (CDCl_3_, 400 MHz) δ: 1.55 (d, *J* = 7.2 Hz, 3H), 2.14 (s, 3H), 2.60 (dd, *J* = 17.6, 8.4 Hz, 1H), 2.91 (dd, *J* = 17.6, 8.4 Hz, 1H), 3.46 (m, 1H), 3.68 (dd, *J* = 12.0, 6.8 Hz, 1H), 3.91 (s, 3H), 4.31 (dd, *J* = 12.0, 8.0 Hz, 1H), 5.23 (q, *J* = 7.2 Hz, 1H), 6.74–6.80 (m, 2H), 6.97–6.98 (m, 2H), 7.11–7.13 (m, 2H), 7.45–7.48 (m, 1H), 7.68 (d, *J* = 8.4 Hz, 3H). ^13^C-NMR (CDCl_3_, 100 MHz) δ: 19.4, 21.2, 35.9, 41.3, 44.6, 52.5, 53.5, 105.5, 118.8, 123.4, 126.5, 127.0, 127.1, 127.9, 128.7, 129.4, 133.6, 136.0, 138.6, 140.1, 157.6, 173.6, 175.3. MS (CI^+^): *m*/*z* 388 (100%), 387 (45%) 212 (77%), 185 (20%), 176 (12%). HRMS (CI) calculated for C_25_H_26_NO_3_ [M + H]^+^, *m*/*z* 388.1913, found for [M + H]^+^, *m*/*z* 388.1935.

### 3.9. General Procedure for the Preparation of Enantiomerically-Pure β-Aryl-γ-lactams ***2a**–**d***

To a solution of (*R*,*S*)-**10a**–**d** or (*S*,*S*)-**10a**–**d** in tetrahydrofuran (0.6 mL) was added 1 N KOH (0.3 mL) and the reaction mixture was stirred at room temperature for 5 h. The solvent was evaporated under reduced pressure and extracted with CH_2_Cl_2_ (4 × 3 mL), the organic layer was dried over anhydrous Na_2_SO_4_ and evaporated under reduced pressure to give the corresponding γ-lactams (*R*)**-2a**–**d** or (*S*)-**2a**–**d**.

#### 3.9.1. (*R*)-4-Phenylpyrrolidin-2-one **2a**

According to the general procedure (*R*,*S*)-**10a** (23 mg, 0.06 mmol) in THF (0.6 mL) was reacted with 1 N KOH (0.3 mL), to give (*R*)-**2a** (7 mg, 100%) as a white solid, m.p.: 84–86 °C, [43,44]. ${\left[\alpha \right]}_{\text{D}}^{20}$ − 19.2 (*c* 0.9, CHCl_3_) [31]. ^1^H- and ^13^C-NMR data are identical to (±)-**2a**.

#### 3.9.2. (*S*)-4-Phenylpyrrolidin-2-one **2a**

According to the general procedure (*S,S*)-**10a** (20 mg, 0.056 mmol) in THF (0.6 mL) was reacted with 1 N KOH (0.3 mL) to give (*S*)*-***11a** (8 mg, 95%) as a white solid, m.p.: 87–89 °C [43,44]. ${\left[\alpha \right]}_{\text{D}}^{20}$ + 19.8 (*c* 0.9, CHCl_3_). ^1^H- and ^13^C-NMR data are identical to (*R*)-**2a**.

#### 3.9.3 (*R*)-4-(4-Chlorophenyl)-pyrrolidin-2-one **2b**

According to general procedure (*R*,*S*)-**10b** (19 mg, 0.05 mmol) in THF (0.5 mL) was reacted with 1 N KOH (0.3 mL) to give (*R*)*-***11b** (7 mg, 79%) as a white solid, m.p.: 102–105 °C. ${\left[\alpha \right]}_{\text{D}}^{20}$ − 24.2 (*c* 1.15, CHCl_3_) [31]. ^1^H- and ^13^C-NMR data are identical to (±)-**2b**.

#### 3.9.4. (*S*)-4-(4-Chlorophenyl)-pyrrolidin-2-one **2b**

According to the general procedure (*S,S*)-**10b** (28 mg, 0.07 mmol) in THF (0.5 mL) was reacted with 1 N KOH (0.3 mL) to give (*S*)*-***2b** (12 mg, 94%) as a white solid, m.p.: 99–101 °C. ${\left[\alpha \right]}_{\text{D}}^{20}$ + 13.5 (*c* 0.9, CHCl_3_) [45]. ^1^H- and ^13^C-NMR data are identical to (±)-**2b**.

#### 3.9.5. (*R*)-4-(4-Methylphenyl)-pyrrolidin-2-one **2c**

According to general procedure (*R*,*S*)-**10c** (30 mg, 0.06 mmol) in THF (0.6 mL) was reacted with 1 N KOH (0.3 mL) to give (*R*)*-***2c** (12 mg, 98%) as a white solid, m.p.: 108–110 °C. ${\left[\alpha \right]}_{\text{D}}^{20}$ − 33.7 (*c* 0.95, CHCl_3_). IR (cm^−1^): 3189, 2917, 1685, 804. ^1^H-NMR (CDCl_3_, 400 MHz) δ: 2.28 (s, 3H), 2.48 (dd, *J* = 17.2, 9.2 Hz, 1H), 2.71 (dd, *J* = 17.2, 8.4 Hz, 1H), 3.39 (dd, *J* = 9.2, 7.6 Hz, 1H), 3.66 (dddd, *J* = 9.2, 8.4, 8.0, 7.6 Hz, 1H), 3.73–3.80 (m, 1H), 7.1 (s, 4H). ^13^C-NMR (CDCl_3_, 100 MHz): δ 21.2, 38.1, 40.2, 49.8, 126.8, 129.7, 137.0, 139.2, 177.9. MS (FAB^+^): *m*/*z* 176 (100%), 149 (25%), 113 (<10%), 73 (<10%), 57 (<10%). HRMS (FAB) calculated for C_11_H_14_NO [M + H]^+^, *m*/*z* 176.1075; found for [M + H]^+^, *m*/*z* 176.1083.

#### 3.9.6. (*S*)-4-(4-Methylphenyl)-pyrrolidin-2-one **2c**

According to the general procedure (*S,S*)-**10c** (26 mg, 0.054 mmol) in THF (0.6 mL) was reacted with 1 N KOH (0.3 mL) to give (*S*)*-***2c** (11 mg, 100%) as a white solid, m.p.: 100–103 °C. ${\left[\alpha \right]}_{\text{D}}^{20}$ + 30.3 (*c* 1.04, CHCl_3_). IR (cm^−1^): 3191, 2918, 1685, 804. ^1^H-NMR (CDCl_3_, 200 MHz) δ: 2.33 (s, 3H), 2.47 (dd, *J* = 16.8, 7.8 Hz, 1H), 2.70 (dd, *J* = 16.8, 9.0 Hz, 1H), 3.38 (dd, *J* = 8.2, 6.6 Hz, 1H), 3.56-3.80 (m, 2H), 7.14 (s, 4H). ^13^C-NMR (CDCl_3_, 50 MHz): δ 21.6, 38.1, 40.5, 49.7, 123.9, 127.7, 128.0, 128.9, 138.7, 142.3, 177.9. MS (FAB^+^): *m*/*z* 176 (100%), 147 (76%), 73 (58%), 57 (13%). HRMS (FAB) calculated for C_11_H_14_NO [M + H]^+^, *m*/*z* 176.1075; found for [M + H]^+^, *m*/*z* 176.1043.

#### 3.9.7. (*R*)-4-(3-Methylphenyl)-pyrrolidin-2-one **2d**

According to the general procedure (*R*,*S*)*-***10d** (24 mg, 0.06 mmol) in THF (0.6 mL) was reacted with 1 N KOH (0.3 mL) to give (*R*)*-***2d** (8 mg, 72%) as an amber liquid. ${\left[\alpha \right]}_{\text{D}}^{20}$ − 20.0 (*c* 0.68, CHCl_3_). IR (cm^−1^): 3228, 2922, 1686, 784, 700, 637. ^1^H-NMR (CDCl_3_, 200 MHz) δ: 2.35 (s, 3H), 2.50 (dd, *J* = 17.2, 9.0 Hz, 3H), 2.71 (dd, *J* = 17.2, 8.6 Hz, 1H), 3.41 (dd, *J* = 8.2, 6.6 Hz, 1H), 3.62–3.70 (m, 2H), 7.04–7.09 (m, 2H), 7.21–7.26 (m, 2H). ^13^C-NMR (CDCl_3_, 50 MHz): δ 21.6, 38.0, 40.4, 49.6, 123.9, 127.7, 128.0, 128.9, 138.7, 142.3, 177.9. MS (FAB^+^): *m*/*z* 176 (20%), 175 (15%), 145 (<10%), 131 (<10%), 118 (100%), 117 (30%), 91 (15%). HRMS (FAB) calculated for C_11_H_14_NO [M + H]^+^, *m*/*z* 176.1075; found for [M + H]^+^, *m*/*z* 176.1069.

#### 3.9.8. (*S*)-4-(3-Methylphenyl)-pyrrolidin-2-one **2d**

According to general procedure (*S,S*)*-***10d**. (29 mg, 0.16 mmol) in THF (0.5 mL) was reacted with 1 N KOH (0.3 mL) to give (*S*)*-***2d** (13 mg, 100%) as an amber liquid. ${\left[\alpha \right]}_{\text{D}}^{20}$ + 17.5 (*c* 0.94, CHCl_3_). IR (cm^−1^): 3222, 2919, 1682, 783, 699, 637. ^1^H-NMR (CDCl_3_, 200 MHz) δ: 2.34 (s, 3H), 2.46 (dd, *J* = 16.8, 9.0 Hz, 1H), 2.69 (dd, *J* = 16.8, 8.6 Hz, 1H), 3.37 (dd, *J* = 8.6, 7.2 Hz, 1H), 3.53-3.76 (m, 2H), 6.66 (bs, 1H), 7.02–7.09 (m, 2H), 7.19–7.27 (m, 2H). ^13^C-NMR (CDCl_3_, 50 MHz) δ: 21.6, 38.1, 40.5, 49.7, 123.9, 127.7, 128.0, 128.9, 138.7, 142.3, 177.9. MS (FAB^+^): *m*/*z* 176 (20%), 175 (30%), 145 (<10%), 131 (<10%), 118 (100%), 117 (25%), 91 (12%). HRMS (FAB) calculated for C_11_H_14_NO [M + H]^+^, *m*/*z* 176.1075; found for [M + H]^+^, *m*/*z* 176.1076.

### 3.10. (R)-(−)-Baclofen Hydrochloride ***4***

The γ-lactam (*R*)*-***2b** (7 mg, 0.03 mmol) and 6N HCl (2 mL) was refluxed for 3.5 h. After this time, the mixture reaction was concentrated in vacuum to afford (*R*)*-***12b** (10 mg, 79%) as a colorless solid, m.p.: 190–192 °C. ${\left[\alpha \right]}_{\text{D}}^{20}$ − 2.0 (*c* 0.6, H_2_O) [31,45]. ^1^H- and ^13^C-NMR data are identical to those reported in the literature [46].

### 3.11. (S)-(+)-Baclofen Hydrochloride ***4***

The γ-lactam (*S*)*-***2b** (18 mg, 0.09 mmol) and 6N HCl (2 mL) was refluxed for 5.0 h. After this time, the mixture reaction was concentrated in vacuum to afford (*S*)-**4** (23 mg, 97%) as a white solid, m.p.: 188–189 [47]. ${\left[\alpha \right]}_{\text{D}}^{20}$ + 2.9 (*c* 0.76, H_2_O) [32,48]. ^1^H- and ^13^C-NMR data are identical to (*S*)-(+)*-*Baclofen hydrochloride [46].

## 4. Conclusions

In conclusion, we have demonstrated the utility of (*S*)-Naproxen as an excellent resolution agent of β-aryl-γ-lactams, which are easily obtained through four steps from diethyl or methyl malonate and the appropriate aromatic aldehyde. The utility of this methodology was highlighted by the preparation of enantiomerically-pure (*R*)- and (*S*)-Baclofen hydrochloride in excellent yields. Additionally, we anticipate that the use of this procedure could be used in the preparation of β-aryl-γ-lactams as key intermediates in the synthesis of compounds with important pharmacological properties.

## Supplementary Materials

Supplementary materials can be accessed at: http://www.mdpi.com/1420-3049/20/12/19830/s1.
