# Supplementary material for: Efficient Synthesis of β-Aryl-γ-lactams and Their Resolution with (S)-Naproxen: Preparation of (R)- and (S)-Baclofen
Source: Molecules. 2015 Dec 10;20(12):22028–43. doi: 10.3390/molecules201219830 (PMC6332160; doi:10.3390/molecules201219830)
Supplement: Supplementary file 1 [file molecules-20-19830-s001.pdf]

Efficient Synthesis of β-Aryl-γ-lactams and Their Resolution with (*S*)-Naproxen: Preparation of (*R*)- and (*S*)-Baclofen

Iris J. Montoya-Balbás, Berenice Valentín-Guevara, Estefanía López-Mendoza, Irma Linzaga-Elizalde, Mario Ordoñez and Perla Román-Bravo

Crystal data for $C_{13}H_{14}ClNO_3$ (**7c**) and $C_{13}H_{14}NO_3$ (**7d**)

Crystal data for $C_{13}H_{14}ClNO_3$ (**7c**). M_r = 267.70 gmol^{-1}, 0.55 × 0.38 × 0.23, monoclinic, space group P2(1)/n, a = 12.5214(17) Å, b = 7.5743(10) Å, c = 14.467(2) Å, α = 90°, β = 112.018(2)°, γ = 90°, V = 1272.0(3). Z = 4, ϱ = 1.398 gcm^{-3}, θ_{max} = 24.99°, 2230 independent reflections, R_1 = 0.0749 with $I > 2\sigma(I)$, wR_2 = 0.1860 for all data, 168 parameters. Crystallographic data for the structure reported in this paper have been deposited with the Cambridge Crystallographic Data Center as supplementary publication no. CCDC 1048101.

Crystal data for $C_{13}H_{14}NO_3$ (**7d**). M_r = 232.25 gmol^{-1}, 0.57 × 0.44 × 0.23 mm^3, monoclinic, space group P2(1)/c, a = 7.1505(10) Å, b = 7.5628(11) Å, c = 23.060(3) Å, α = 90°, β = 98.105(2)°, γ = 90°, V = 1234.6(3) Å^3. Z = 4, ϱ = 1.250 gcm^{-3}, θ_{max} = 23.99°, 1934 independent reflections, R_1 = 0.0641 with $I > 2\sigma(I)$, wR_2 = 0.1749 for all data, 156 parameters. Crystallographic data for the structure reported in this paper have been deposited with the Cambridge Crystallographic Data Center as supplementary publication No. CCDC 1048102.

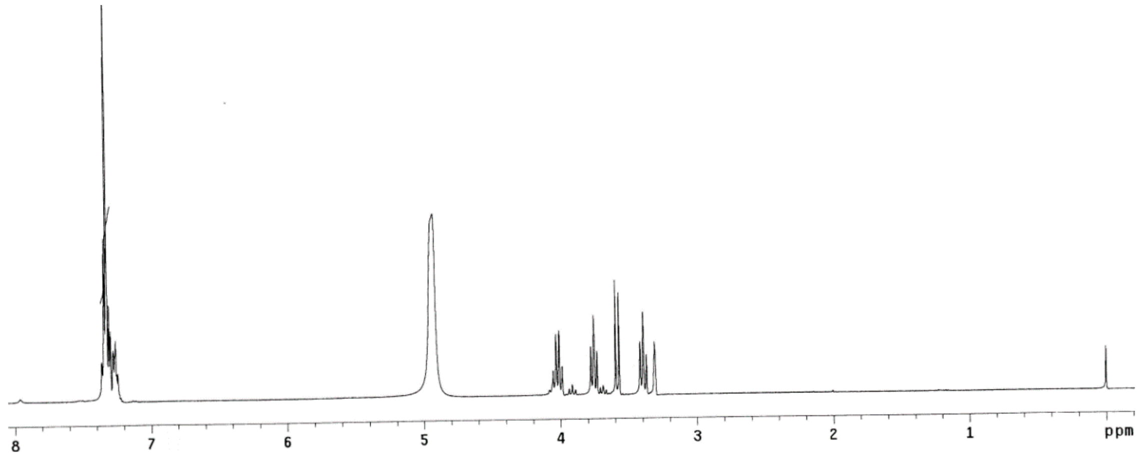

Figure S1. ^{1}H-NMR of Diethyl 2-(2-chlorobenzylidene)malonate **5e**.

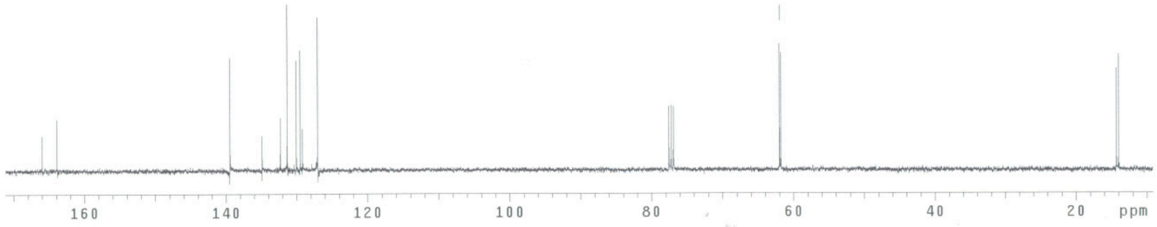

Figure S2. ^{13}C-NMR of Diethyl 2-(2-chlorobenzylidene)malonate **5e**.

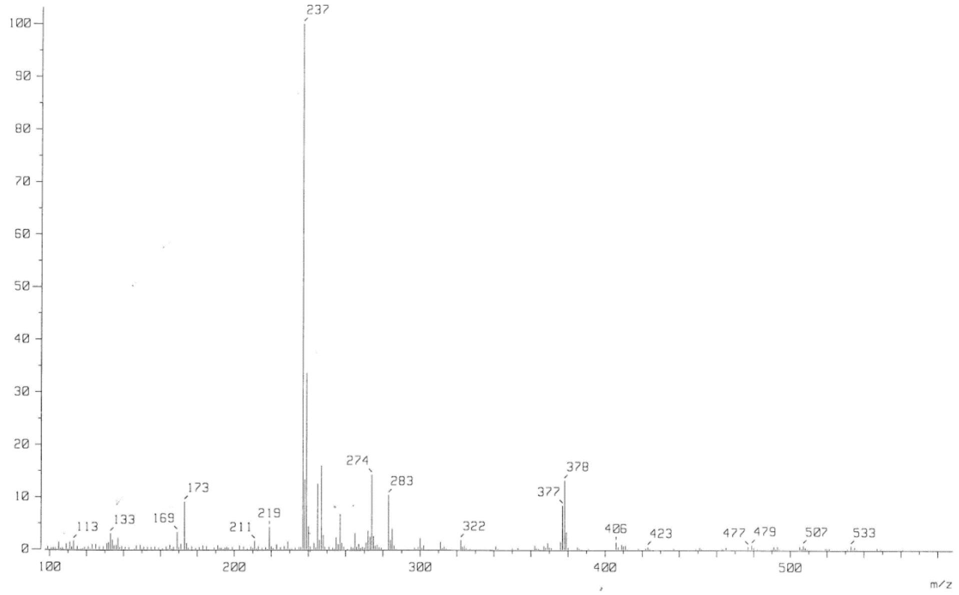

Figure S3. HRMS of Diethyl 2-(2-chlorobenzylidene)malonate **5e**.

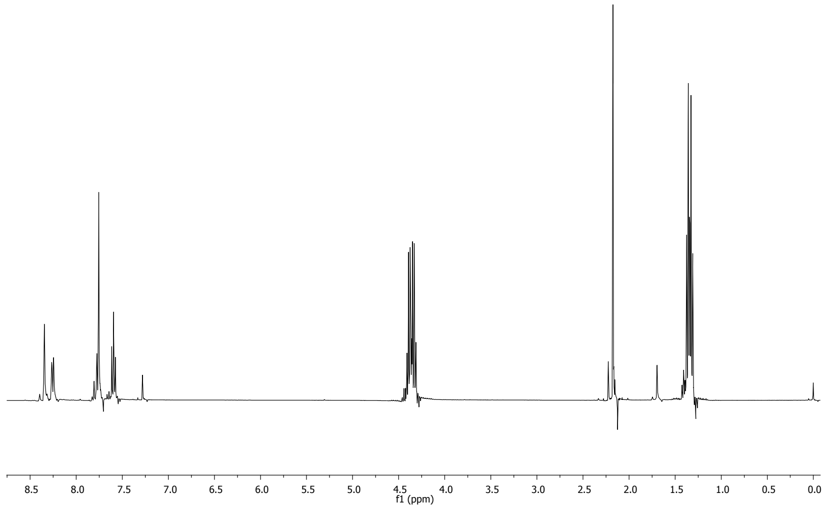

Figure S4. ^{1}H-NMR of Diethyl 2-(2-nitrobenzyliden)malonate **5f**.

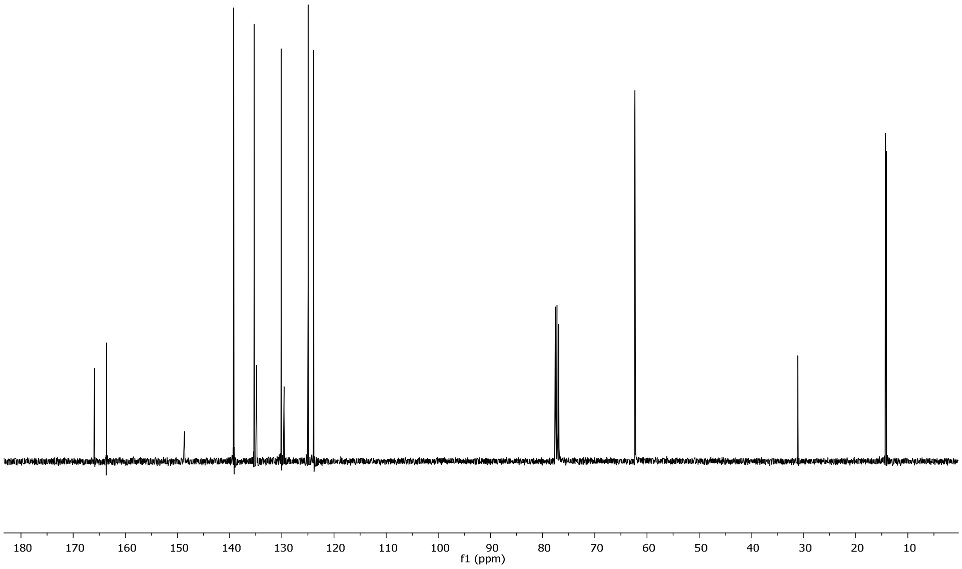

Figure S5. ^{1}H-NMR of Diethyl 2-(2-nitrobenzyliden)malonate **5f**.

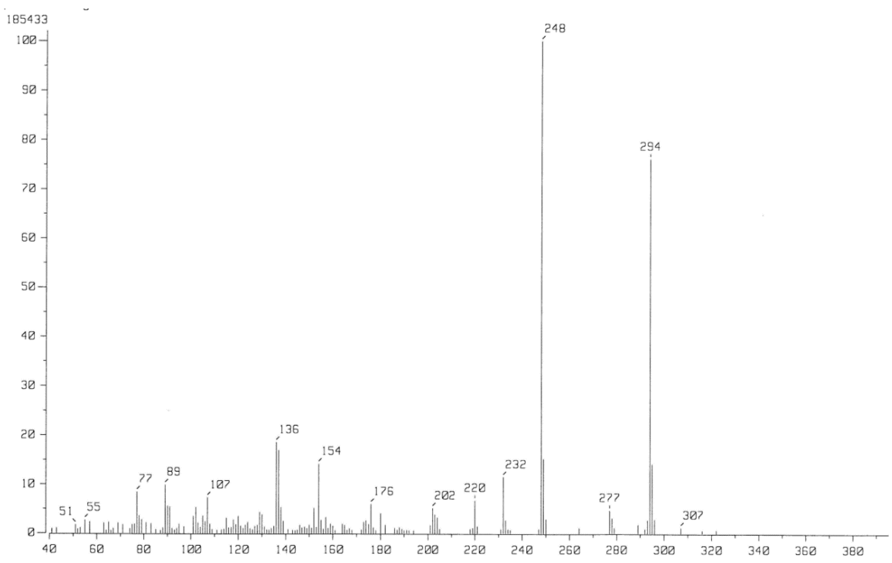

Figure S6. HRMS of Diethyl 2-(2-nitrobenzyliden)malonate **5f**.

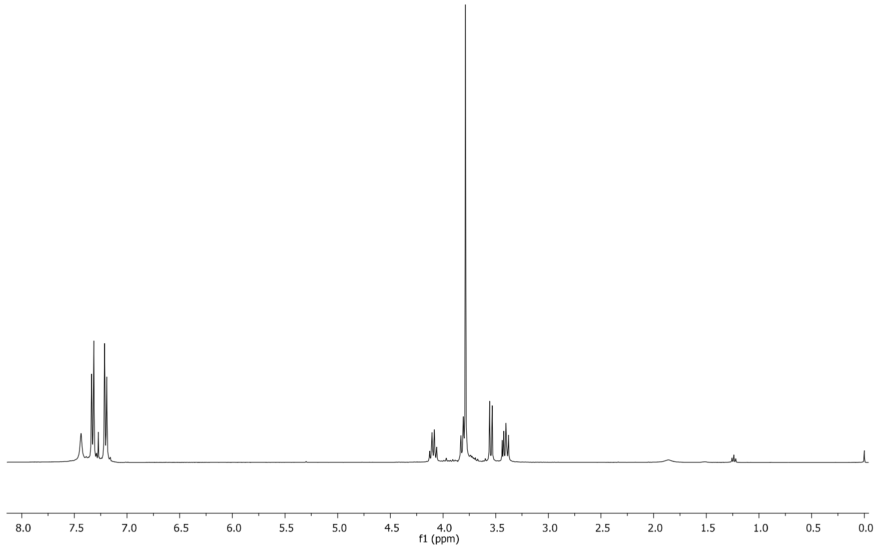

Figure S7. ^{1}H-NMR of Ethyl 4-(4-chlorophenyl)-2-oxopyrrolidine-3-carboxylate (±)-**4b.**

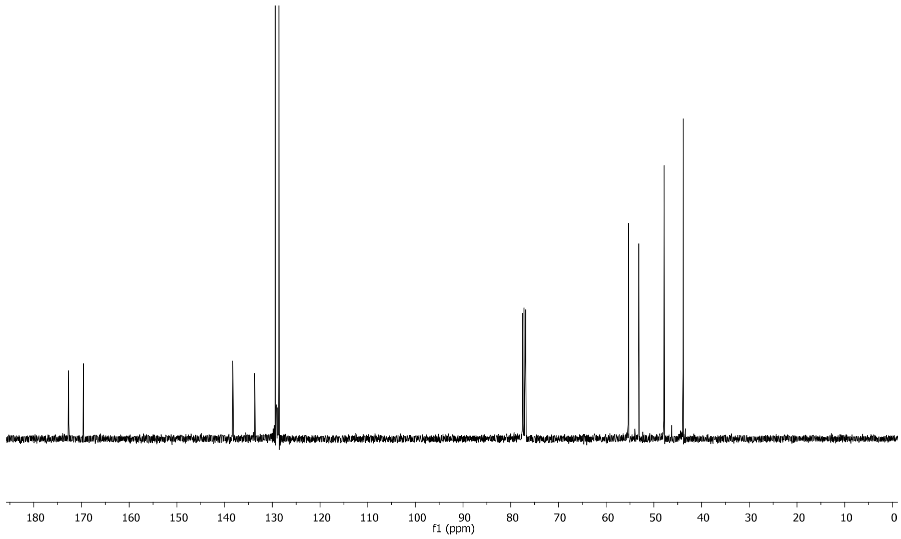

Figure S8. ^{13}C-NMR of Ethyl 4-(4-chlorophenyl)-2-oxopyrrolidine-3-carboxylate (±)-**4b.**

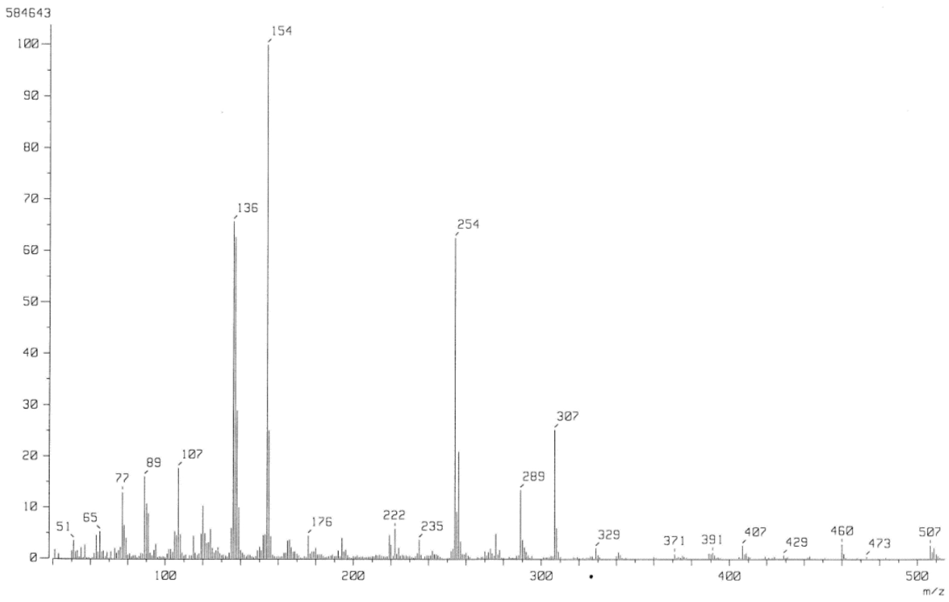

Figure S9. HRMS of Ethyl 4-(4-chlorophenyl)-2-oxopyrrolidine-3-carboxylate (±)-**4b.**

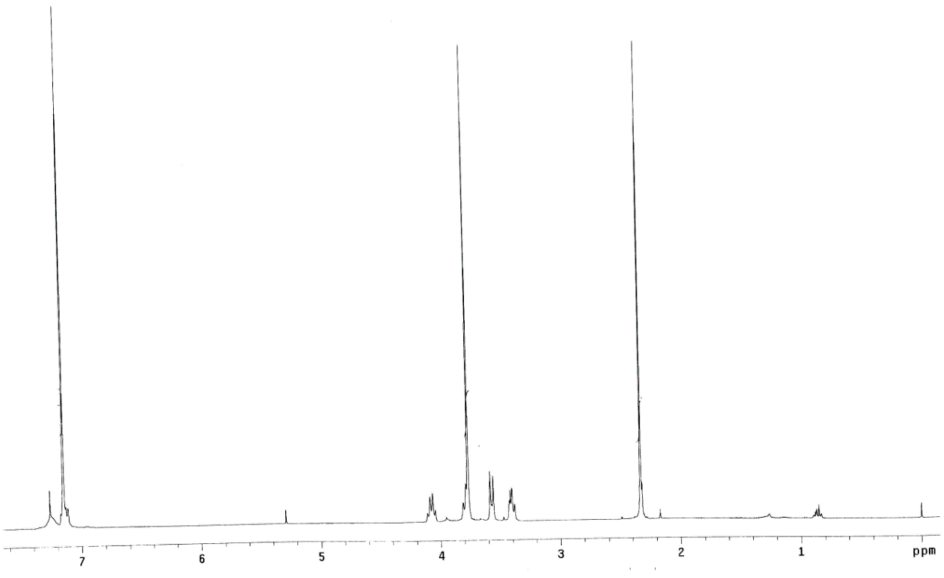

Figure S10. ^{1}HNMR Methyl 2-oxo-4(p-tolyl)pyrrolidine-3-carboxylate (±)-**4c**.

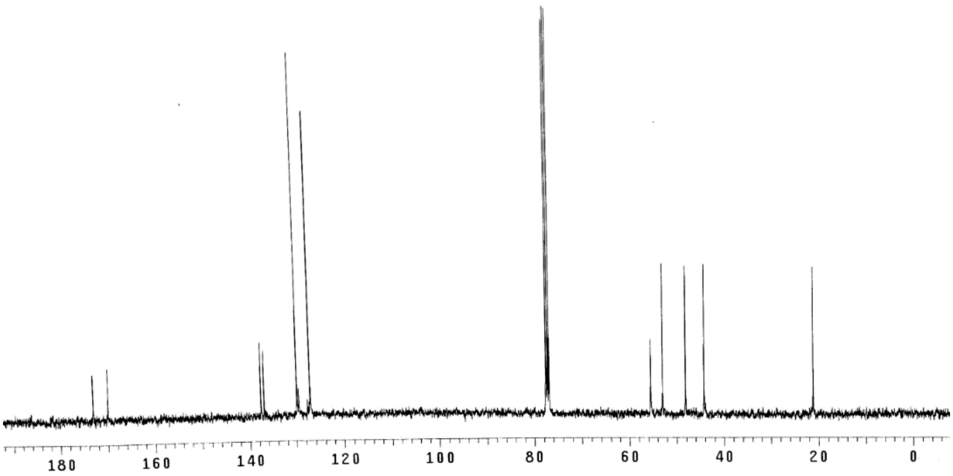

Figure S11. ^{13}C-NMR of Methyl 2-oxo-4(p-tolyl)pyrrolidine-3-carboxylate (±)-**4c**.

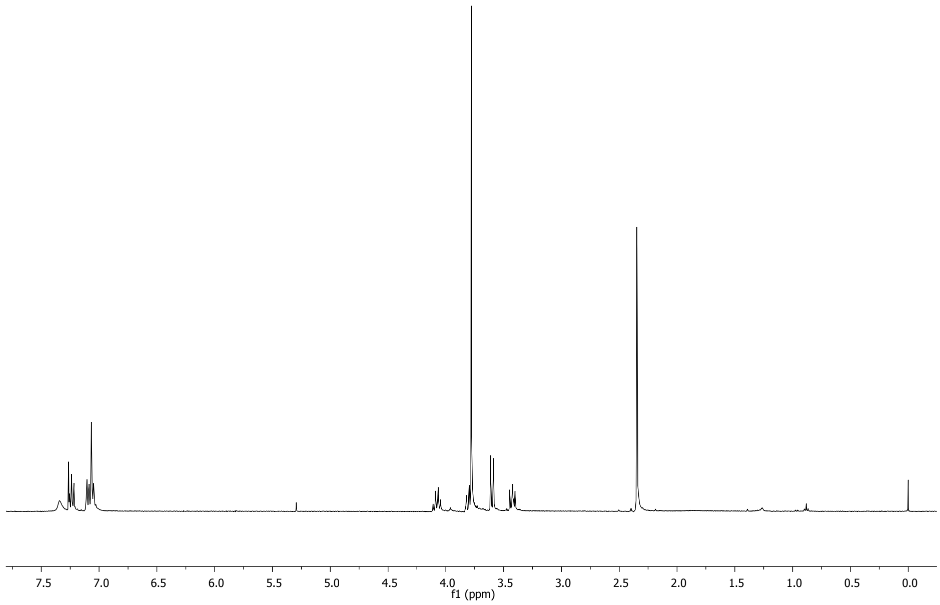

Figure S13. ^{1}H-NMR of Methyl 2-oxo-4(m-tolyl)pyrrolidine-3-carboxylate (±)-**7d**.

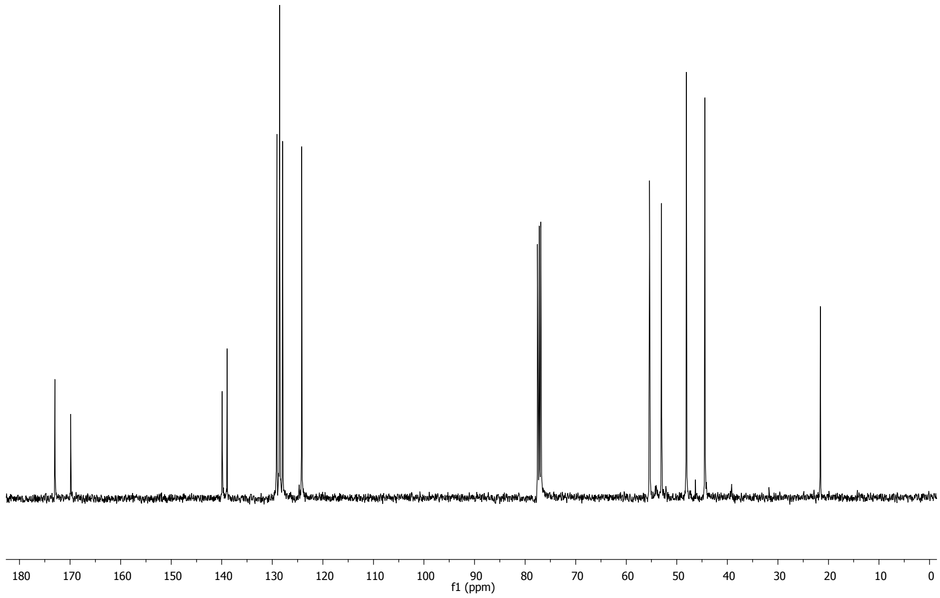

Figure S14. ^{13}C-NMRof Methyl 2-oxo-4(m-tolyl)pyrrolidine-3-carboxylate (±)-**7d**.

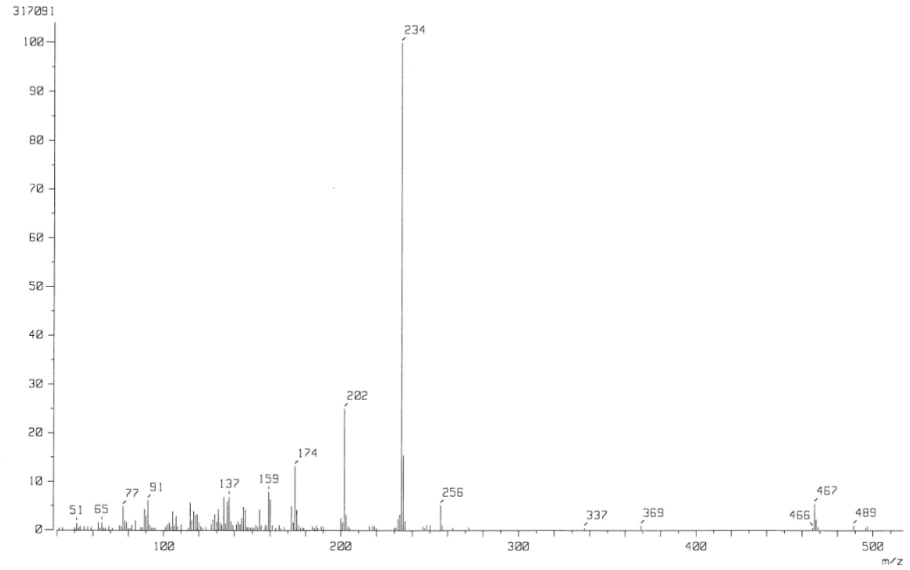

Figure S15. HRMS of Methyl 2-oxo-4(m-tolyl)pyrrolidine-3-carboxylate (±)-**7d**.

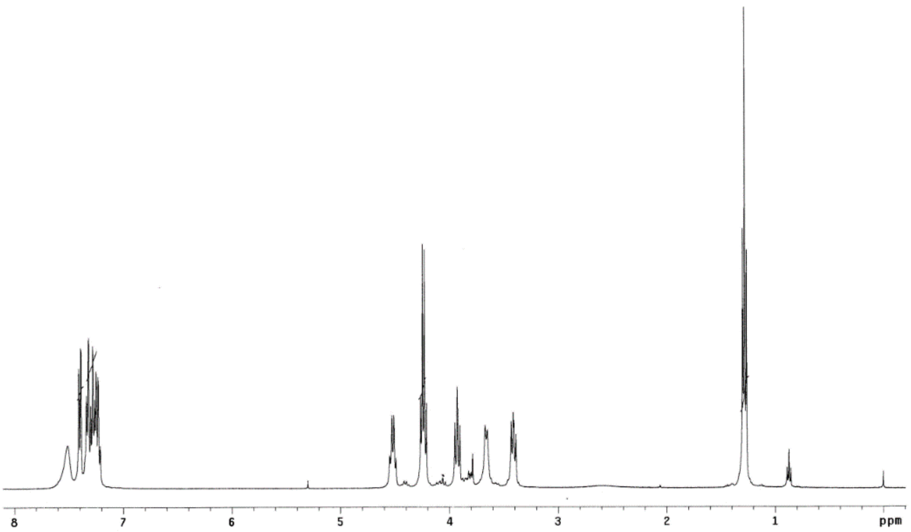

Figure S16. ^{1}H-NMR of Methyl Ethyl 2-oxo-4-(2-chlorophenyl)-pyrrolidine-3-carboxylate (±)-**7e.**

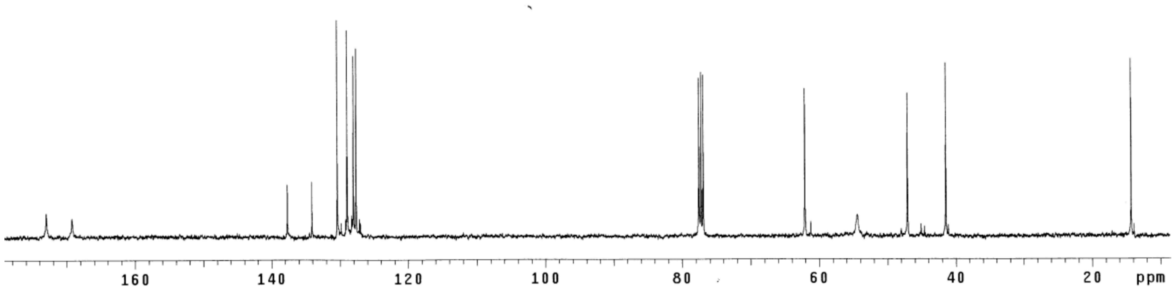

Figure S17. ^{13}C-NMR of Methyl Ethyl 2-oxo-4-(2-chlorophenyl)-pyrrolidine-3-carboxylate (±)-**7e.**

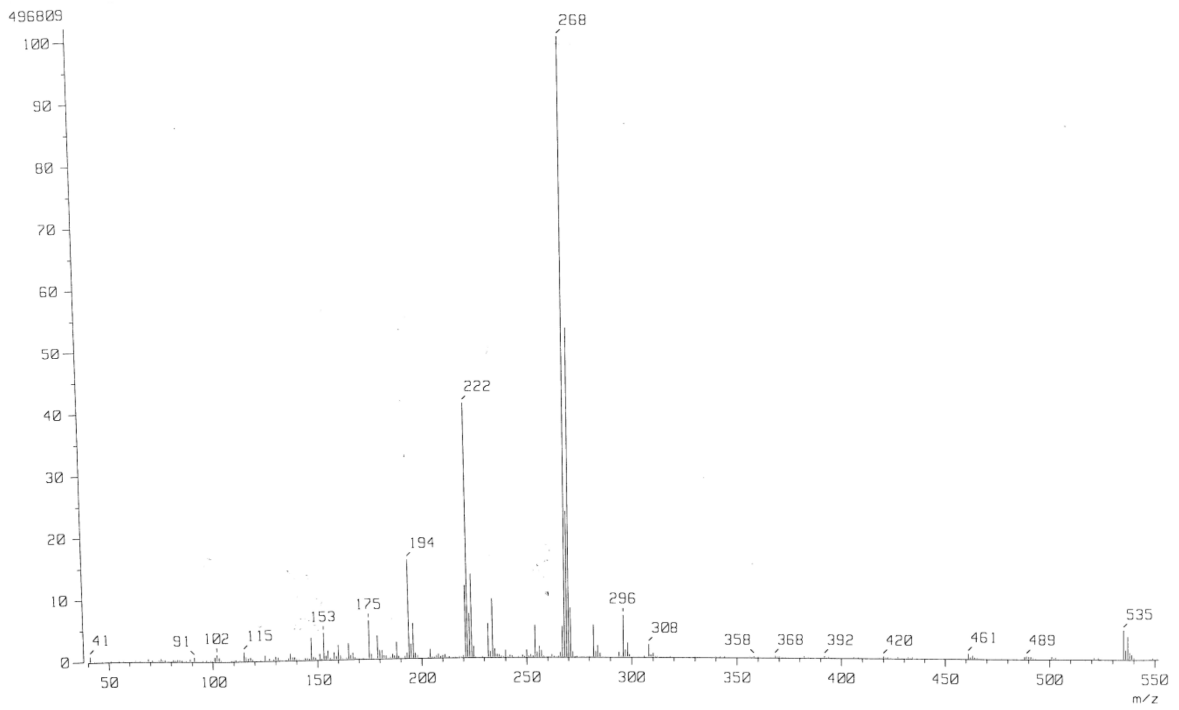

Figure S18. HRMS of Methyl Ethyl 2-oxo-4-(2-chlorophenyl)-pyrrolidine-3-carboxylate (±)-**7e.**

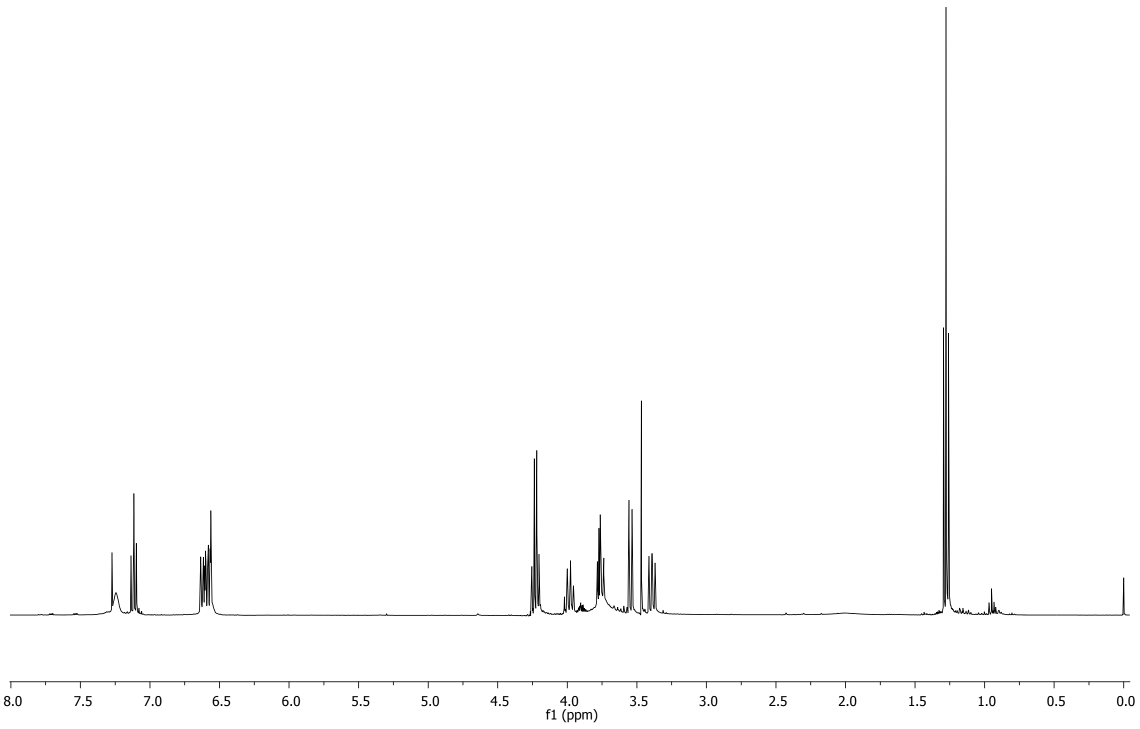

Figure S19. ^{1}H-NMR of Ethyl 2-oxo-4-(2-aminophenyl)-pyrrolidine-3-carboxylate (±)-**7f**.

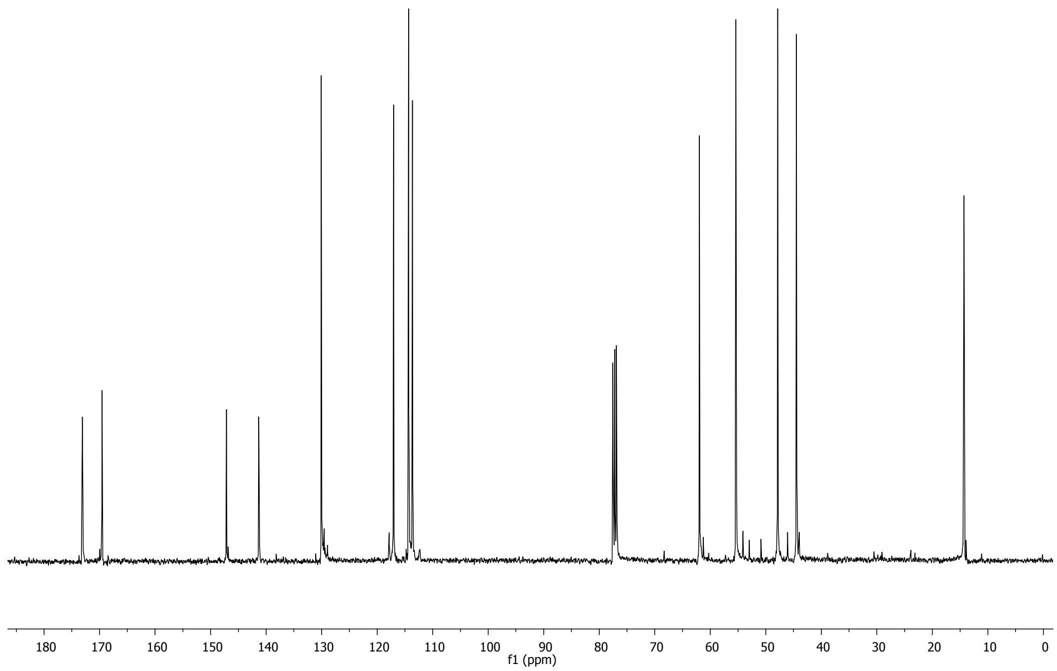

Figure S20. ^{13}C-NMR of Ethyl 2-oxo-4-(2-aminophenyl)-pyrrolidine-3-carboxylate (±)-**7f**.

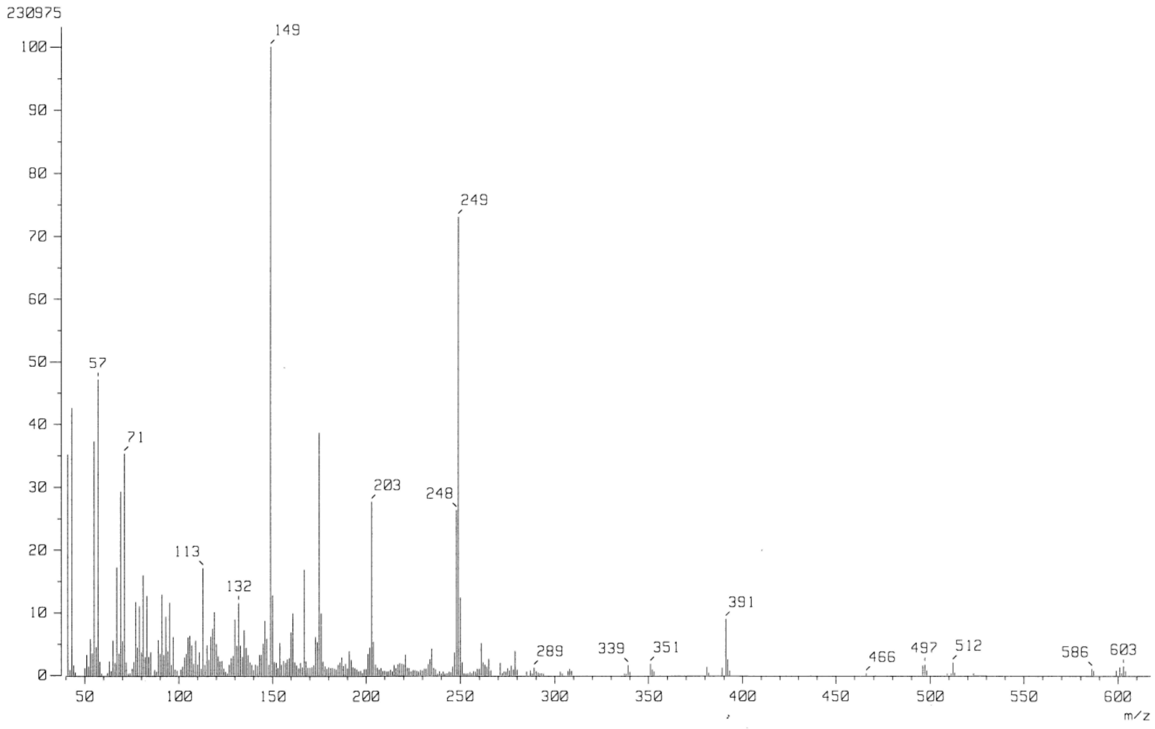

Figure S21. HRMS of Ethyl 2-oxo-4-(2-aminophenyl)-pyrrolidine-3-carboxylate (±)-**7f**.

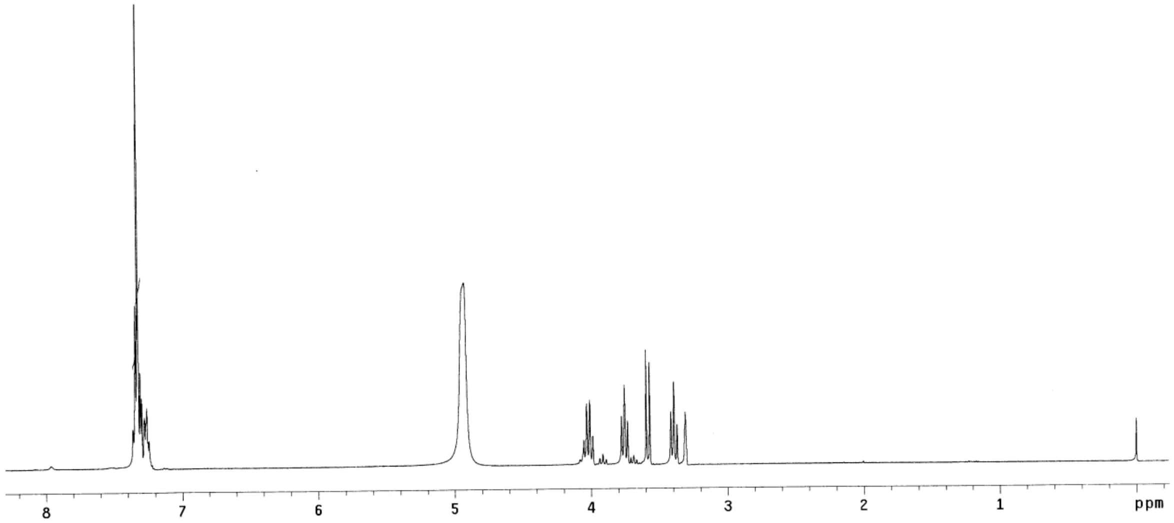

Figure S22. ^{1}H-NMR of 2-Oxo-4-phenyl-pyrrolidine-3-carboxylic acid **8a**.

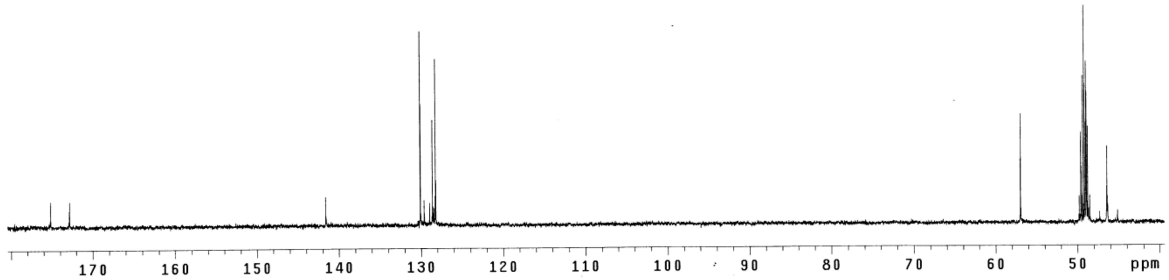

Figure S23. ^{13}C-NMR of 2-Oxo-4-phenyl-pyrrolidine-3-carboxylic acid **8a**.

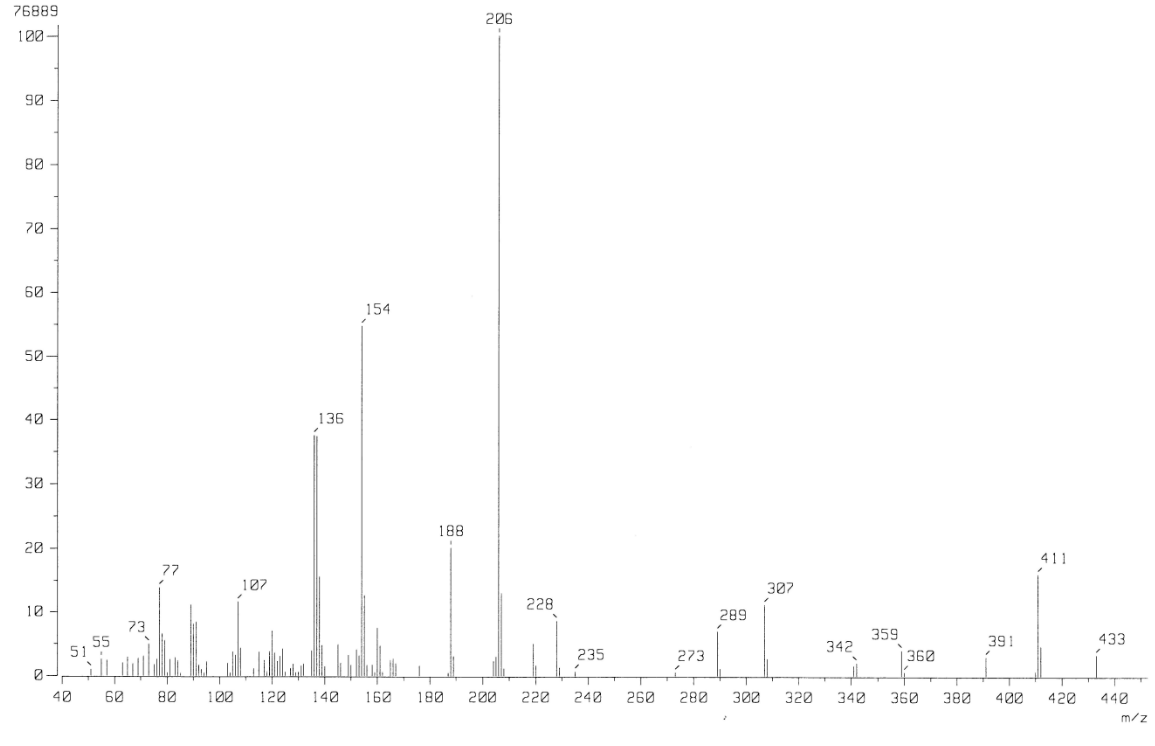

Figure S24. HRMS of 2-Oxo-4-phenyl-pyrrolidine-3-carboxylic acid **8a**.

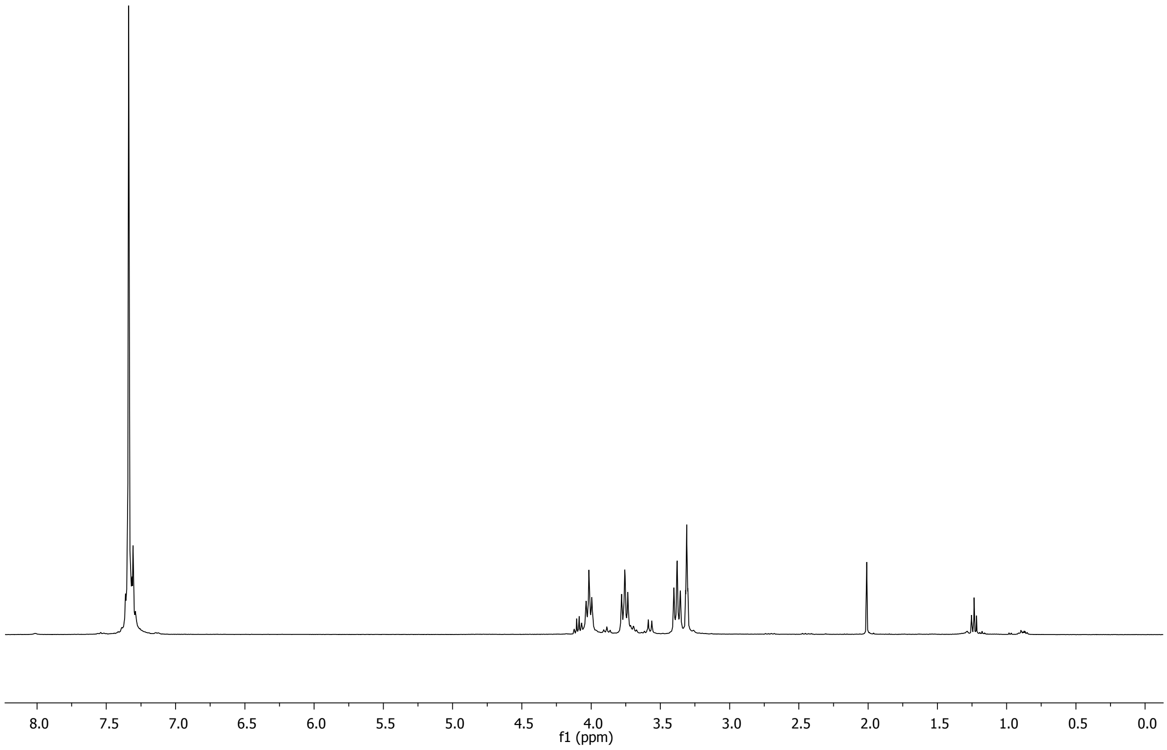

Figure S25. ^{1}H-NMR of 2-Oxo-4-(4-chlorophenyl)-pyrrolidine-3-carboxylic acid **8b**.

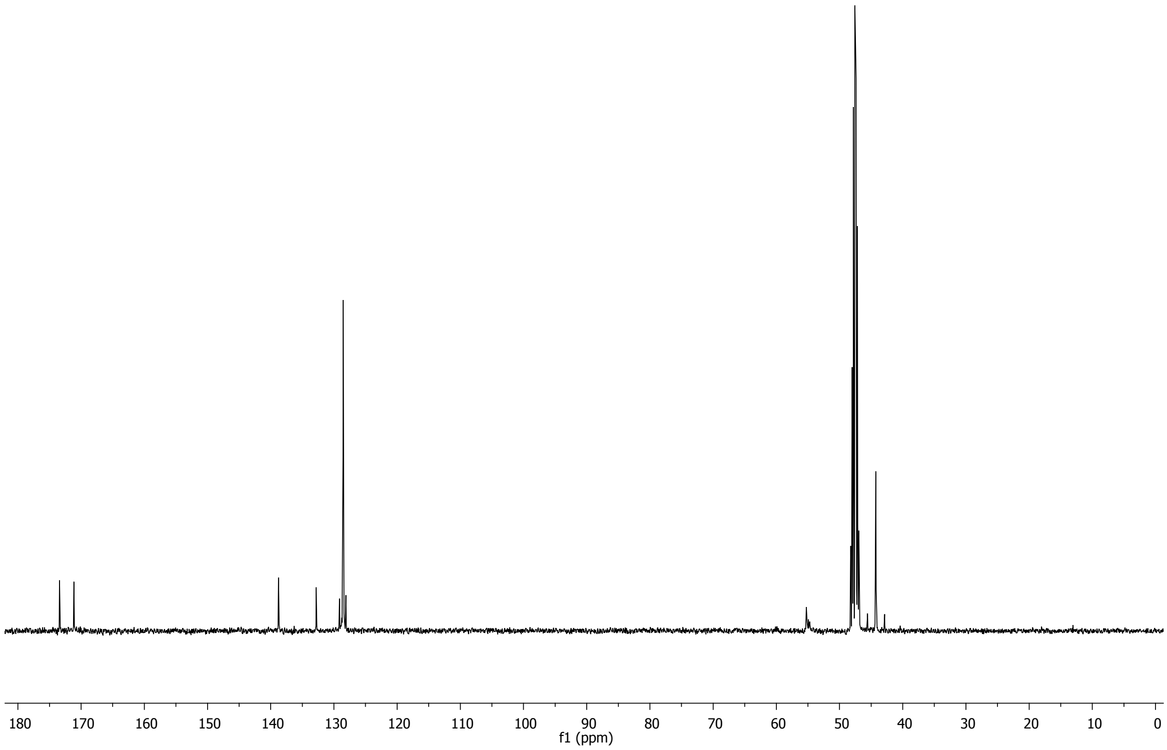

Figure S26. ^{13}C-NMR of 2-Oxo-4-(4-chlorophenyl)-pyrrolidine-3-carboxylic acid **8b**.

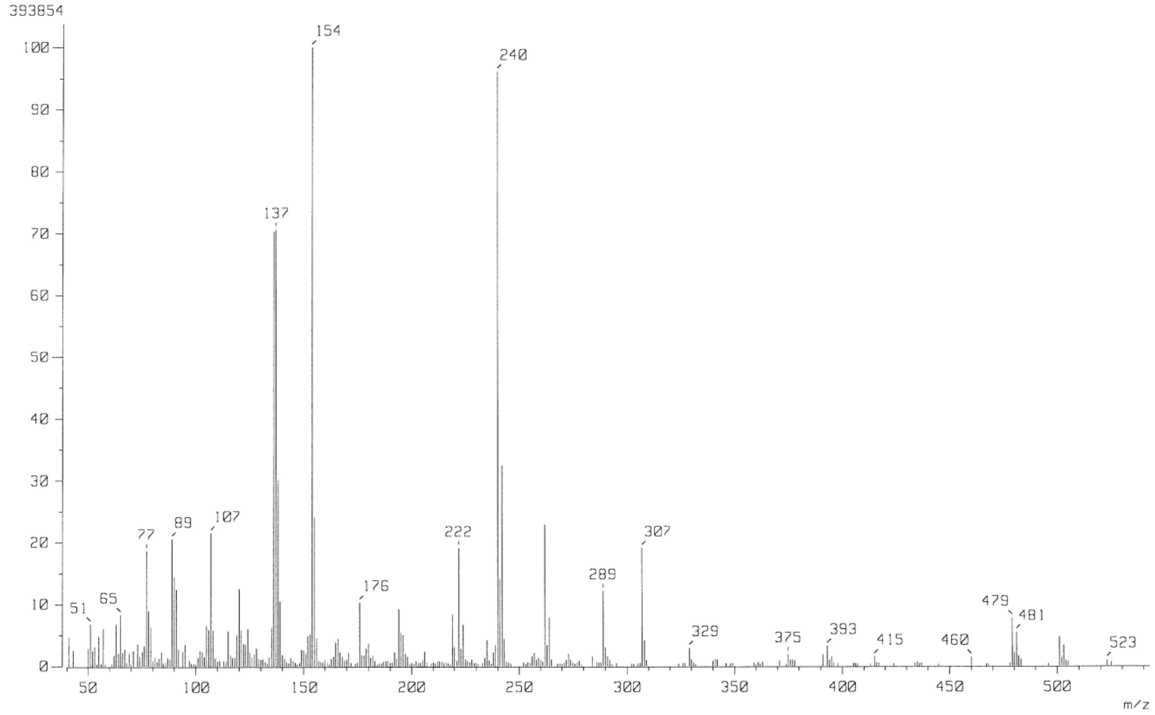

Figure S27. HRMS of 2-Oxo-4-(4-chlorophenyl)-pyrrolidine-3-carboxylic acid **8b**.

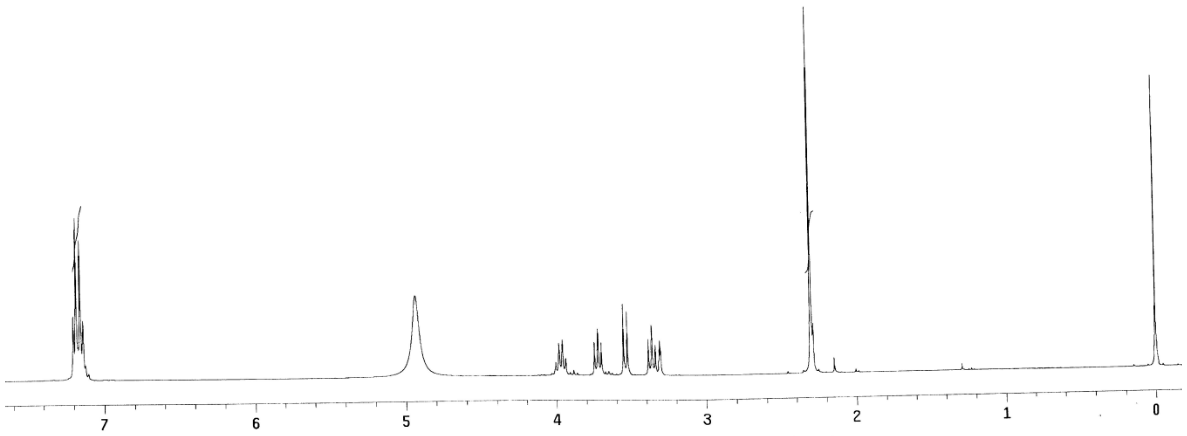

Figure S28. ^{1}H-NMR of 2-Oxo-4-(4-methylphenyl)-pyrrolidine-3-carboxylic acid **8c**.

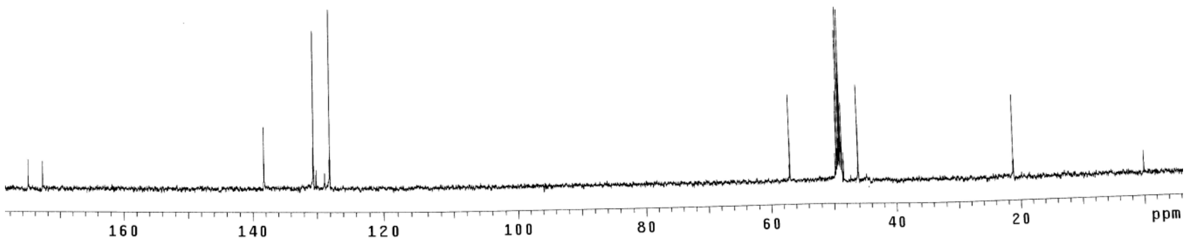

Figure S29. ^{13}C-NMR of 2-Oxo-4-(4-methylphenyl)-pyrrolidine-3-carboxylic acid **8c**.

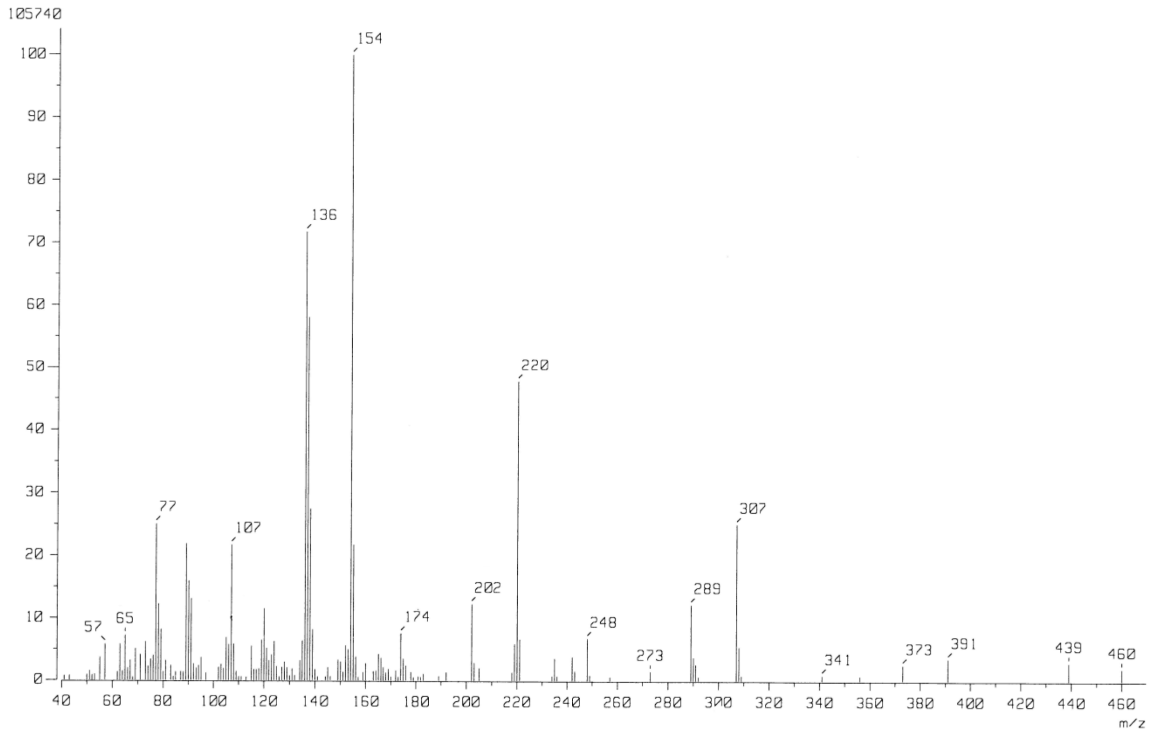

Figure S30. HRMS of 2-Oxo-4-(4-methylphenyl)-pyrrolidine-3-carboxylic acid **8c**.

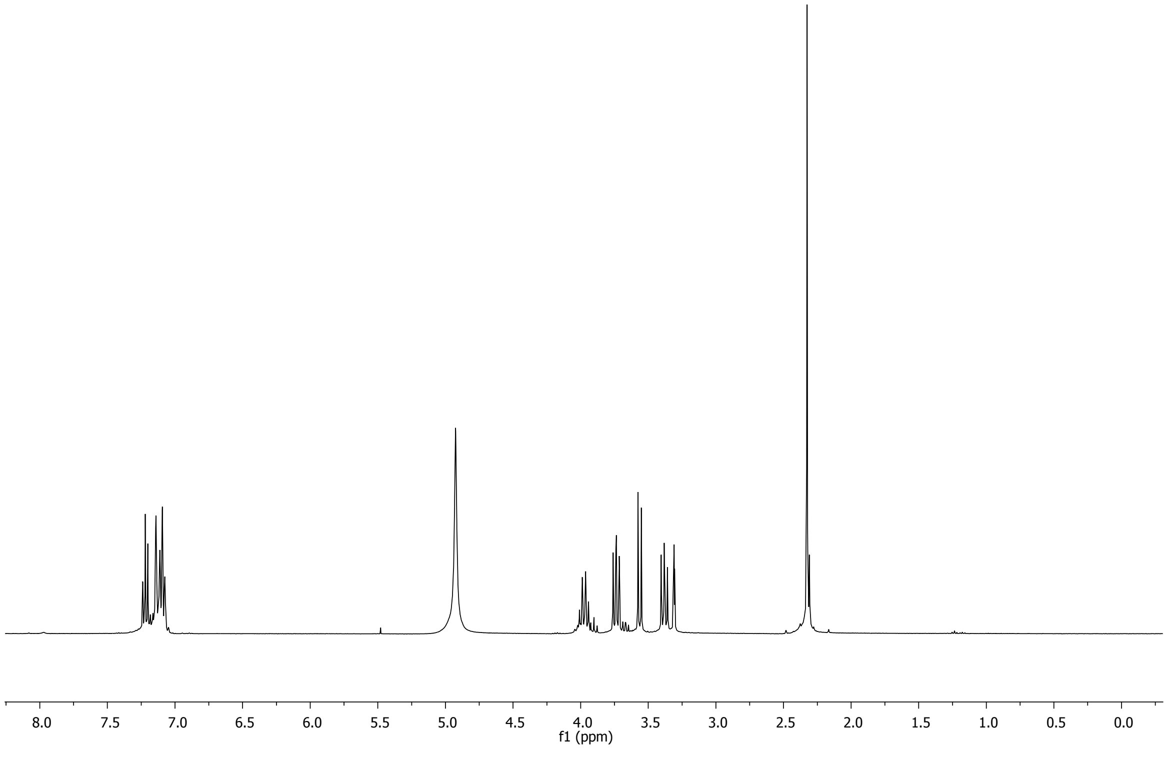

Figure S31. ^{1}H-NMR of 2-Oxo-4-(3-methylphenyl)-pyrrolidine-3-carboxylic acid **8d.**

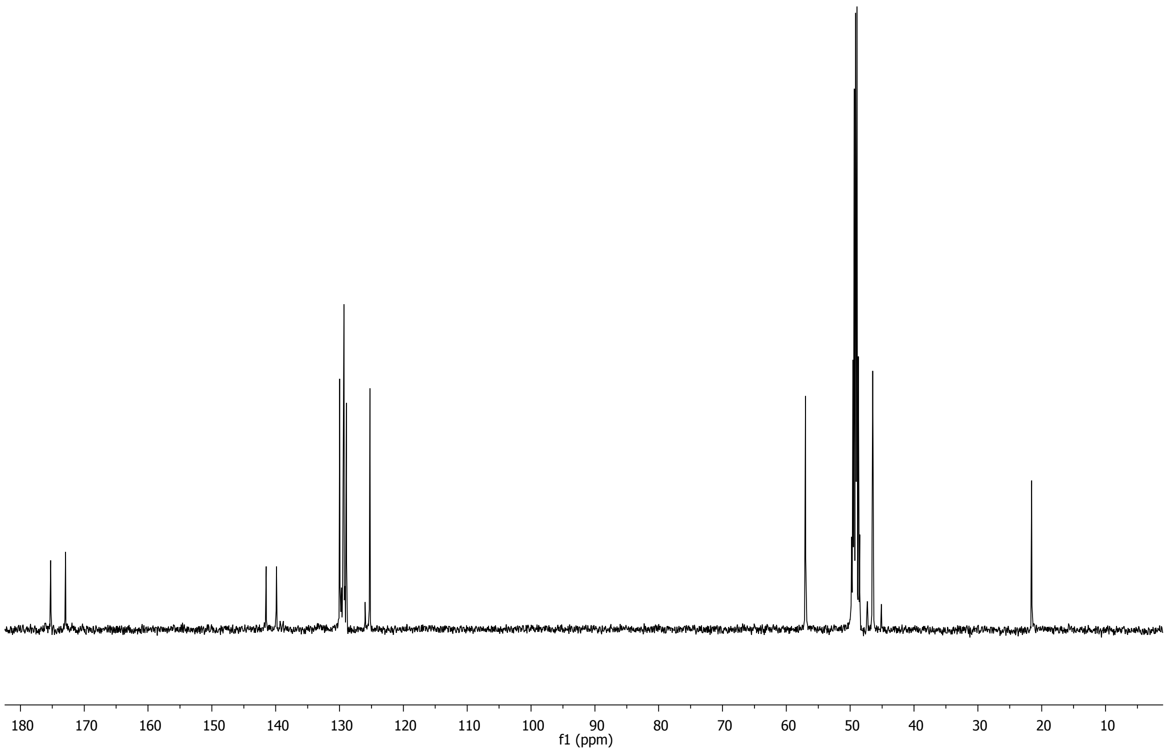

Figure S32. ^{13}C-NMR of 2-Oxo-4-(3-methylphenyl)-pyrrolidine-3-carboxylic acid **8d.**

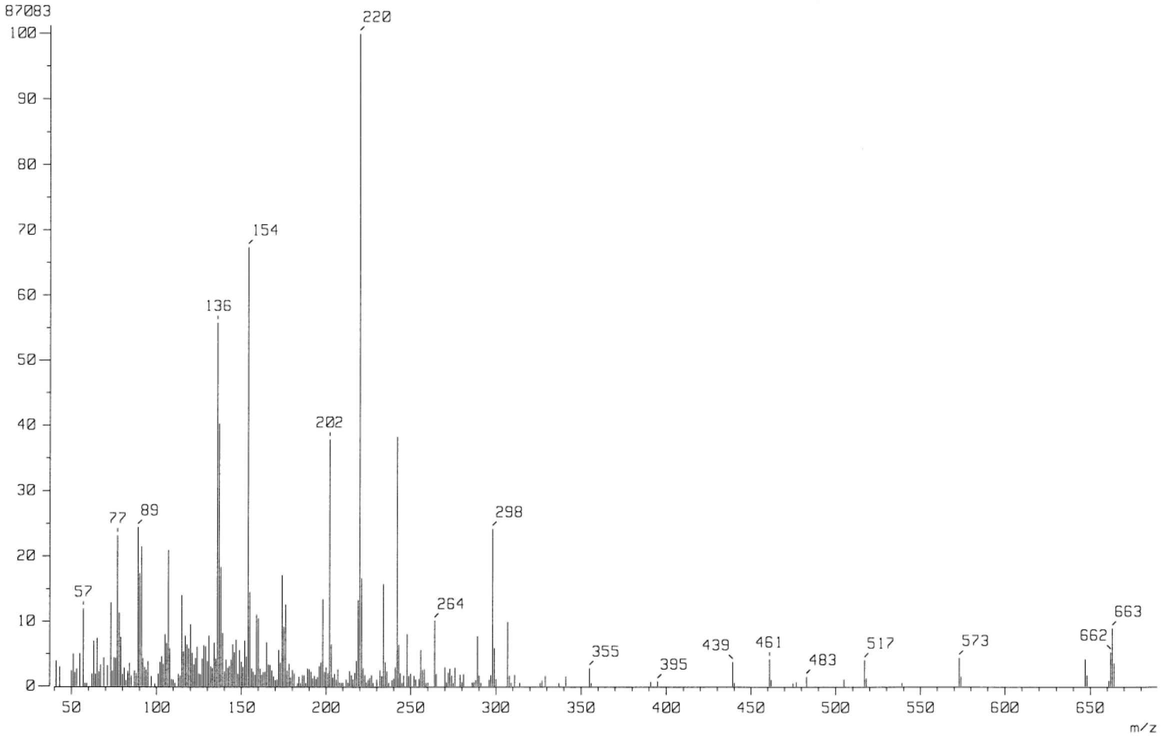

Figure S33. ^{1}H-NMR of 2-Oxo-4-(3-methylphenyl)-pyrrolidine-3-carboxylic acid **8d.**

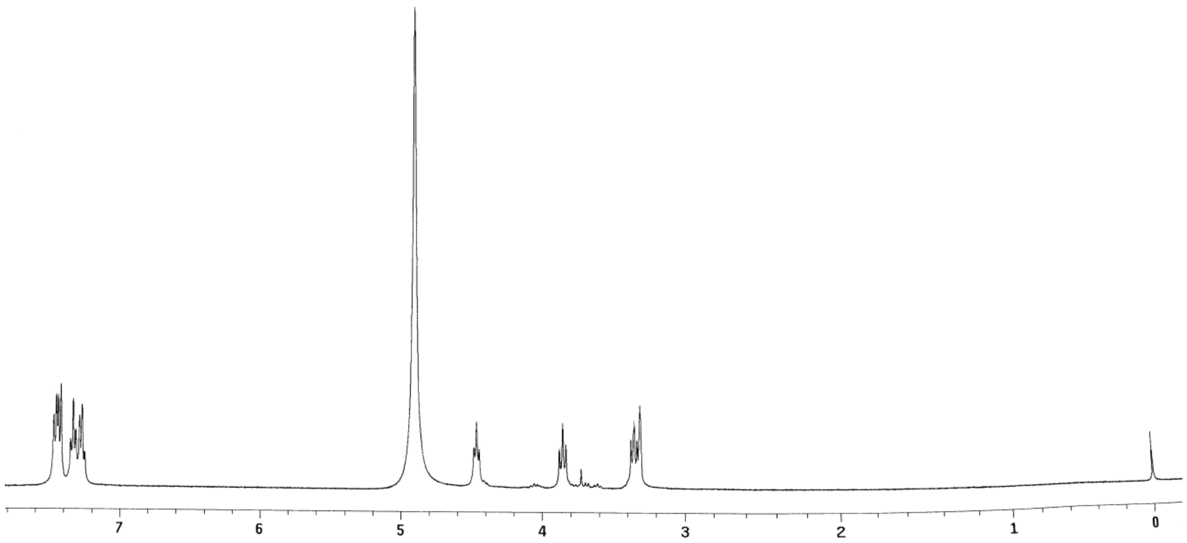

Figure S34. ^{1}H-NMR of 2-Oxo-4-(2-clorophenyl)-pyrrolidine-3-carboxylic acid **8e**.

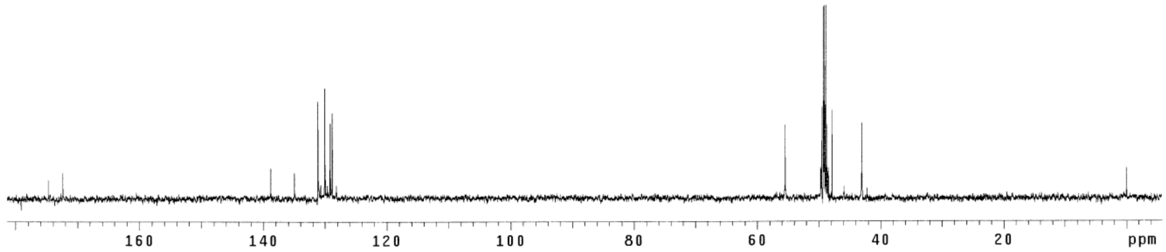

Figure S35. ^{13}C-NMR of 2-Oxo-4-(2-clorophenyl)-pyrrolidine-3-carboxylic acid **8e**.

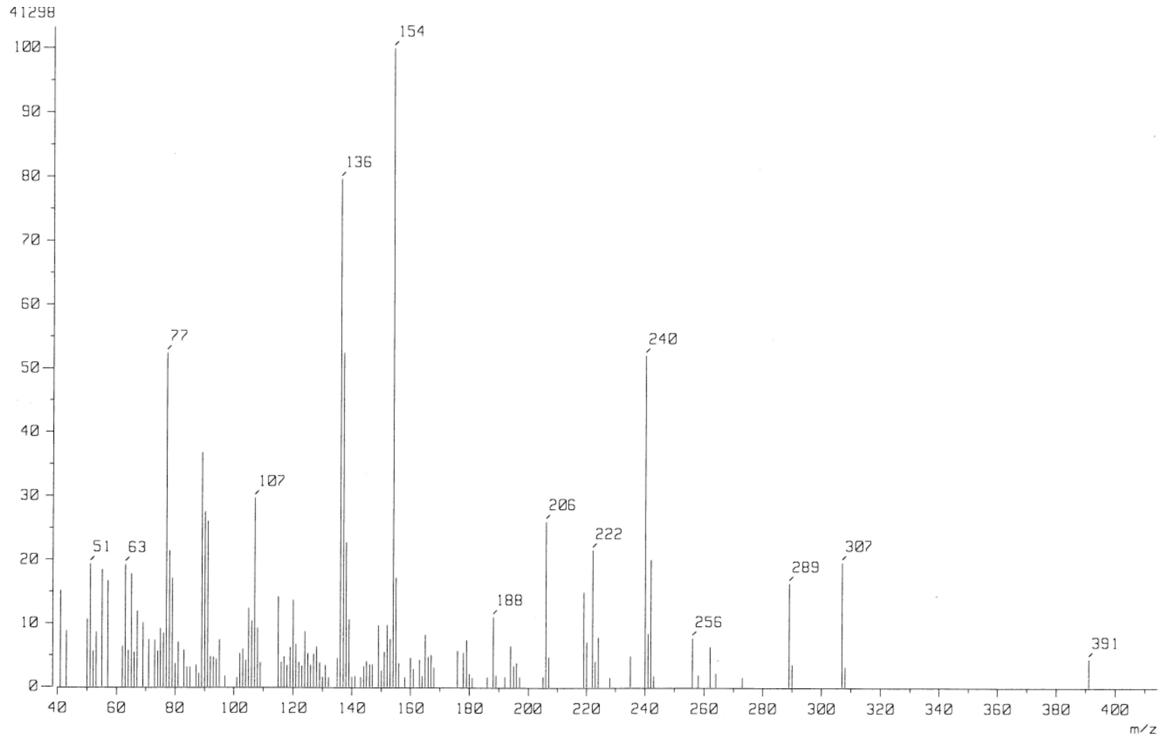

Figure S36. HRMS of 2-Oxo-4-(2-clorophenyl)-pyrrolidine-3-carboxylic acid **8e**.

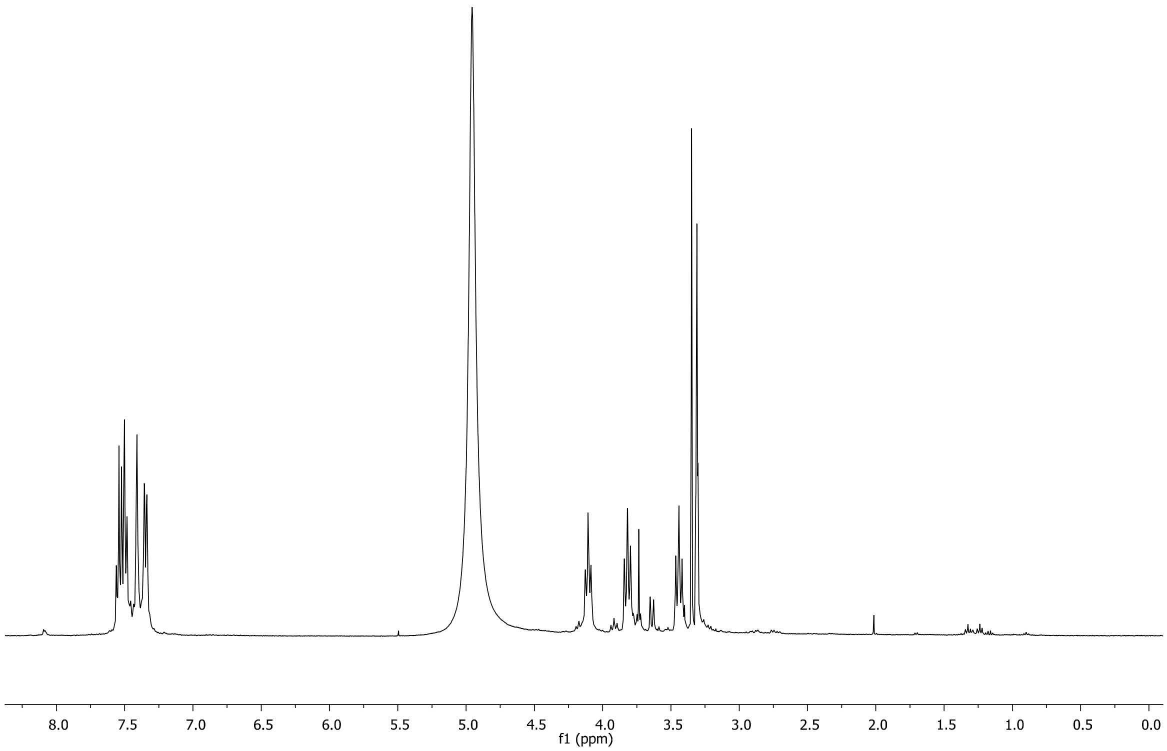

Figure S37. ^{1}H-NMR of 2-Oxo-4-(2-aminophenyl)-pyrrolidine-3-carboxylic acid **8f.**

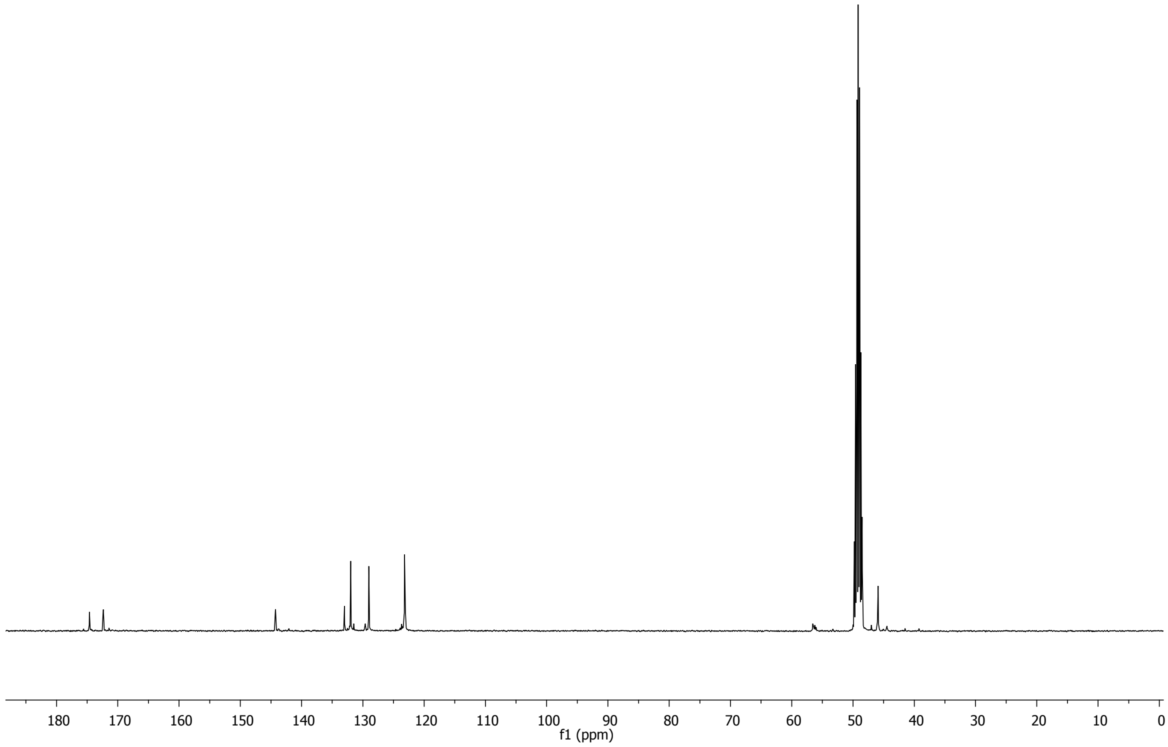

Figure S38. ^{13}C-NMR of 2-Oxo-4-(2-aminophenyl)-pyrrolidine-3-carboxylic acid **8f.**

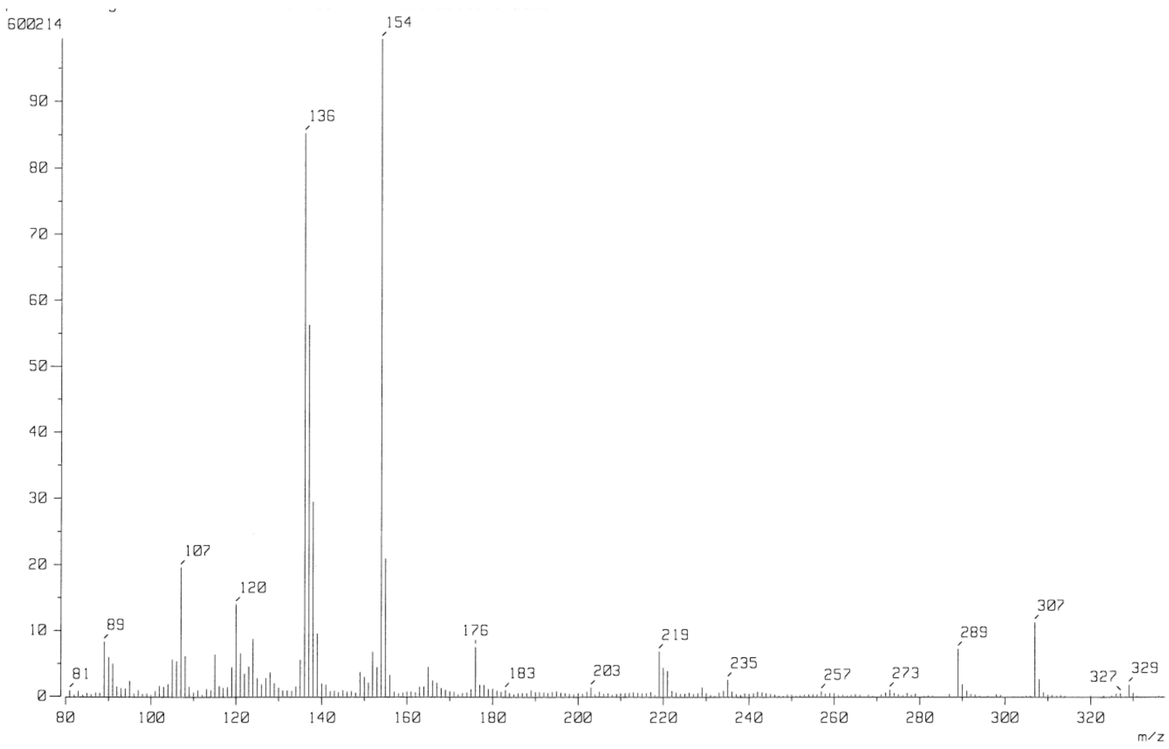

Figure S37. HRMS of 2-Oxo-4-(2-aminophenyl)-pyrrolidine-3-carboxylic acid **8f.**

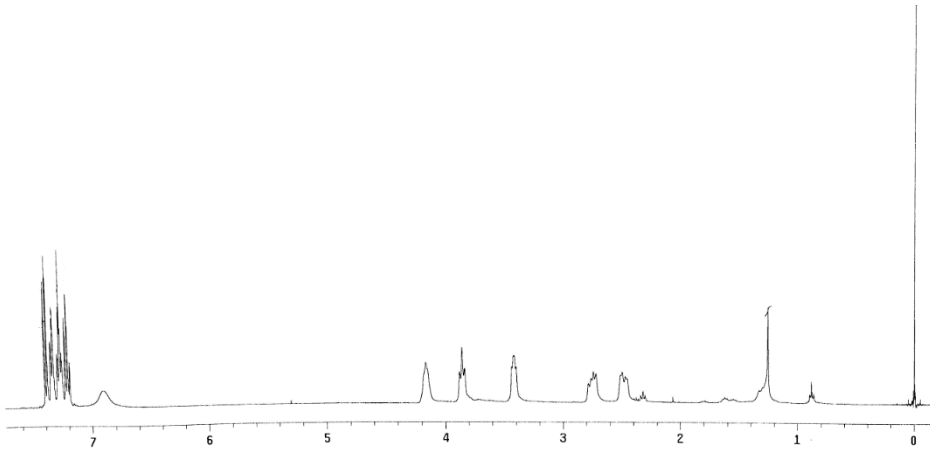

Figure S38. ^{1}H-NMR of 4-(2-Chlorophenyl)-pyrrolidin-2-one (±)-**2e**.

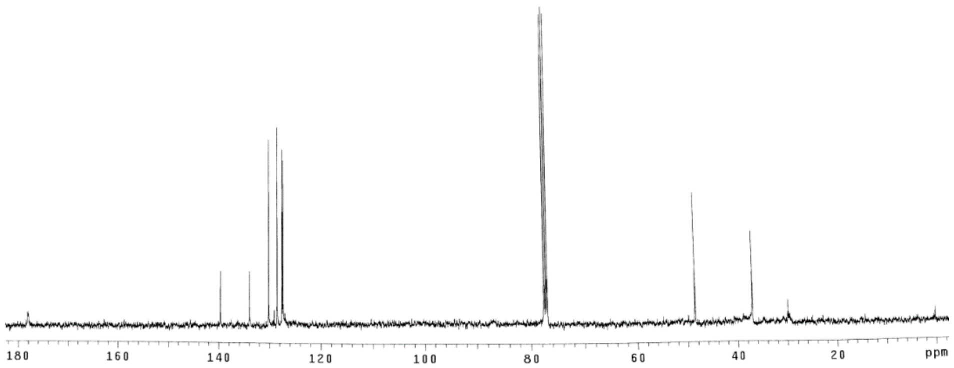

Figure S39. ^{13}C-NMR of 4-(2-Chlorophenyl)-pyrrolidin-2-one (±)-**2e**.

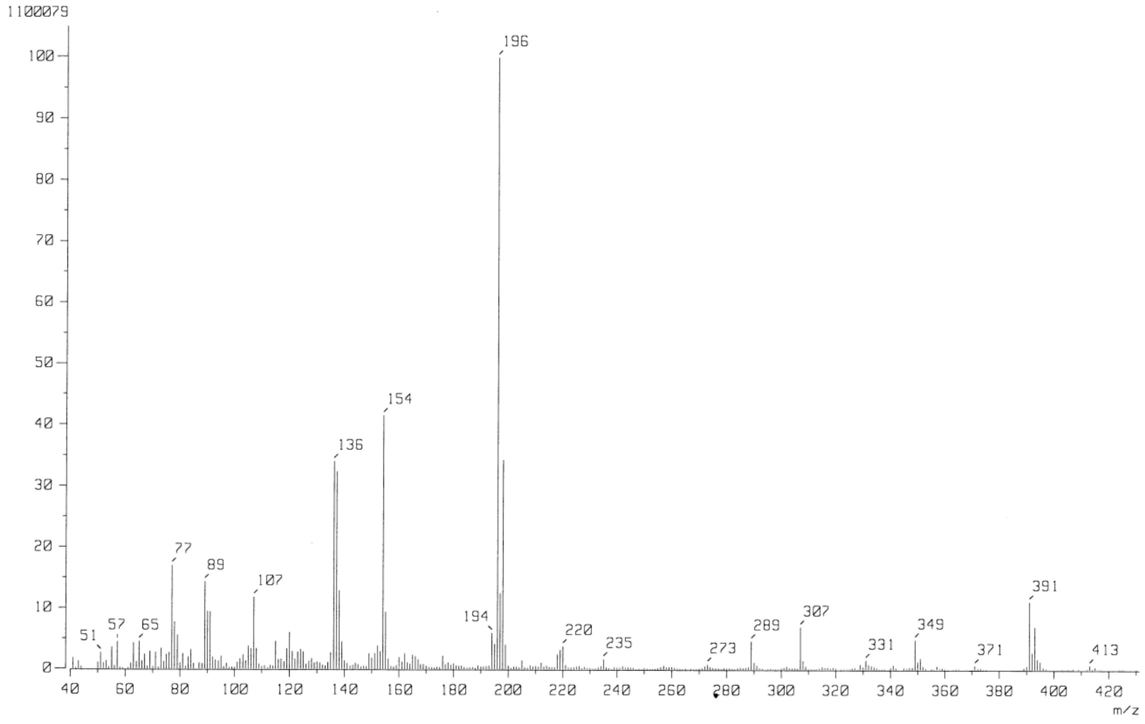

Figure S40. HRMS of 4-(2-Chlorophenyl)-pyrrolidin-2-one (±)-**2e**.

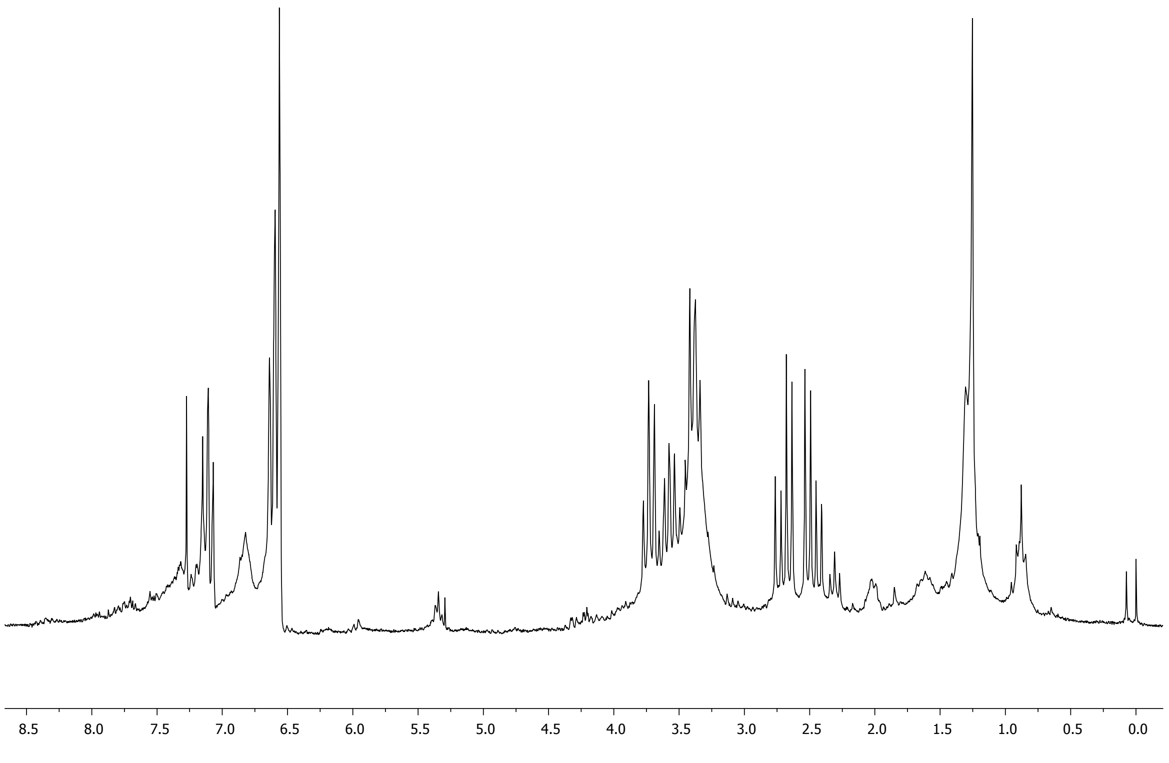

Figure S41. ^{1}H-NMR of 4-(2-Aminophenyl)-pyrrolidin-2-one (±)-**2f**.

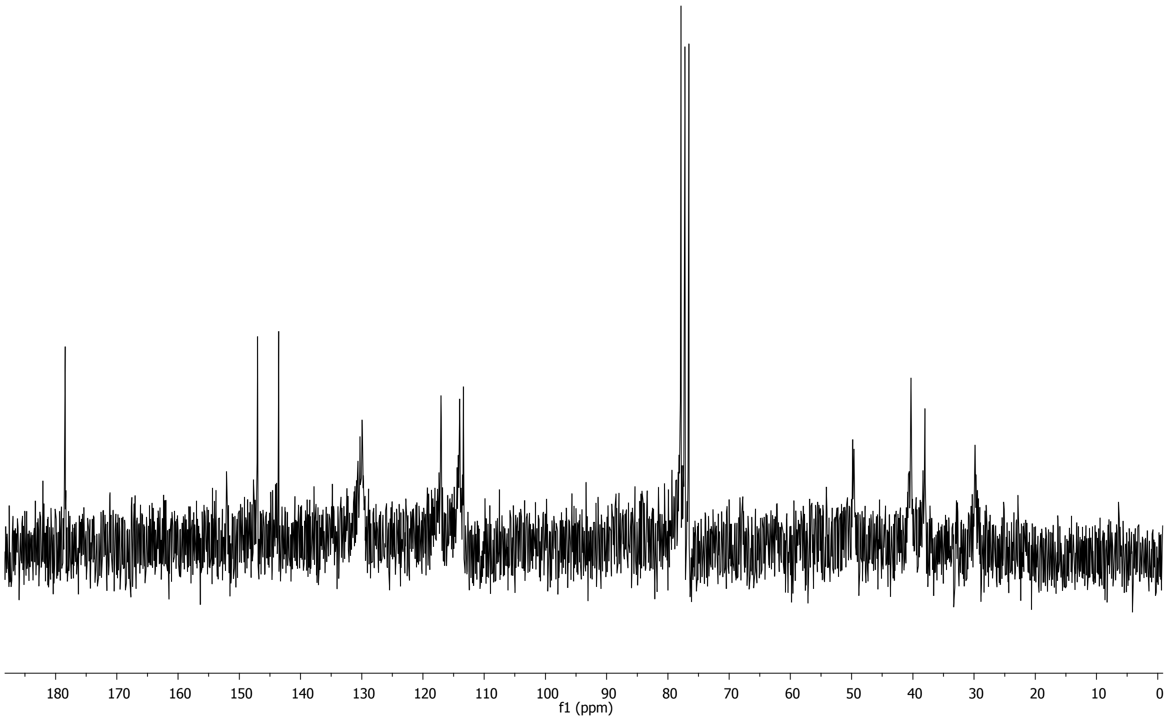

Figure S42. ^{13}C-NMR of 4-(2-Aminophenyl)-pyrrolidin-2-one (±)-**2f**.

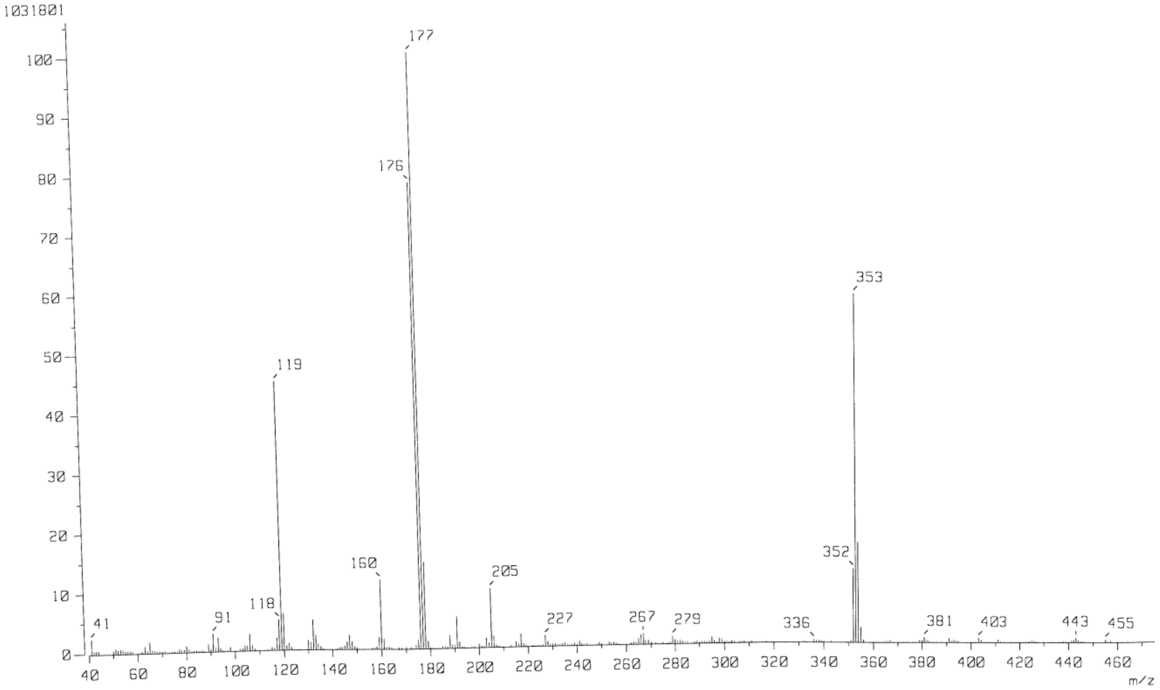

Figure S43. HRMS of 4-(2-Aminophenyl)-pyrrolidin-2-one (±)-**2f**.

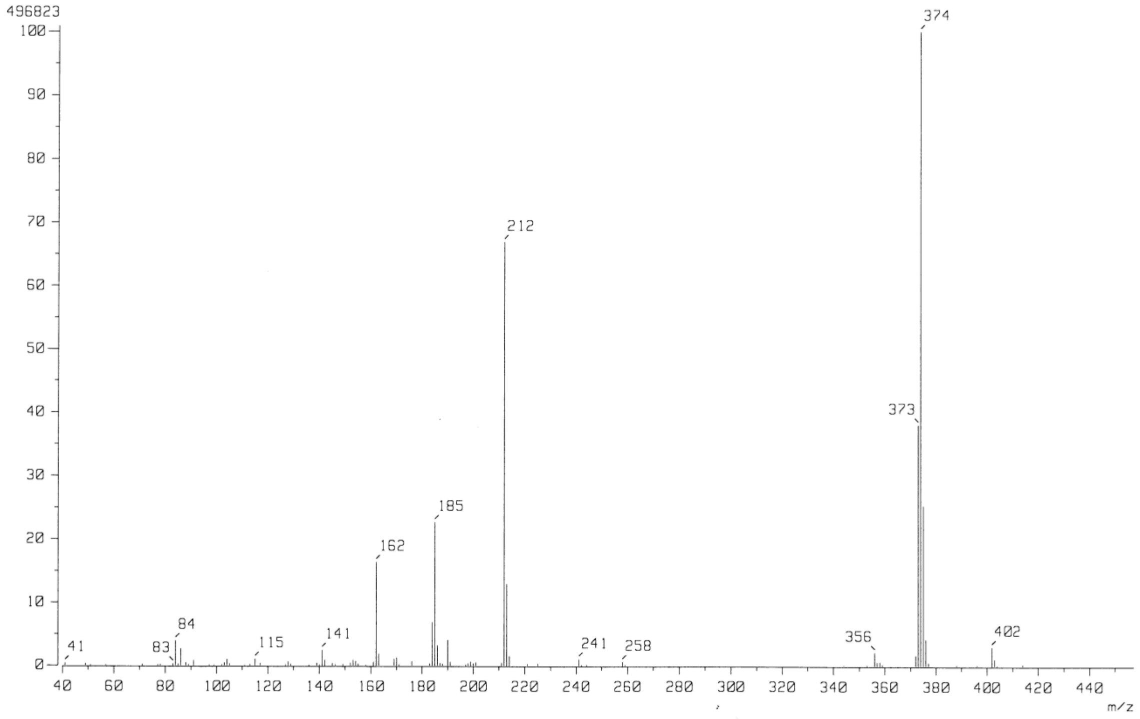

Figure S44. HRMS of 4-(2-Aminophenyl)-pyrrolidin-2-one (±)-**2f**.

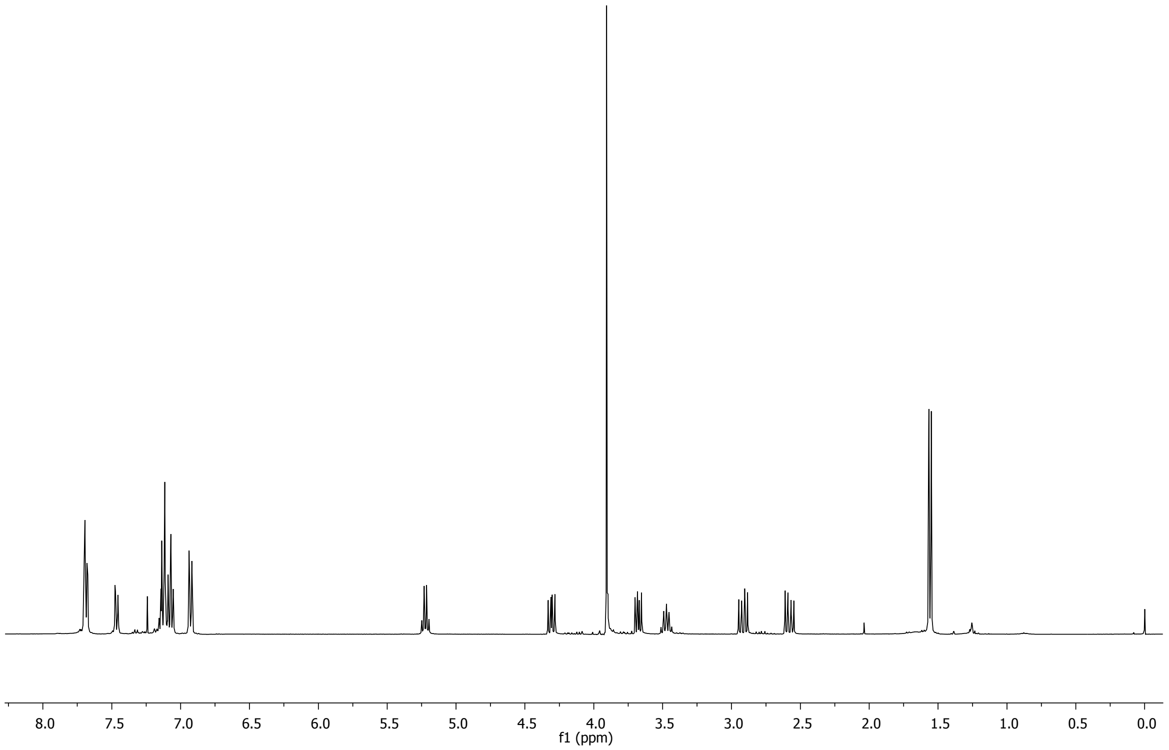

Figure S45. ^{1}H-NMR of (*R*)-1-((*S*)-2(6-Methoxynaphth-2-yl)propionyl)-4-phenyl-pyrrolidin-2-one (*R,S*)-**10a.**

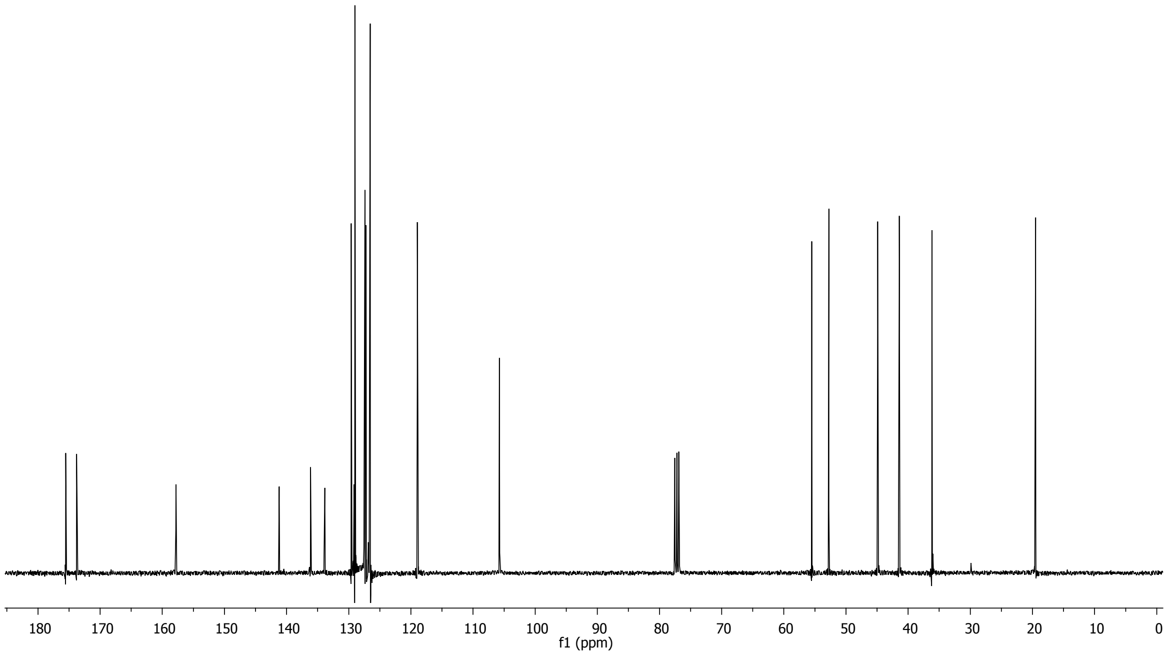

Figure S46. ^{13}C-NMR of (*R*)-1-((*S*)-2(6-Methoxynaphth-2-yl)propionyl)-4-phenyl-pyrrolidin-2-one (*R,S*)-**10a.**

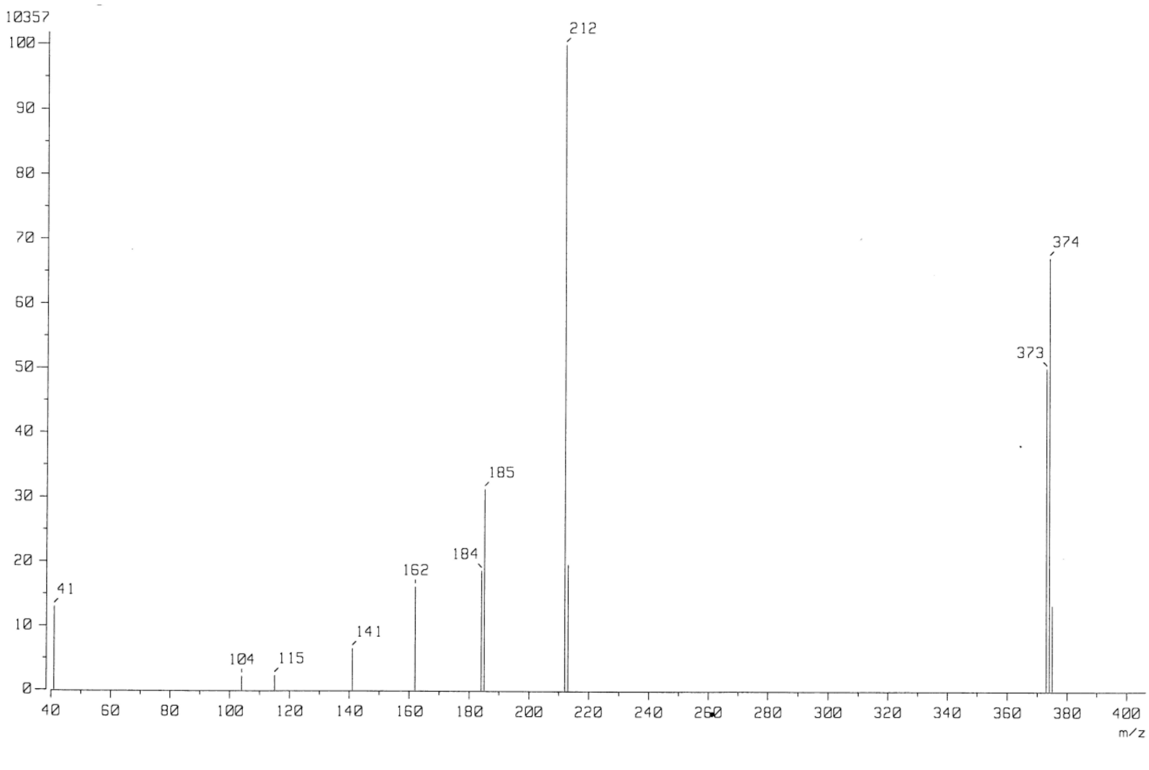

Figure S47. HRMS of (*R*)-1-((*S*)-2(6-Methoxynaphth-2-yl)propionyl)-4-phenyl-pyrrolidin-2-one (*R,S*)-**10a.**

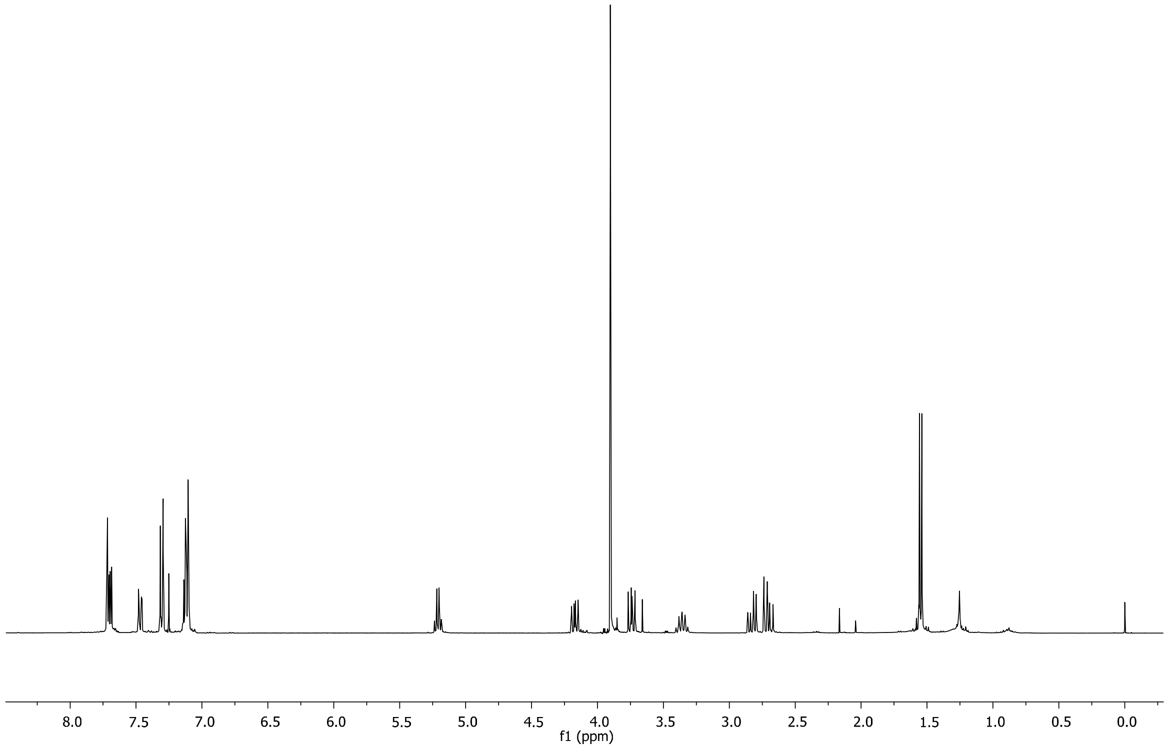

Figure S48. ^{1}H-NMR of ((*R*)-4-(4-Chlorophenyl)-1-((*S*)-2-(6-methoxynaphth-2-yl)propionyl)-pyrrolidin-2-one (*R,S*)-**10b**.

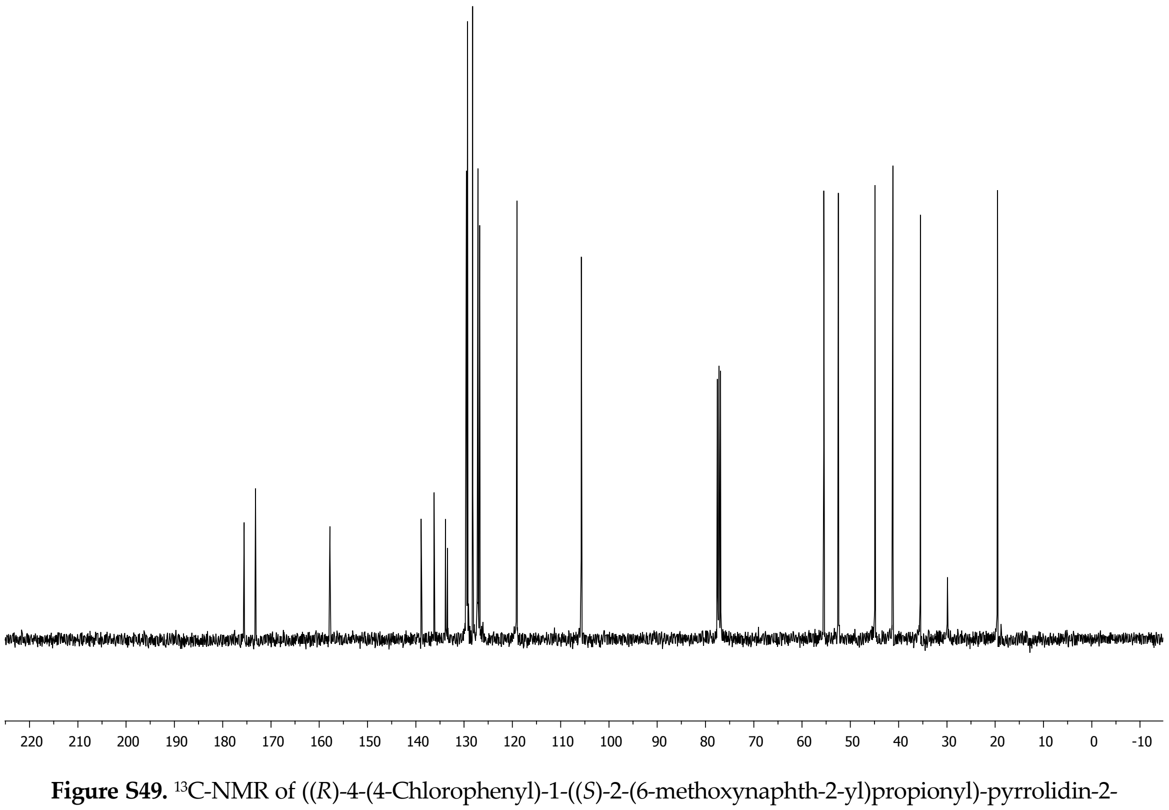

Figure S49. ^{13}C-NMR of ((*R*)-4-(4-Chlorophenyl)-1-((*S*)-2-(6-methoxynaphth-2-yl)propionyl)-pyrrolidin-2-one (*R,S*)-**10b**.

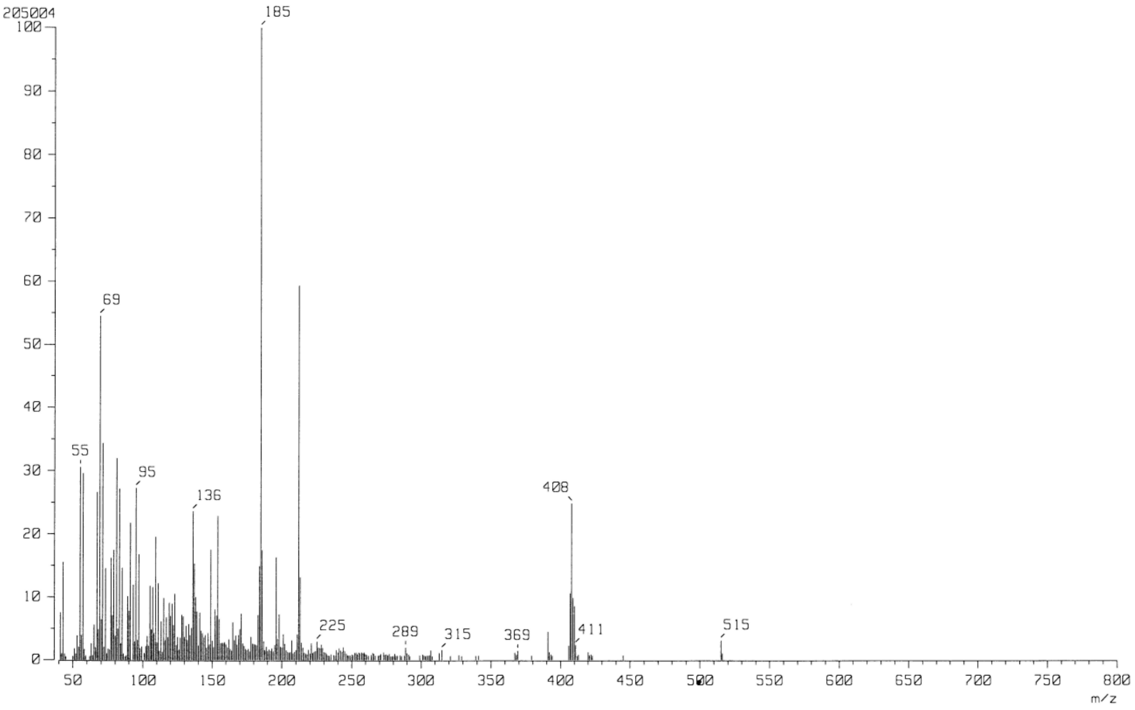

Figure S50. HRMS of ((*R*)-4-(4-Chlorophenyl)-1-((*S*)-2-(6-methoxynaphth-2-yl)propionyl)-pyrrolidin-2-one (*R,S*)-**10b**.

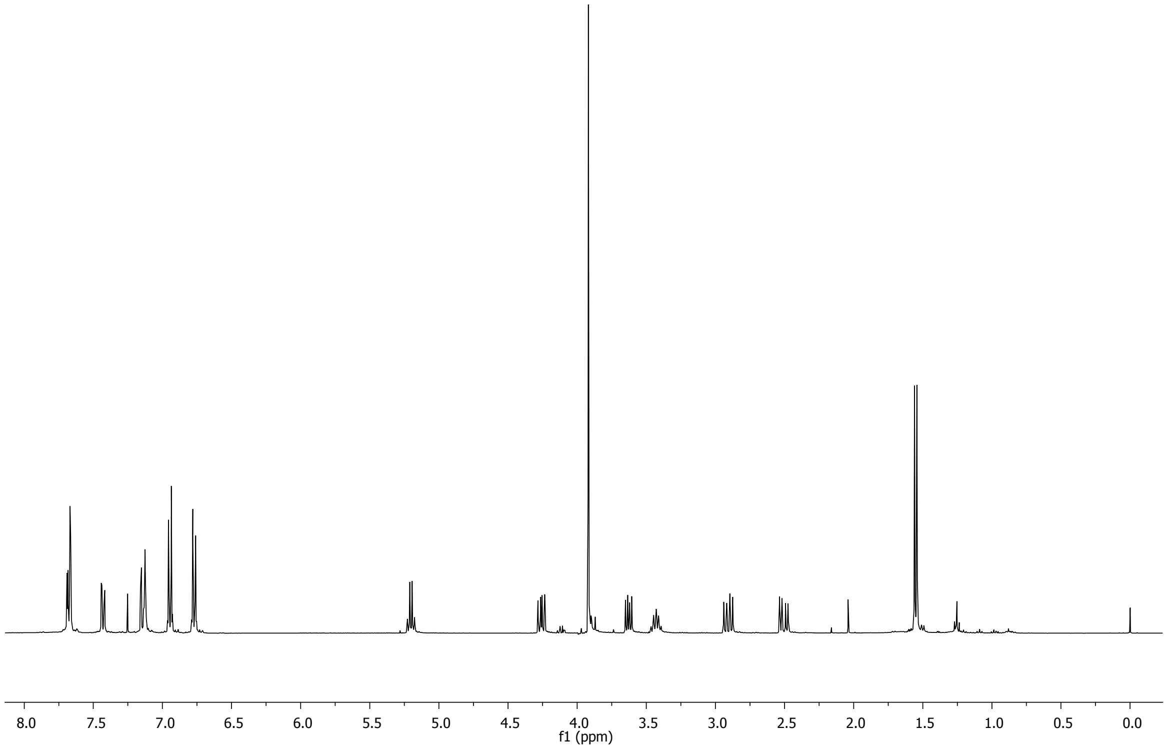

Figure S51. ^{1}H-NMR of (*S*)-4-(4-chlorophenyl)-1-((*S*)-2-(6-methoxynaphth-2-yl)propionyl)-pyrrolidin-2-one (*S,S*)-**10b.**

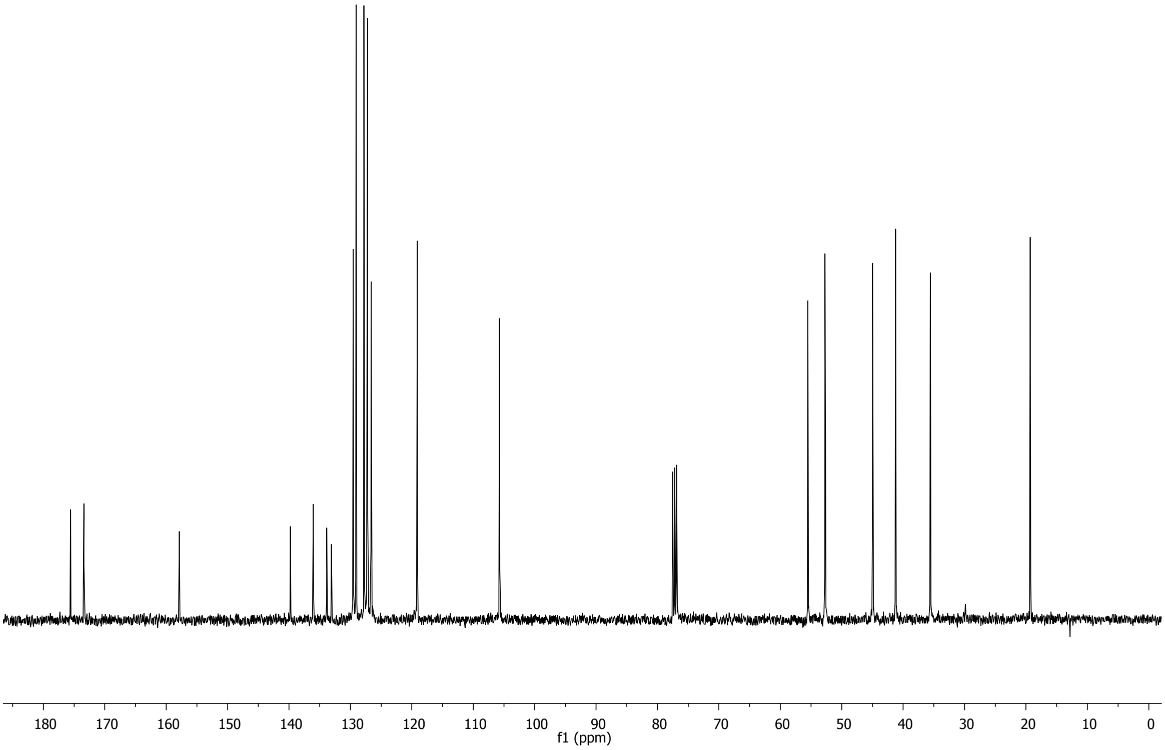

Figure S52. ^{13}C-NMR of (*S*)-4-(4-chlorophenyl)-1-((*S*)-2-(6-methoxynaphth-2-yl)propionyl)-pyrrolidin-2-one (*S,S*)-**10b.**

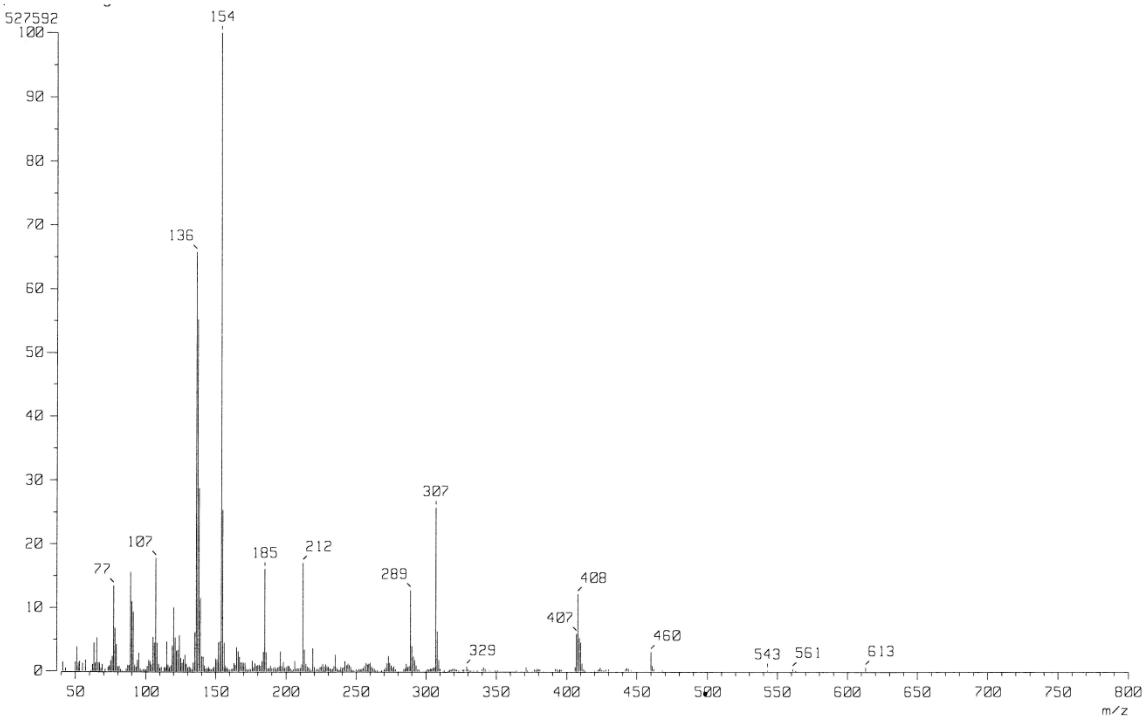

Figure S53. HRMS of (*S*)-4-(4-chlorophenyl)-1-((*S*)-2-(6-methoxynaphth-2-yl)propionyl)-pyrrolidin-2-one (*S,S*)-**10b.**

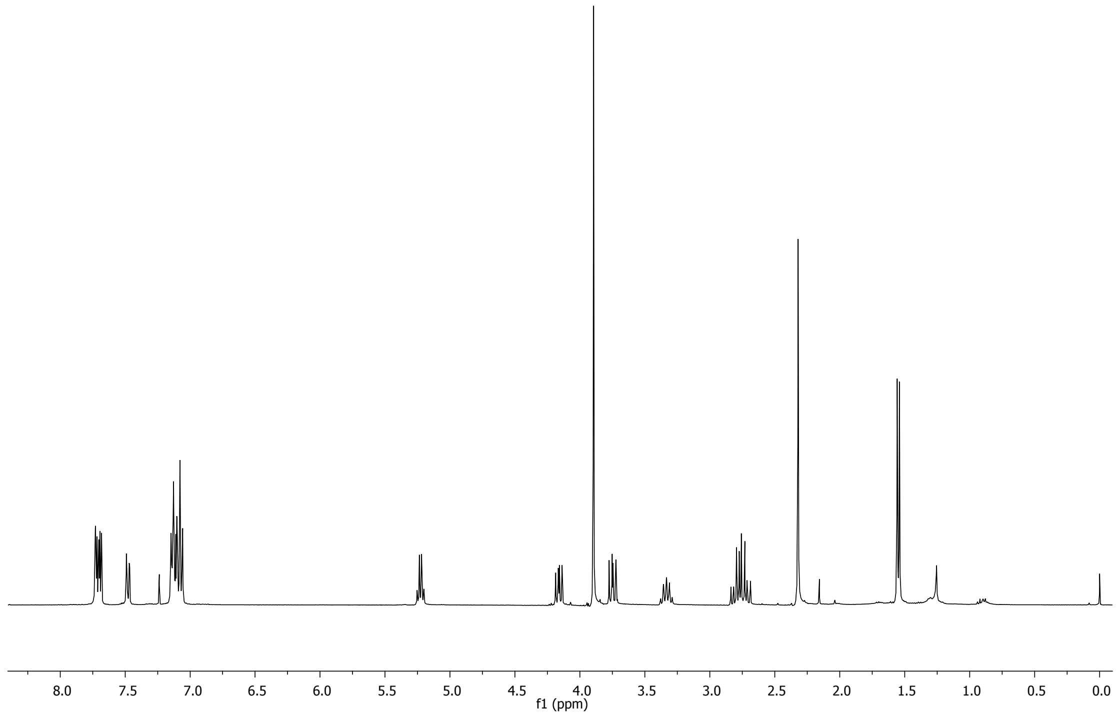

Figure S54. ^{1}H-NMR of (*R*)-1-((*S*)-2-(6-Methoxynaphth-2-yl)propionyl-4-(4-methylphenyl)-pyrrolidin-2-one (*R,S*)-**10c.**

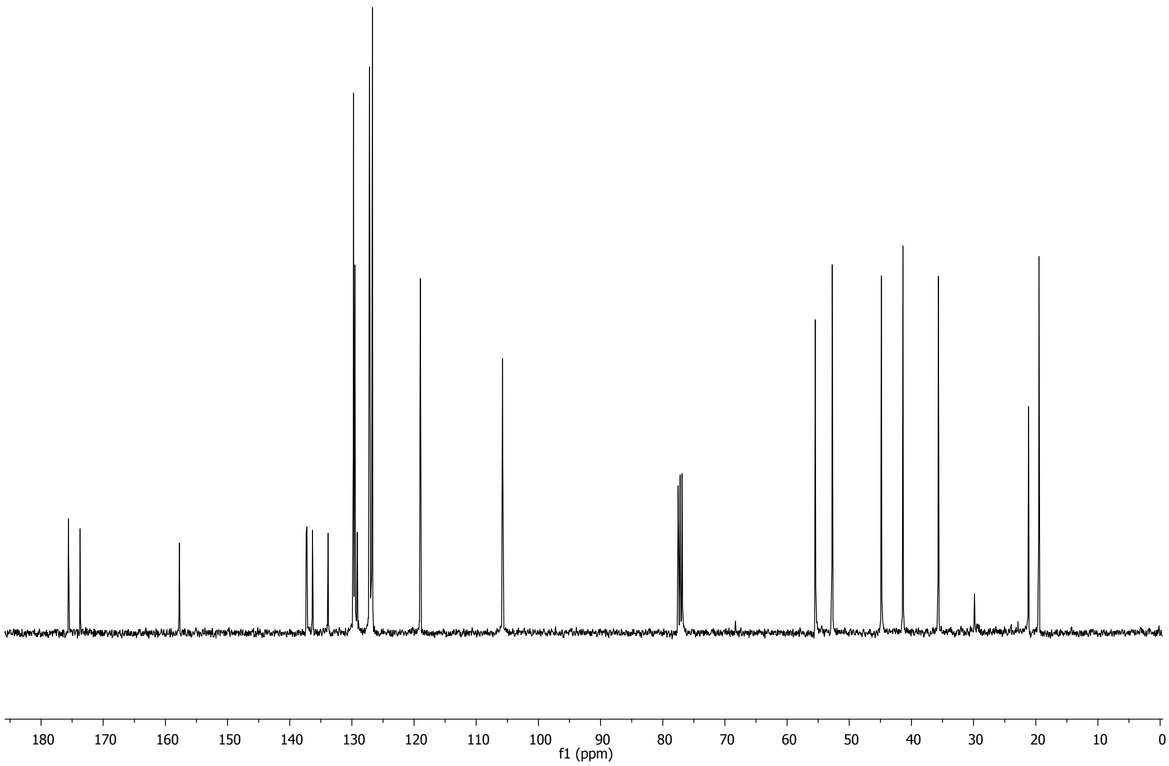

Figure S55. ^{13}C-NMR of (*R*)-1-((*S*)-2-(6-Methoxynaphth-2-yl)propionyl-4-(4-methylphenyl)-pyrrolidin-2-one (*R,S*)-**10c.**

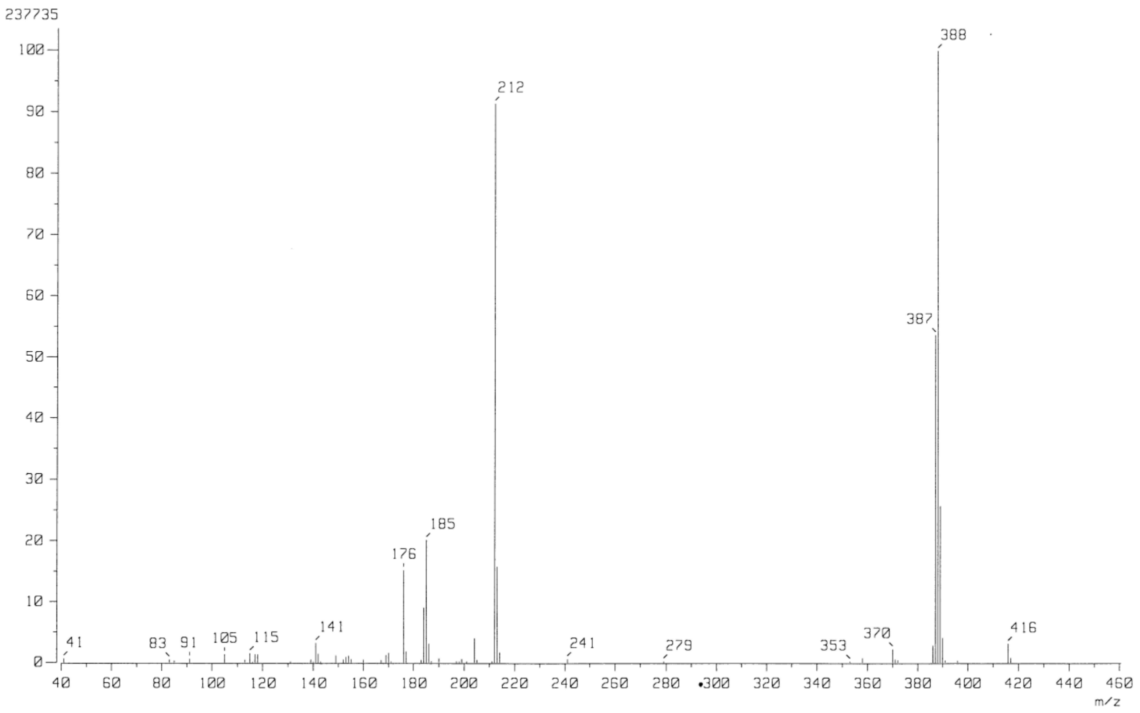

Figure S56. HRMS of (*R*)-1-((*S*)-2-(6-Methoxynaphth-2-yl)propionyl-4-(4-methylphenyl)-pyrrolidin-2-one (*R,S*)-**10c.**

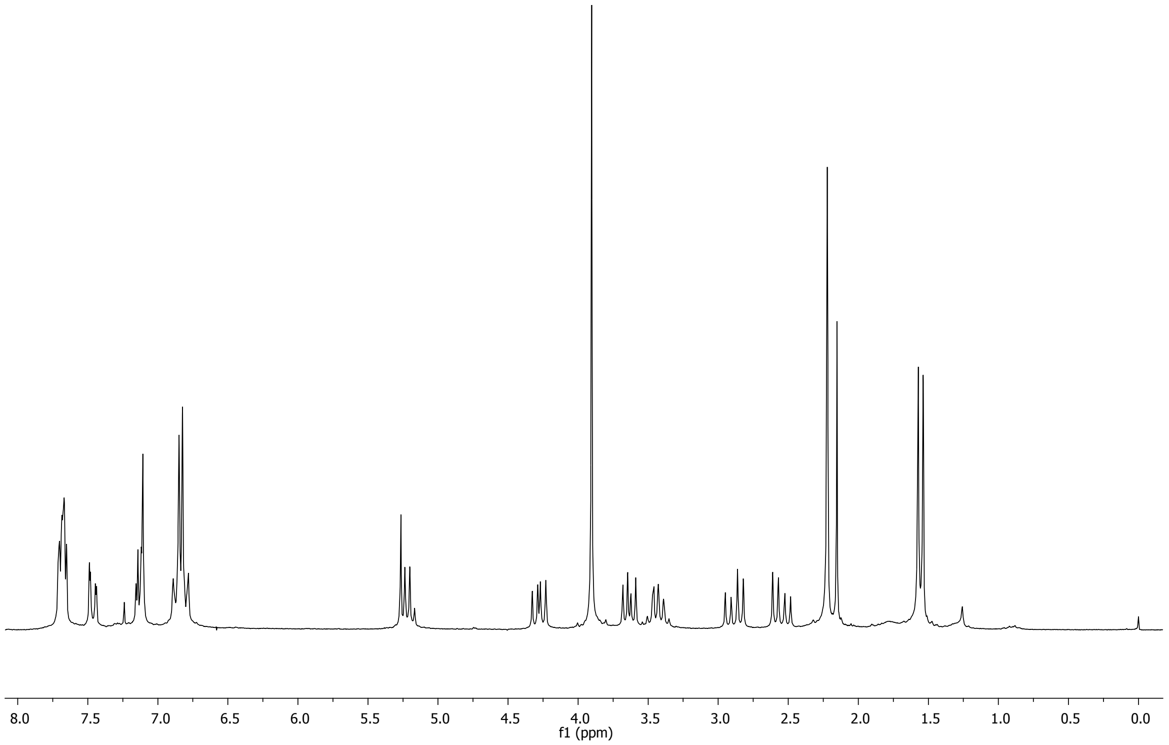

Figure S57. ^{1}H-NMR of (*S*)-1-((*S*)-2-(6-methoxynaphth-2-yl)propionyl-4-(4-methylphenyl)-pyrrolidin-2-one (*S,S*)-**10c**.

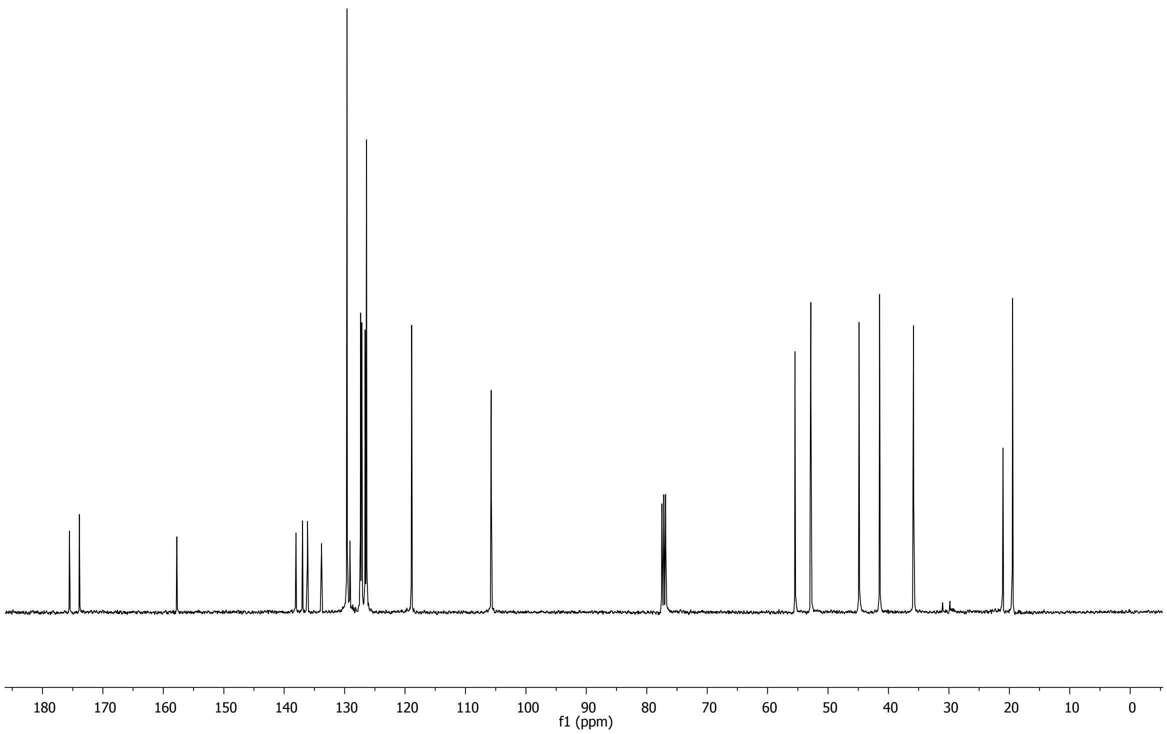

Figure S58. ^{13}C-NMR of (*S*)-1-((*S*)-2-(6-methoxynaphth-2-yl)propionyl-4-(4-methylphenyl)-pyrrolidin-2-one (*S,S*)-**10c**.

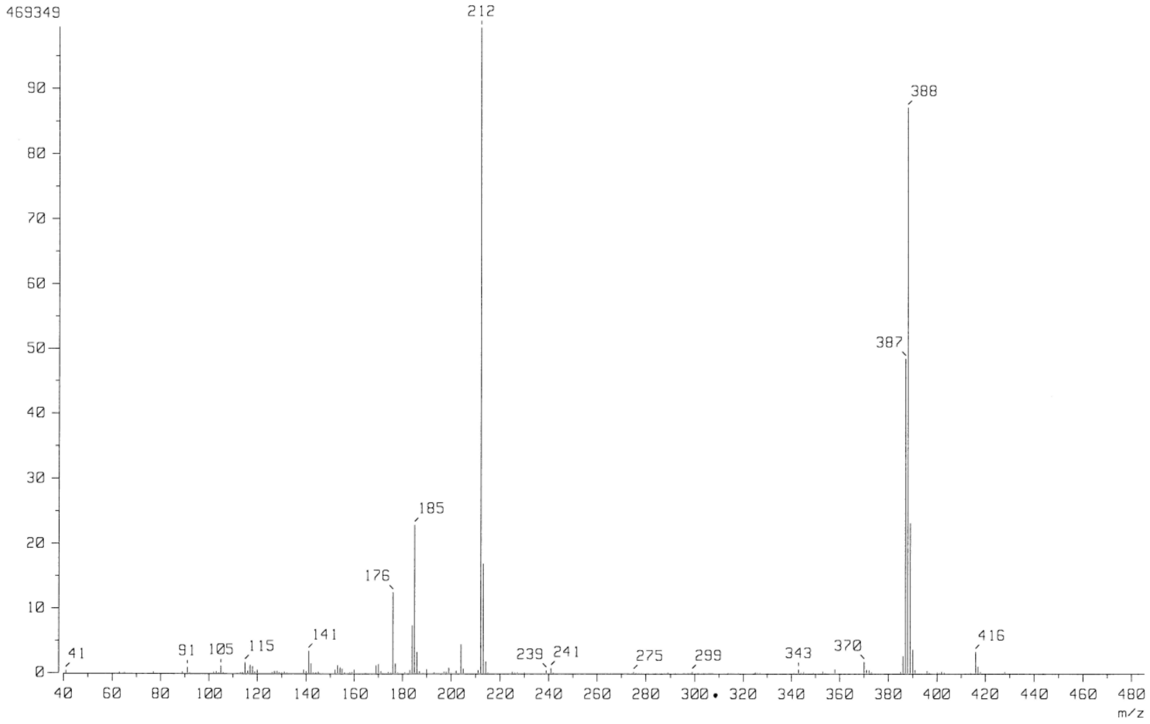

Figure S59. HRMS of (*S*)-1-((*S*)-2-(6-methoxynaphth-2-yl)propionyl-4-(4-methylphenyl)-pyrrolidin-2-one (*S,S*)-**10c**.

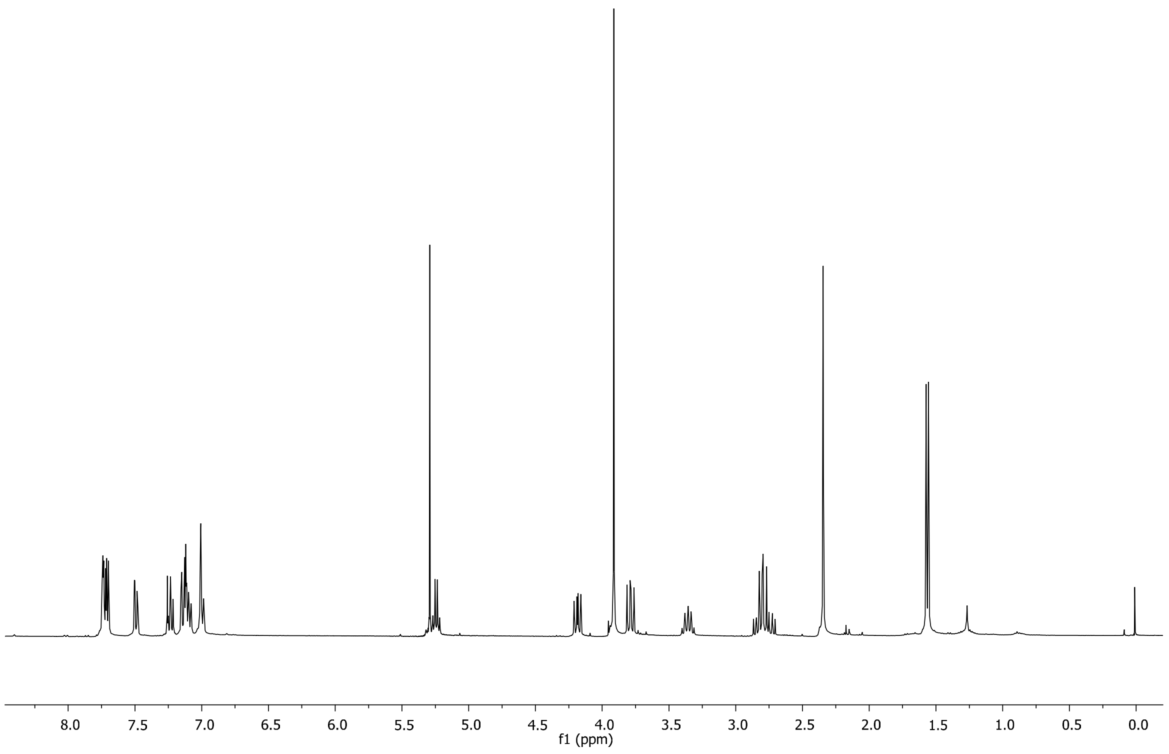

Figure S60. ^{1}H-NMR of (*R*)-1-((*S*)-2-(6-Methoxynaphth-2-yl)propionyl)-4-(3-methylphenyl)-pyrrolidin-2-one (*R,S*)-**10d.**

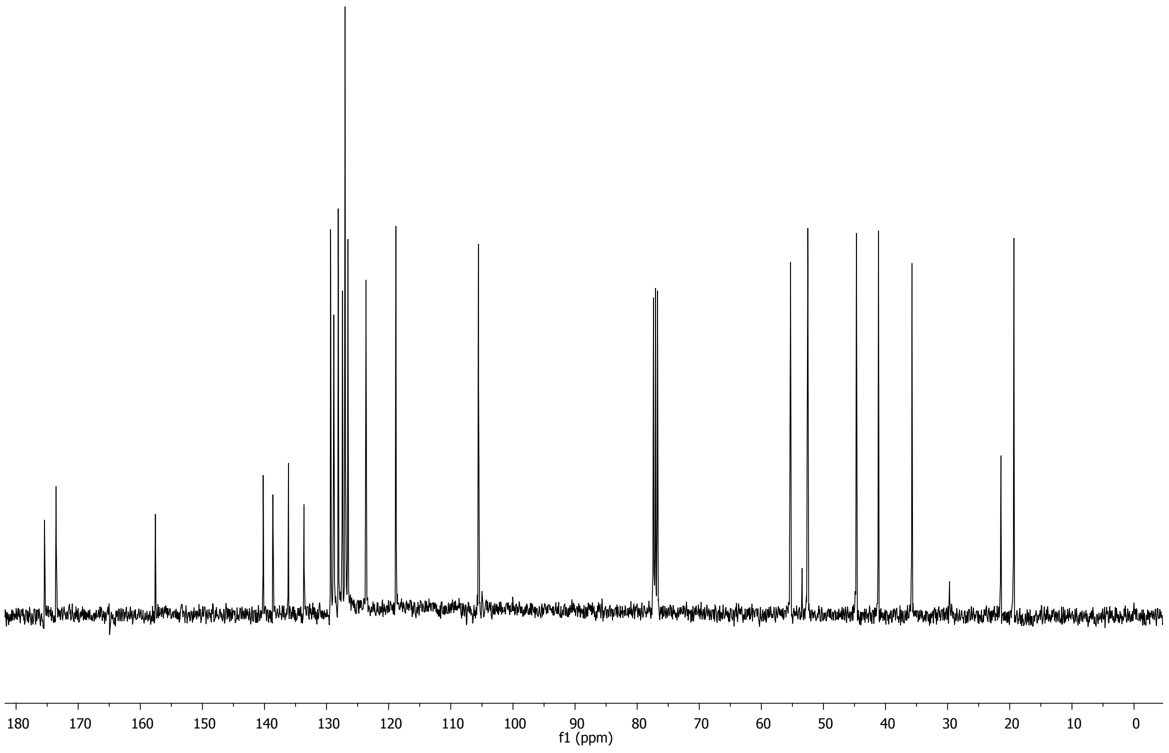

Figure S61. ^{1}H-NMR of (*R*)-1-((*S*)-2-(6-Methoxynaphth-2-yl)propionyl)-4-(3-methylphenyl)-pyrrolidin-2-one (*R,S*)-**10d.**

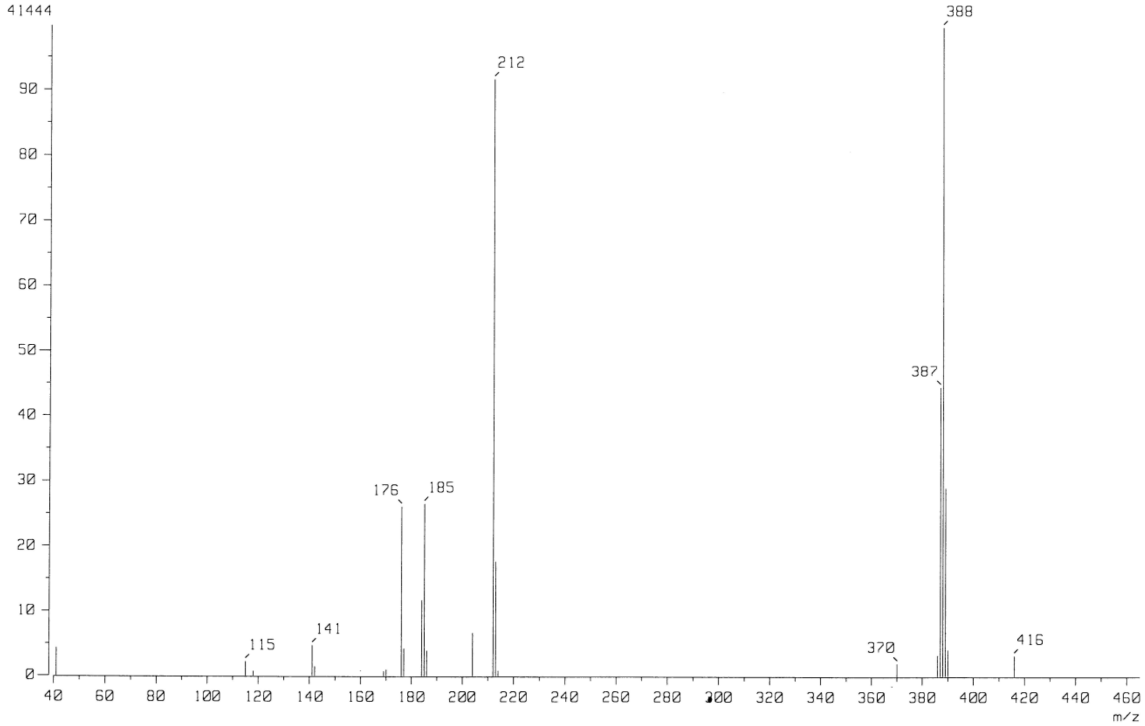

Figure S62. HRMS of (*R*)-1-((*S*)-2-(6-Methoxynaphth-2-yl)propionyl)-4-(3-methylphenyl)-pyrrolidin-2-one (*R,S*)-**10d.**

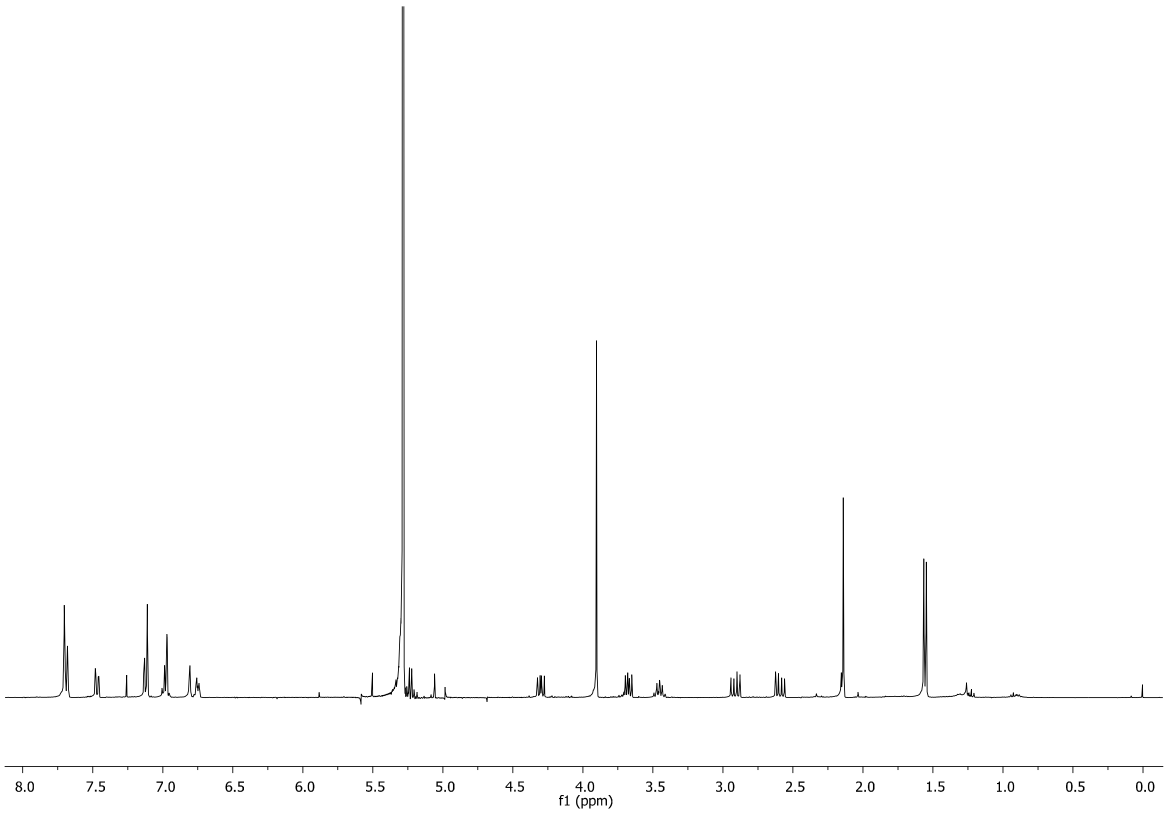

Figure S63. ^{1}H-NMR of (*S*)-1-((*S*)-2-(6-Methoxynaphth-2-yl)propionyl)-4-(3-methylphenyl)-pyrrolidin-2-one (*S,S*)-**10d**.

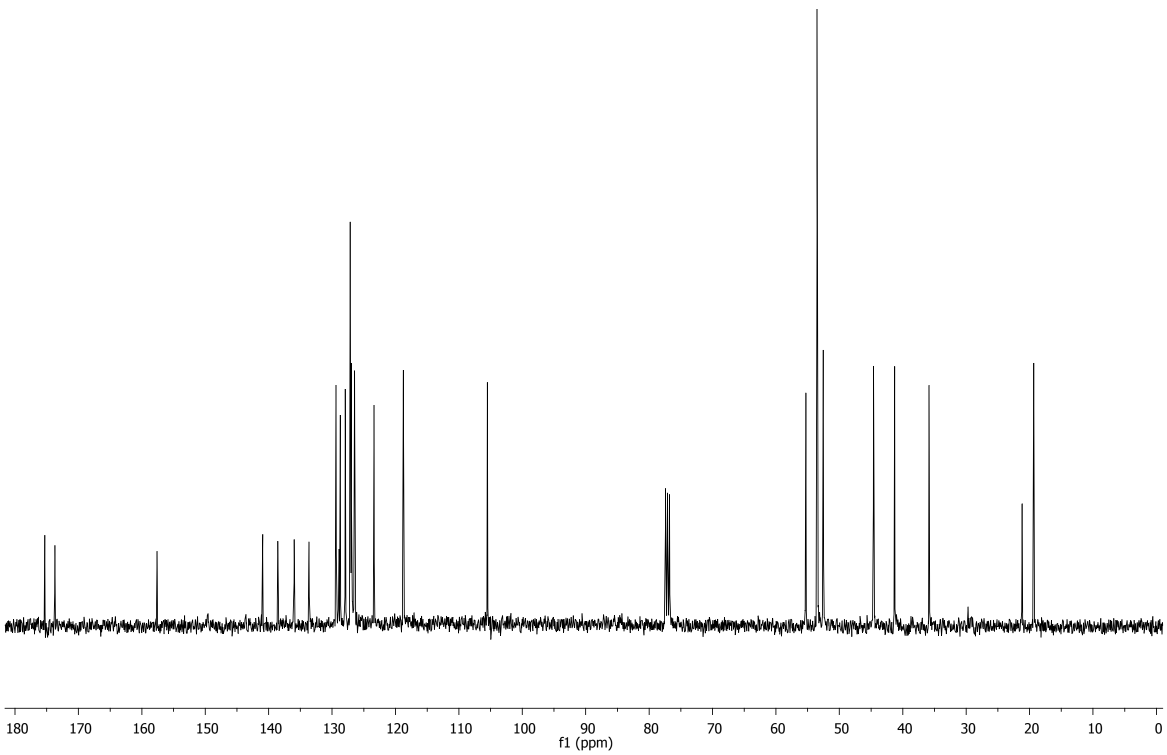

Figure S64. ^{13}C-NMR of (*S*)-1-((*S*)-2-(6-Methoxynaphth-2-yl)propionyl)-4-(3-methylphenyl)-pyrrolidin-2-one (*S,S*)-**10d**.

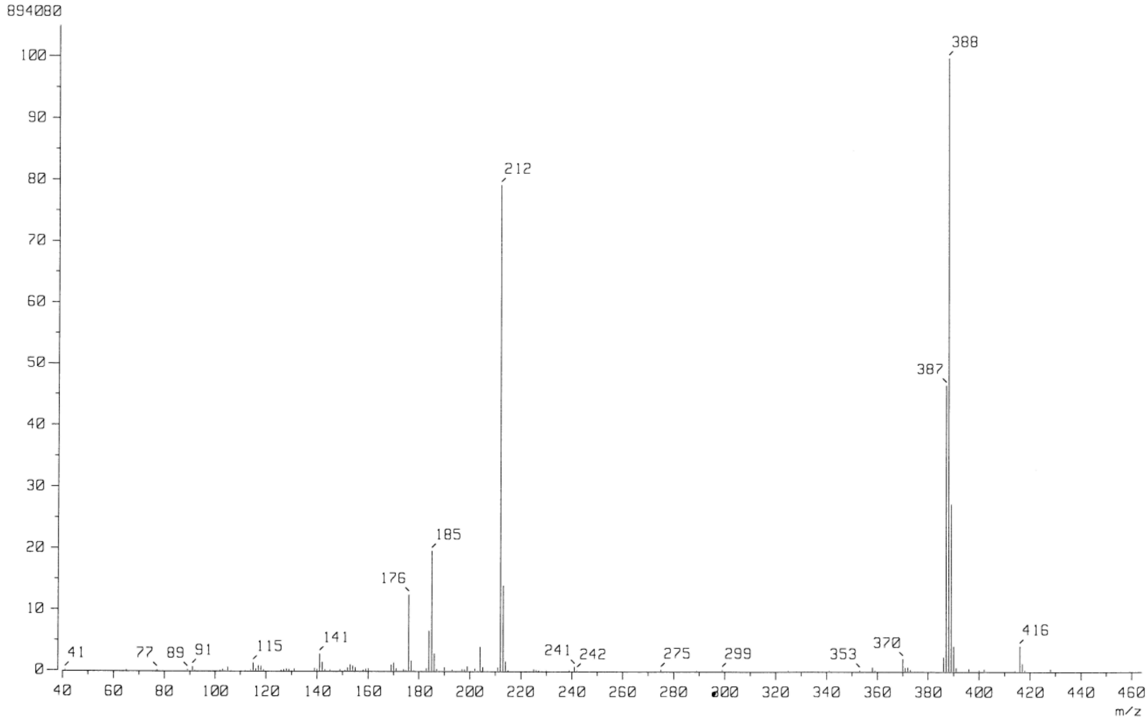

Figure S65. HRMS of (*S*)-1-((*S*)-2-(6-Methoxynaphth-2-yl)propionyl)-4-(3-methylphenyl)-pyrrolidin-2-one (*S,S*)-**10d**.

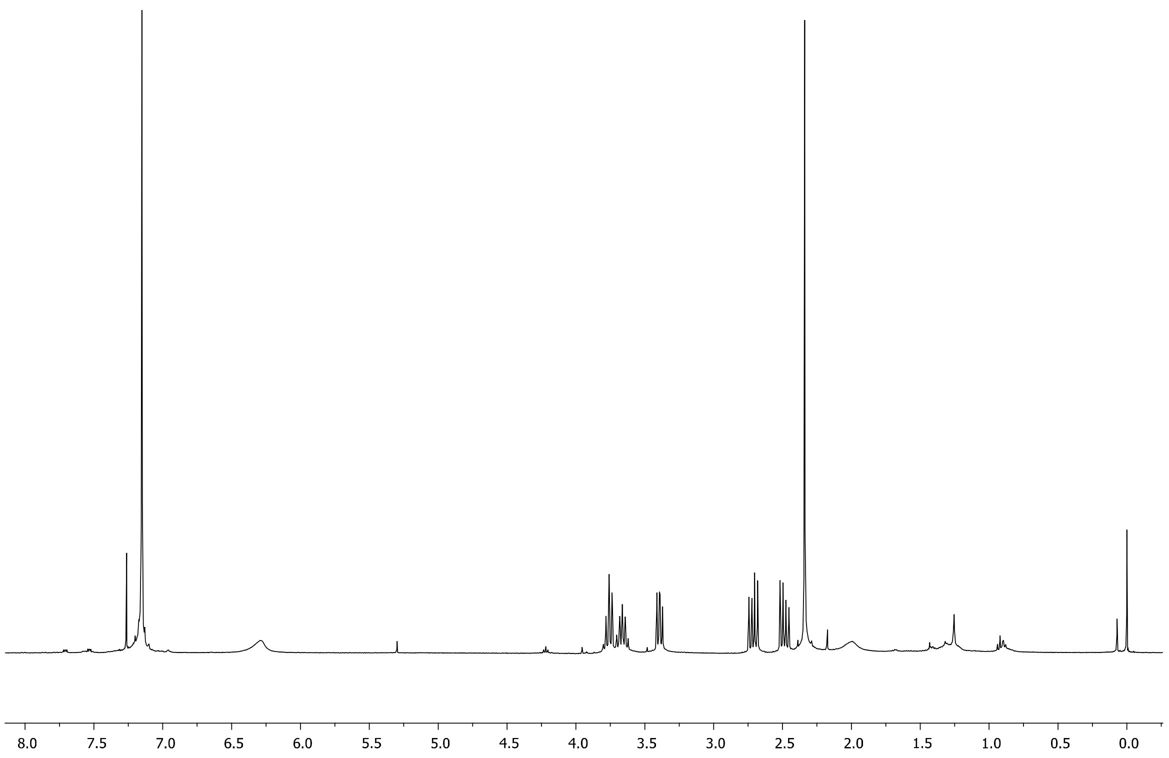

Figure S66. ^{1}H-NMR of (*R*)-4-(4-Methylphenyl)-pyrrolidin-2-one **2c**.

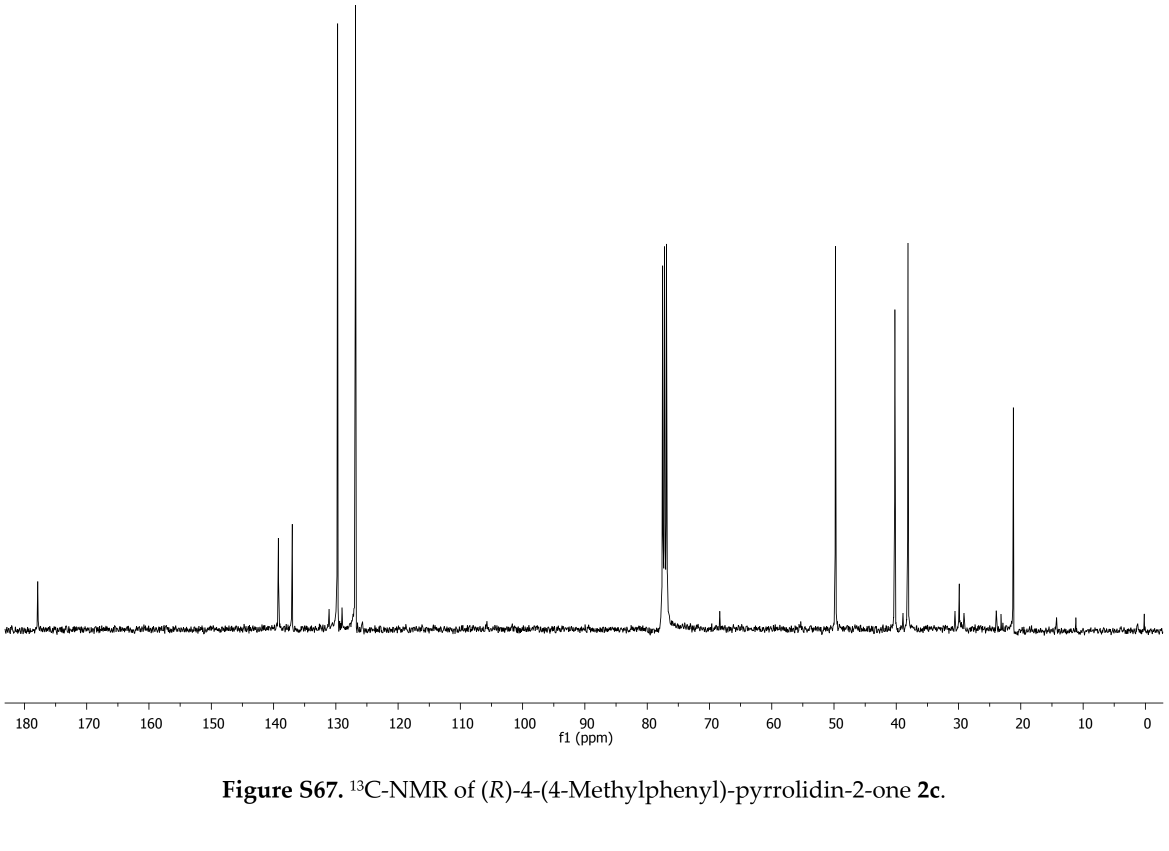

Figure S67. ^{13}C-NMR of (*R*)-4-(4-Methylphenyl)-pyrrolidin-2-one **2c**.

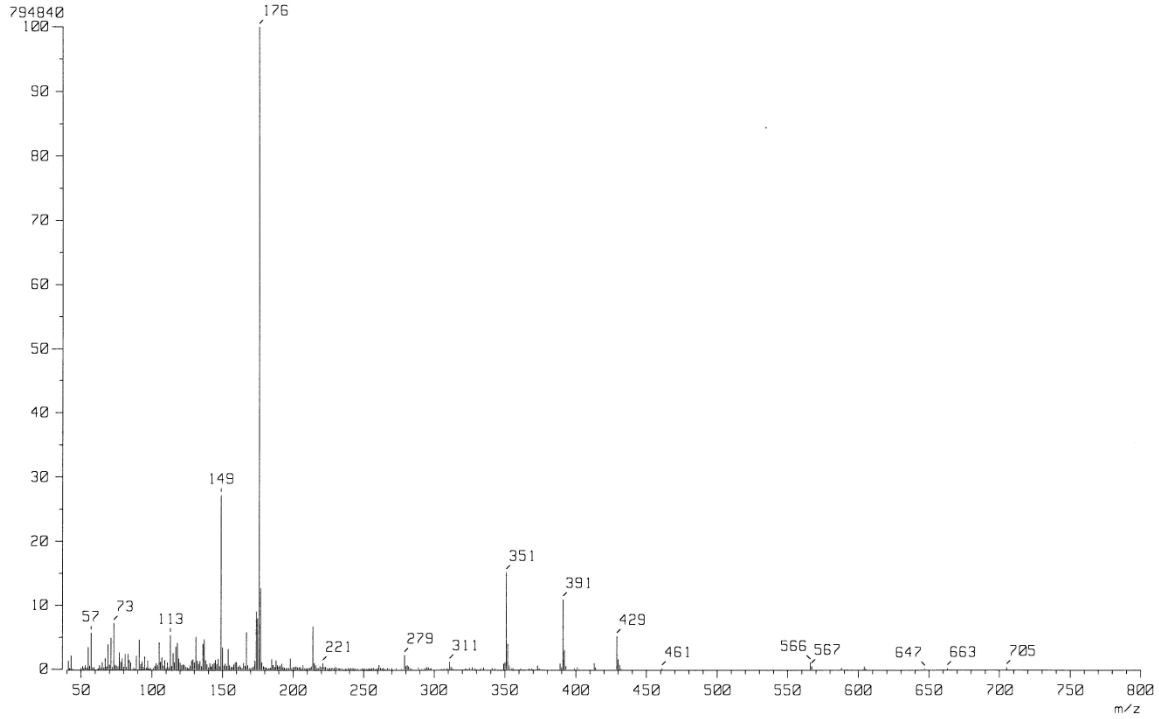

Figure S68. HRMS of (*R*)-4-(4-Methylphenyl)-pyrrolidin-2-one **2c**.

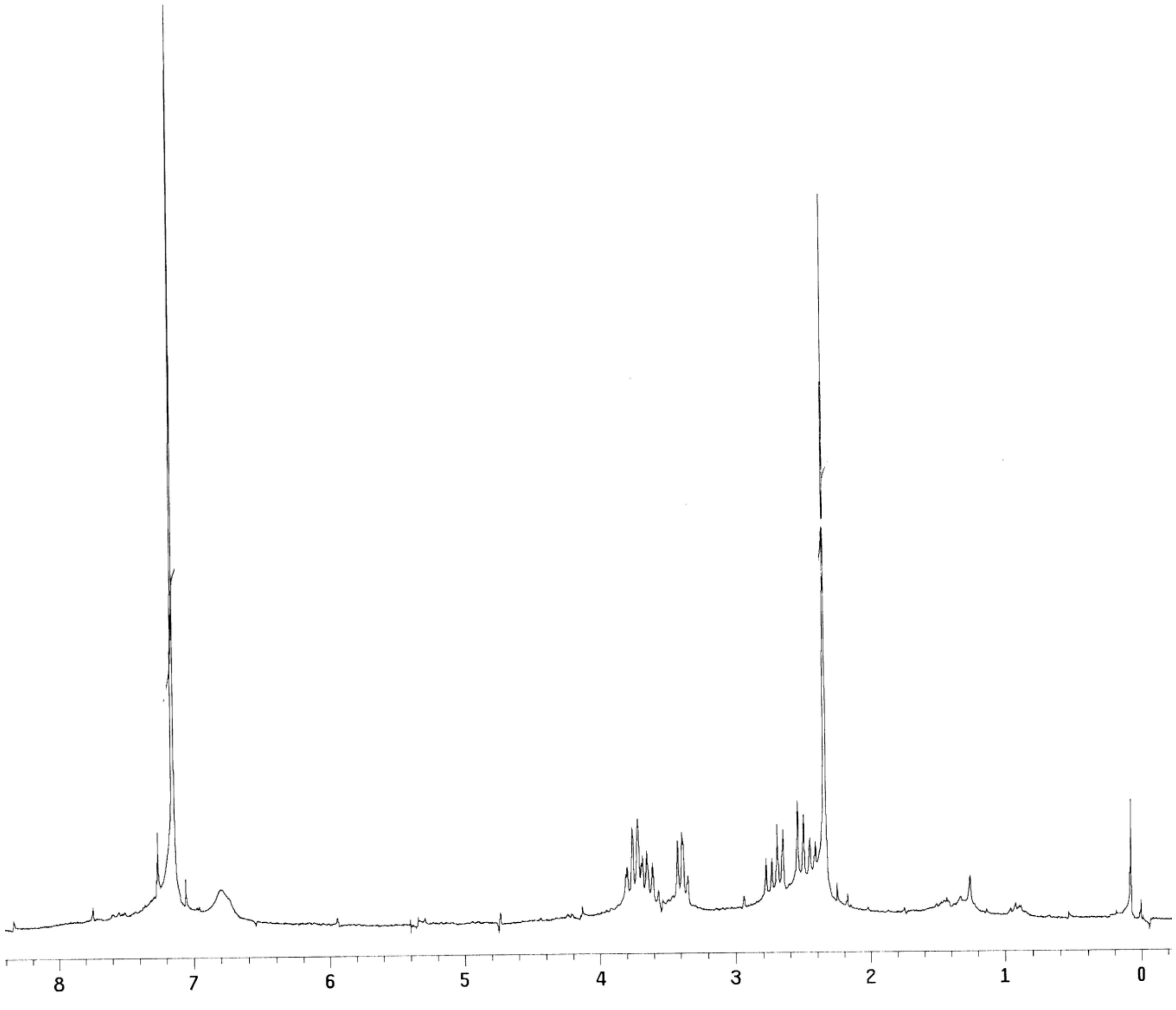

Figure S69. ^{1}H-NMR of (*S*)-4-(4-Methylphenyl)-pyrrolidin-2-one **2c**.

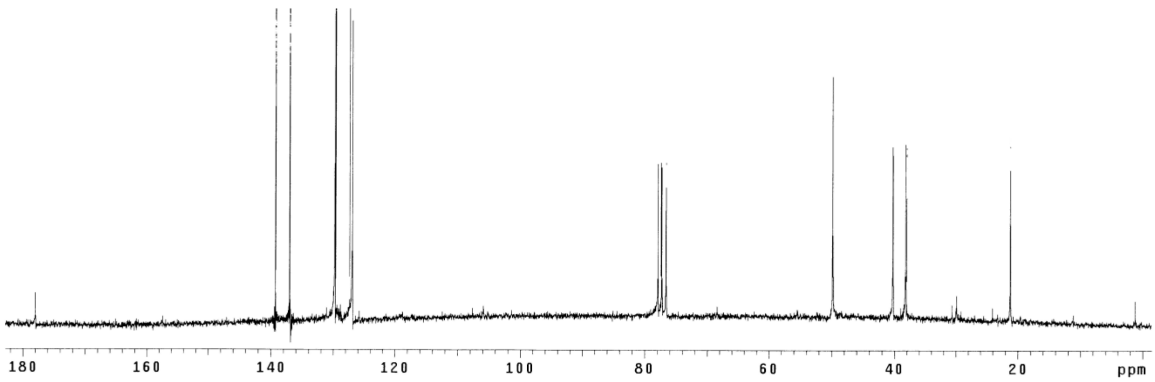

Figure S70. ^{13}C-NMR of (*S*)-4-(4-Methylphenyl)-pyrrolidin-2-one **2c**.

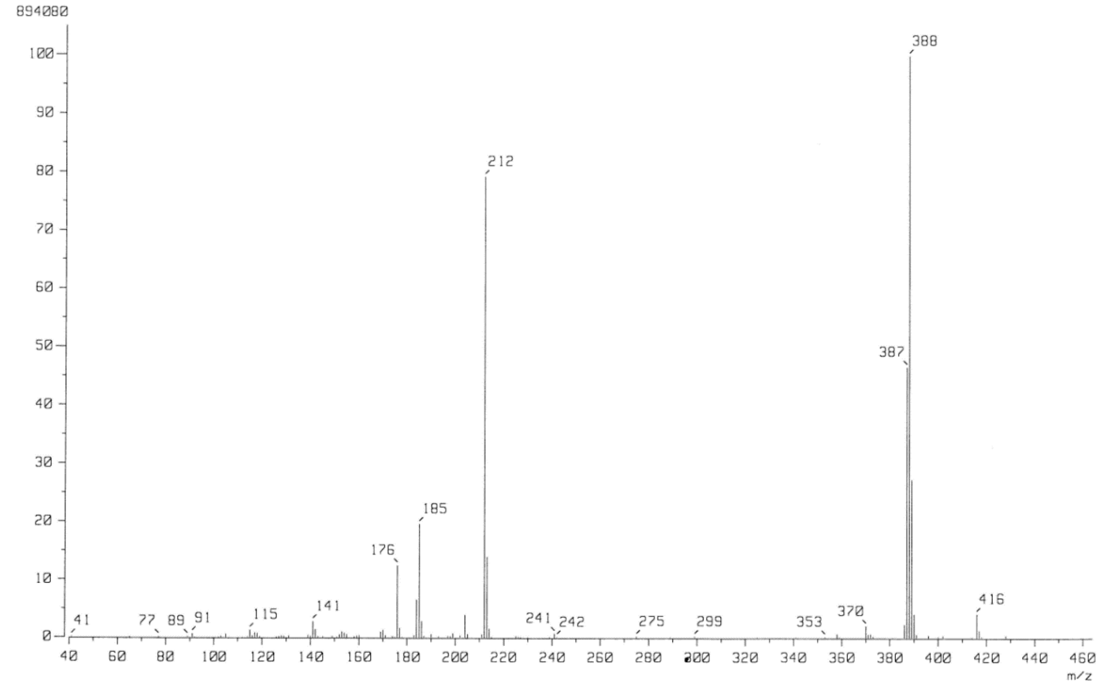

Figure S71. HRMS of (*S*)-4-(4-Methylphenyl)-pyrrolidin-2-one **2c**.

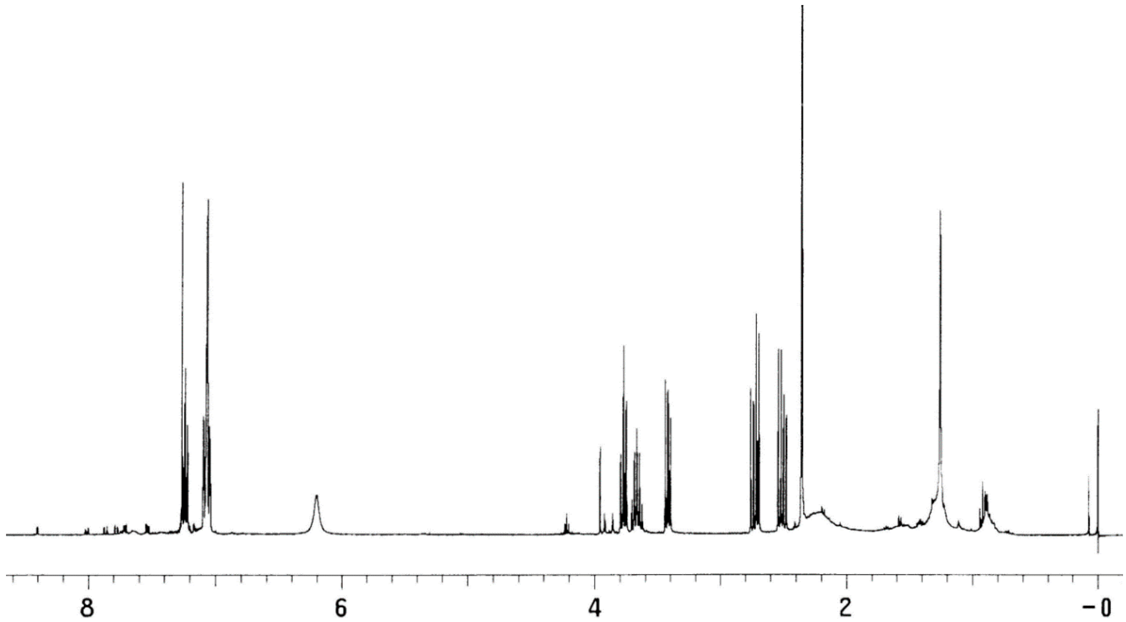

Figure S72. ^{1}H-NMR of (*R*)-4-(3-Methylphenyl)-pyrrolidin-2-one **2d**.

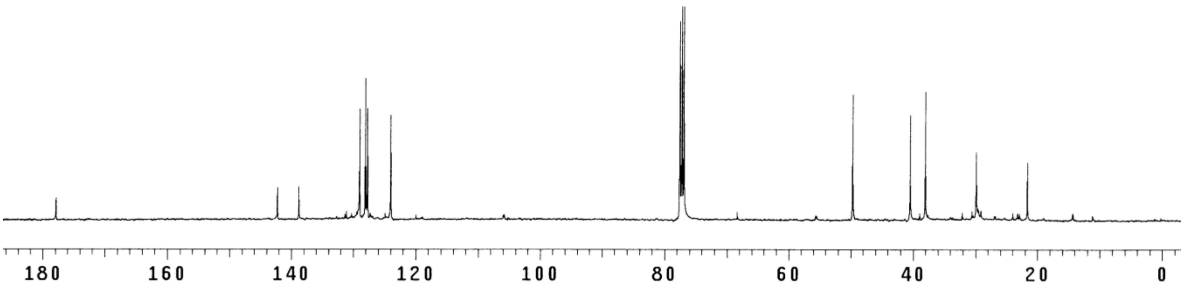

Figure S73. ^{1}H-NMR of (*R*)-4-(3-Methylphenyl)-pyrrolidin-2-one **2d**.

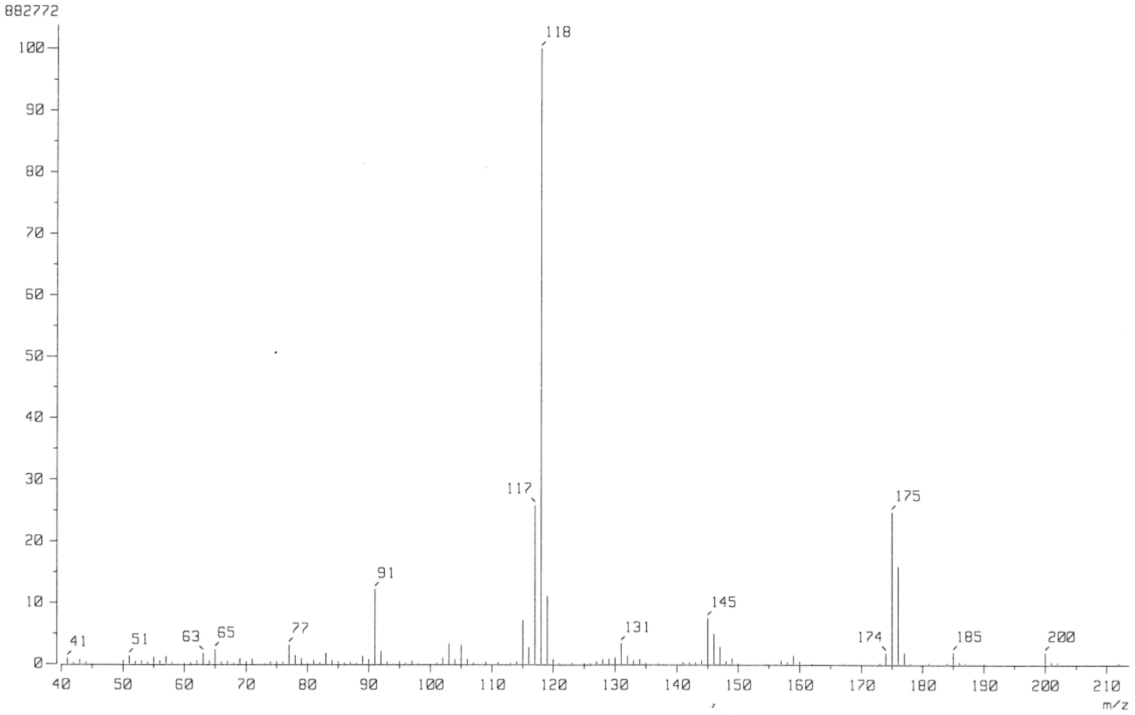

Figure S74. HRMS of (*R*)-4-(3-Methylphenyl)-pyrrolidin-2-one **2d**.

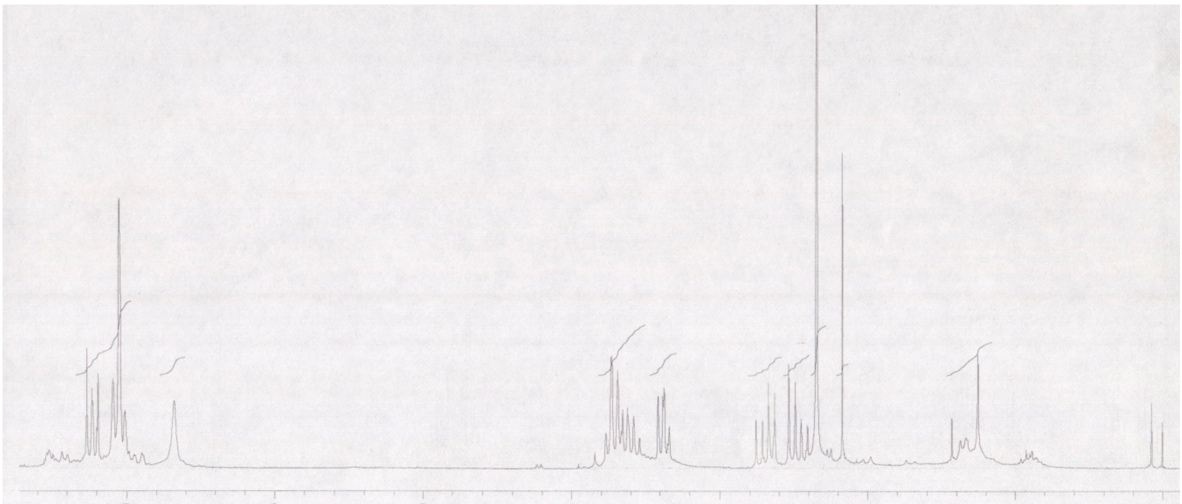

Figure S75. ^{1}H-NMR of (*S*)-4-(3-Methylphenyl)-pyrrolidin-2-one **2d**.

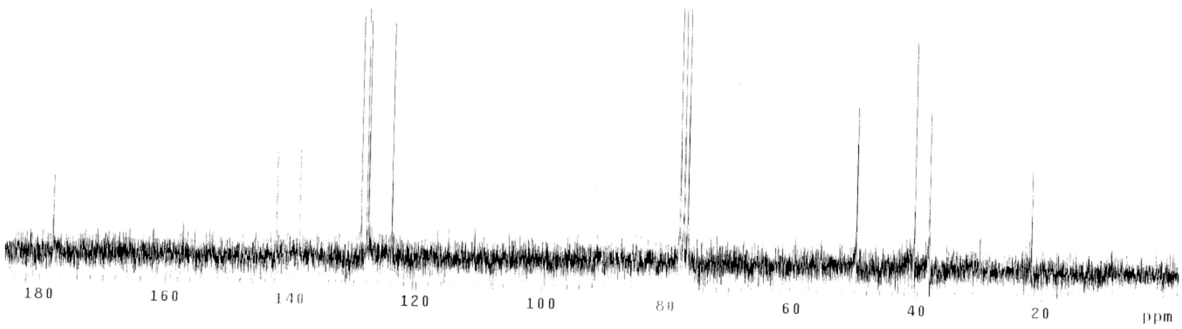

Figure S76. ^{13}C-NMR of (*S*)-4-(3-Methylphenyl)-pyrrolidin-2-one **2d**.

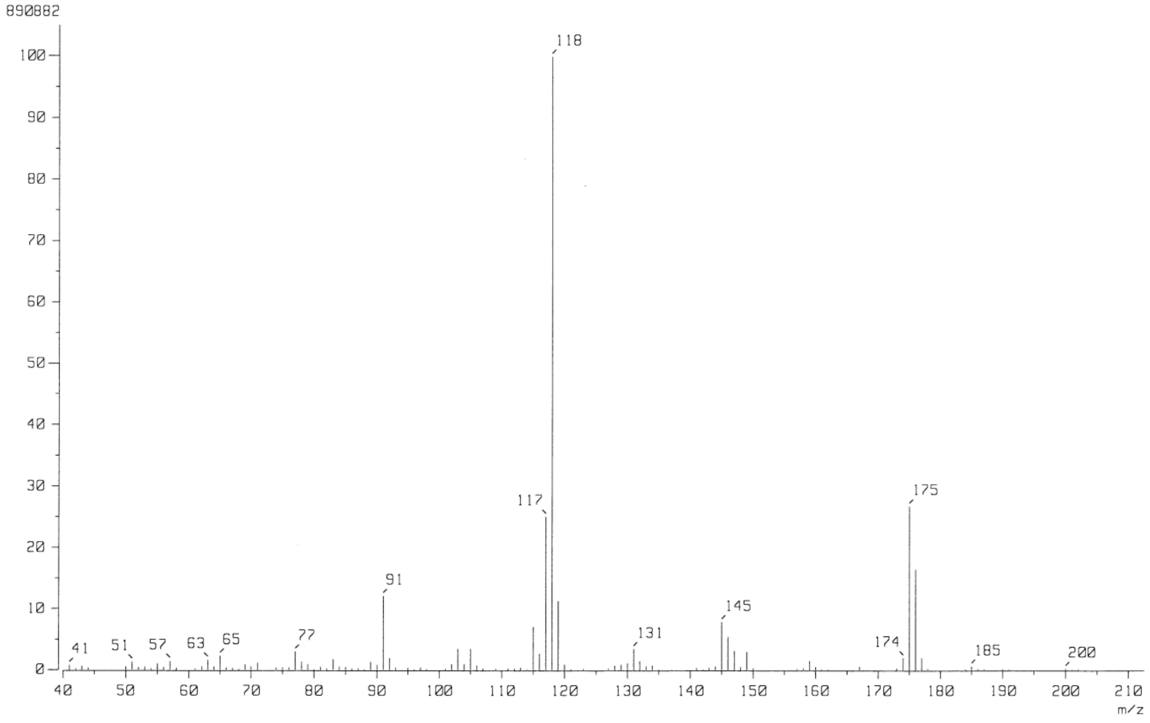

Figure S77. HRMS of (*S*)-4-(3-Methylphenyl)-pyrrolidin-2-one **2d**.
